# Synthesis of Novel Suramin Analogs With Anti-Proliferative Activity *via* FGF1 and FGFRD2 Blockade

**DOI:** 10.3389/fchem.2021.764200

**Published:** 2022-01-03

**Authors:** Nuzhat Parveen, Yan-Liang Lin, Ruey-Hwang Chou, Chung-Ming Sun, Chin Yu

**Affiliations:** ^1^ Chemistry Department, National Tsing Hua University, Hsinchu, Taiwan; ^2^ Department of Applied Chemistry, National Yang Ming Chiao Tung University, Hsinchu, Taiwan; ^3^ Graduate Institute of Biomedical Sciences, China Medical University, Taichung, Taiwan; ^4^ The Ph.D. Program of Biotechnology and Biomedical Industry, China Medical University, Taichung, Taiwan; ^5^ Center for Molecular Medicine, China Medical University Hospital, Taichung, Taiwan; ^6^ Department of Medical Laboratory and Biotechnology, Asia University, Taichung, Taiwan; ^7^ Department of Medicinal and Applied Chemistry, Kaohsiung Medical University, Kaohsiung, Taiwan

**Keywords:** protein-ligand interaction, FGF1, FGFRD2, NMR, WST1 assay, cell proliferation, cytotoxicity

## Abstract

A promising approach in cancer therapy is the inhibition of cell proliferation using small molecules. In this study, we report the synthesis of suramin derivatives and their applications. We used NMR spectroscopy and docking simulations to confirm binding sites and three-dimensional models of the ligand-protein complex. The WST-1 assay was used to assess cell viability and cell proliferation *in vitro* to evaluate the inhibition of protein–protein interactions and to investigate the anti-proliferative activities in a breast cancer cell line. All the suramin derivatives showed anti-proliferative activity by blocking FGF1 binding to its receptor FGFRD2. The dissociation constant was measured by fluorescence spectroscopy. The suramin compound derivatives synthesized herein show potential as novel therapeutic agents for their anti-proliferative activity *via* the inhibition of protein–protein interactions. The cytotoxicity of these suramin derivatives was lower than that of the parent suramin compound, which may be considered a significant advancement in this field. Thus, these novel suramin derivatives may be considered superior anti-metastasis molecules than those of suramin.

## Introduction

One of the most commonly occurring cancers in women is breast cancer, which is the second most common cancer overall. There are around 2 million new cases discovered every year. Female breast cancer ranks as the fifth leading cause of death (627,000 deaths, 6.6%) due to the relatively favorable prognosis (breast cancer statistics). A recent study suggested that suramin, a sulfonated naphthylurea compound, could inhibit cell proliferation in breast cancer cell lines (Wu et al., 2016). Suramin is a member of the phenyl urea class, in which the amino groups have been substituted by a 3-({2-methyl-5-[(4,6,8-trisulfo-1-naphthyl) carbamoyl] phenyl}carbamoyl) (National Center for Biotechnology Information, 2019) phenyl group with potential antineoplastic activity.

Trypanosomiasis and onchocerciasis are common diseases in sub-Saharan Africa and are specifically treated with suramin (Anderson et al., 1976; Cheson, et al., 1987; Barrett et al., 2007; Brun, 2010; Babokhov et al., 2013; Federica et al., 2016; Büscher et al., 2017). This market-approved anti-parasite medication was developed in 1921 and its pharmacological development was stimulated for its antitumor, anti-inflammatory, and antiviral activities (Yahi et al., 1994; Liu. et al., 2012; Basavannacharya and Vasudevan, 2014; Li et al., 2015; Henß et al., 2016; Koval et al., 2016; Albulescu et al., 2017). In the 1980s, suramin was briefly studied as a possible antiviral agent for the treatment of acquired immune deficiency syndrome (AIDS). In addition to inhibiting the function of viral reverse transcriptase, structural changes in the host cell following human immunodeficiency virus (HIV) infection are also blocked by suramin *in vitro* (De Clercq, 1979; Mitsuya et al., 1984; Broder et al., 1985; Clercq, 2021).

Most of the pharmacological and biological properties of suramin are due to the presence of aromatic rings defined by the spatial arrangement of six sulfonic groups. The binding of suramin to proteins usually occurs by ring stacking interactions (i.e., proteins bind to the aromatic amino acids) and is a consequence of its overall anionic charge.

Fibroblast growth factors (FGFs) are systematic mitogenic agents that control a wide variety of cellular functions, including proliferation, differentiation, migration, and survival (Yun et al., 2010; Xie et al., 2020). It has been reported that FGFs play an important role in tissue homeostasis, development, and metabolism (Xie et al., 2020) in a variety of human diseases, such as chronic kidney diseases (CKDs), congenital craniosynostosis, insulin resistance, obesity, dwarfism syndromes, and various tumors.

Based on phylogeny, FGFs are divided into six subfamilies consisting of one endocrine subfamily and five paracrine subfamilies. The five paracrine subfamilies include FGF1, FGF4, FGF7, FGF8, and FGF9 subfamilies, and the endocrine subfamily is represented by the FGF19 subfamily (Ornitz and Itoh, 2001; Beenken and Mohammadi, 2009; Ornitz and Itoh, 2015; Xie et al., 2020). FGFs also play a role in pleiotropism (Wiedemann and Trueb, 2000).

The FGF receptor (FGFR) contains three domains: an extracellular domain, a transmembrane domain, and an intracellular tyrosine kinase domain (Lemmon and Schlessinger, 2010). The extracellular domain consists of three immunoglobulin-like domains (domains D1–D3) (Beenken and Mohammadi, 2009; Goetz and Mohammadi, 2013), an acidic region, heparin cofactors, a partner protein, and a heparin-binding motif for FGFs (Farrell and Breeze, 2018). The transmembrane domain supports receptors and promotes dimerization within the cell membrane. The juxtamembrane region of FGFRs in the cytosol is involved in receptor dimerization, and split kinase domains are involved in the transmission of FGF-related signaling (Goetz and Mohammadi, 2013).

Binding of inactive monomeric FGFRs to FGFs triggers a conformational change in the FGFRs, resulting in the activation and dimerization of tyrosine kinases (Dai et al., 2019; Latko et al., 2019). This occurs by phosphorylating the tyrosine residues in the FGFR cytosolic tail. After phosphorylation, the phosphorylated tyrosine residues are allocated to docking sites for downstream signaling molecules (Furdui et al., 2006; Goetz and Mohammadi, 2013; Ornitz and Itoh, 2015; Farrell and Breeze, 2018) and induces cell proliferation.

Suramin is known to block receptor binding of various growth factors, such as epidermal growth factor (EGF), insulin-like growth factor (IGF-I), platelet-derived growth factor (PDGF), and tumor growth factor-beta (TGF-beta). This results in the inhibition of migration and endothelial cell proliferation (Chamberlain et al., 1995; Schrell et al., 1995; Hosang, 1985; Garg and Corona Benjamin., 2015). The mechanism by which suramin interrupts the activity of growth factors is not completely understood, but it is assumed to bind to FGF directly and not to its complementary receptor (Middaugh et al., 1992; Lozano et al., 1998; Sola et al., 1999; Zamai et al., 2002; Kathir et al., 2006).

Suramin is a polyanion, and the growth factors that it inhibits are primarily heparin-binding proteins. The interaction of suramin with its target proteins is due to its polyanion binding site (La Rocca et al., 1990; Middaugh et al., 1992). Based on the FGF2 crystal structure, suramin inhibits receptor binding in two ways: by changing its conformation and sterically occluding the region of receptor binding (Eriksson et al., 1991; Middaugh et al., 1992; Pagano et al., 2012; Eberle et al., 2019).

Suramin is highly toxic and hypersensitive, and to enhance its chemotherapeutic potential, there is a need to develop drugs with less toxicity. If the polypharmacology of suramin was understood, less toxic and more specific new molecules could be identified for the numerous potential applications of suramin (Wiedemar et al., 2020).

Suramin appears to be a promising antagonist in terms of angiogenic activity. When FGF1 binds to its receptor (FGFRD2), it dimerizes FGFRD2 and brings the cytoplasmic domains of the receptor close to each other. Autophosphorylation between these two cytoplasmic domains then occurs. This triggers a signal transduction cascade and results in cell proliferation. If a compound (such as a suramin derivative) can block the interaction between FGF1 and FGFRD2, it can prevent the dimerization of the receptor FGFRD2, avoid the occurrence of autophosphorylation, and will not allow signal transduction or triggering of cell proliferation, which ultimately results in anti-proliferative activity.

**FIGURE 1:**
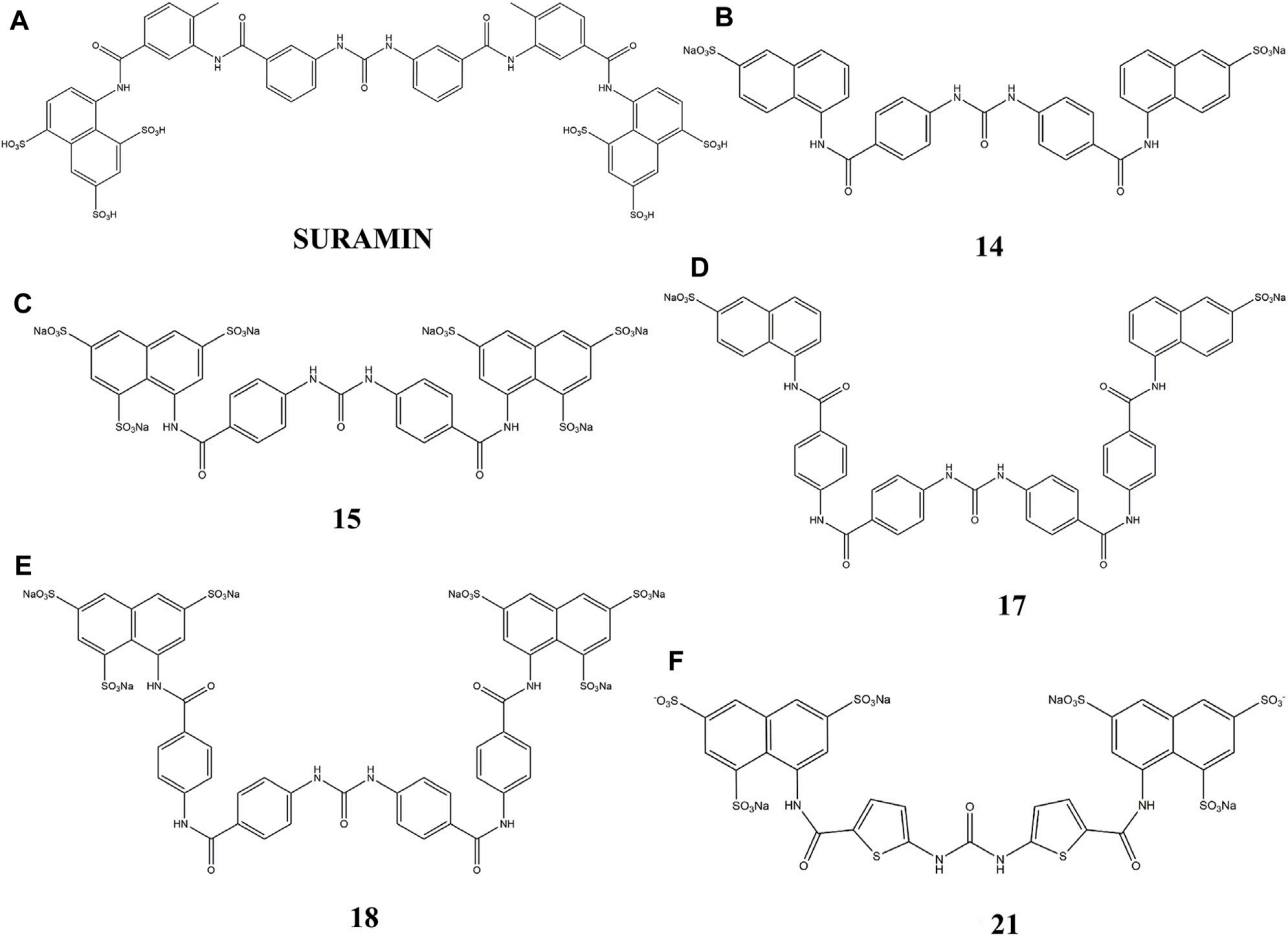
Chemical structure of suramin and its five derivatives (compounds **14, 15, 17, 18** and **21**).

Thus, in this study, we used suramin as the template, to synthesize several suramin derivatives (compounds **14**, **15**, **17**, **18**, and **21**), as shown in Figure 1. These analogs show strong inhibition between FGF1 and its receptor FGFRD2, but have lower cytotoxicity than suramin.

## Materials and Methods

Milli-Q water was used for all buffers and experimental studies. A broad-range protein marker was obtained from Bio-Rad. The MCF7 cell line was purchased from the American Type Culture Collection.

### Synthesis of Compounds

Bioactive compounds derived from suramin were synthesized as shown below. Variations in the aryl sulfonic acid moieties (mono or trisulfonic acids of naphthalene) were incorporated as new building blocks in the target molecules. We introduced novel scaffolds to construct different lengths of suramin-like symmetrical urea structures. Two types of compounds were synthesized: first, the preparation of the suramin-like symmetrical urea compound (**14** and **15**) from its precursors (**6** and **7**); second, replacement of the nitrobenzoyl group by the nitrothiophene moiety in the synthesis of symmetrical urea **21** which contains a heterocyclic building block. To prepare these building blocks, two aminonaphthalenesulfonic acids (**1** and **2**) were allowed to react with carboxylic acid chloride **3a–c** in the aqueous solution of toluene to give nitrobenzamides (**4** and **5**) in high yield.

**SCHEME 1:**
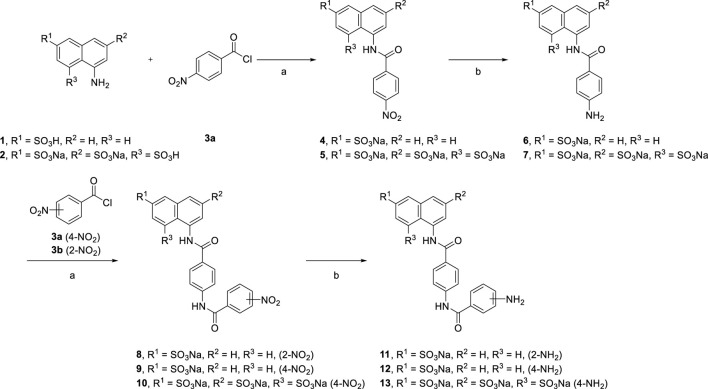
Syntheses of suramin analog precursors **6**, **7**, and **11**, **12, and 13**.

Reduction of the nitro compounds (**4** and **5**) was performed to deliver the corresponding aromatic amines (**6** and **7**) though palladium with charcoal catalysis. Different reduction methods were used in this transformation. We also replaced palladium with zinc powder and used ammonium formate as the hydrogen source. Although, the reaction time was reduced from 16 h to 15 min, many byproducts were formed, which caused difficulty in purifying them after reduction. Moreover, we synthesized suramin symmetrical urea precursors (**11, 12, and 13**) from aromatic amines (**6** and**7**) by amidation and reduction (Scheme 1).

[Reagents/reaction conditions: 1) **1** (200 mg, 0.89 mmol), 4-nitrobenzoyl chloride **3a** (248 mg, 1.34 mmol) in H_2_O (15 ml)/toluene (5 ml), room temperature, 12 h. 2) Pd/C (10%), H_2_ gas, H_2_O (10 ml), room temperature, 16 h]

**SCHEME 2:**
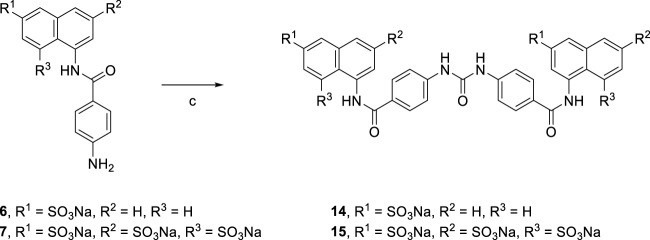
Syntheses of suramin-like derivatives **14** and **15**.

Treatment of aqueous solution of arylamines (**6** and **7**) with a solution of triphosgene in toluene resulted in the synthesis of the target suramin-like derivatives (**14** and **15**, Scheme 2), but these were not soluble in methanol. Therefore, these crude products were purified by washing several times with methanol.

[Reagents/reaction conditions: 3) **6** (200 mg, 0.55 mmol), in H_2_O (15 ml), triphosgene (81.51 mg, 0.27 mmol) in toluene (5 ml), room temperature, 16 h]

**SCHEME 3:**
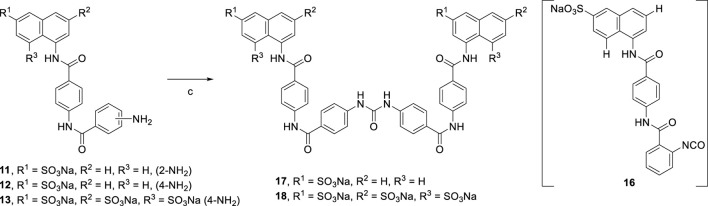
Syntheses of suramin derivatives **17** and **18**.

Suramin precursors **12** and **13** were also successfully used to synthesize suramin derivatives **17** and **18**. However, when suramin precursor **11** was used as a building block to prepare the suramin derivative, an unexpected isocyanate compound **16** was obtained instead of the desired symmetrical urea suramin derivative (Scheme 3).

[Reagents/reaction conditions: 3) **11** (200 mg, 0.41 mmol) in H_2_O (15 ml), triphosgene (61.42 mg, 0.20 mmol) in toluene (5 ml), room temperature, 16 h]

**SCHEME 4:**
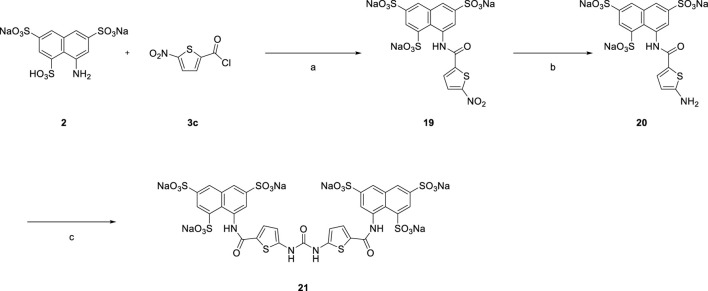
Syntheses of heterocyclic suramin derivative **21**.

In Scheme 4 replacement of the nitrobenzoyl group by the nitrothiophene moiety was achieved for the synthesis of symmetrical urea **21** which contains a heterocyclic building block.

[Reagents/reaction conditions: 1) **2** (200 mg, 0.46 mmol) in H_2_O (15 ml), **3c** (133.66 mg, 0.70 mmol) in toluene (5 ml), room temperature, 12 h. 2) Pd/C (10%), H_2_ gas, H_2_O (10 ml), room temperature, 16 h 3) **20** (200 mg, 0.34 mmol) in H_2_O (15 ml), triphosgene (51.70 mg, 0.17 mmol) in toluene (5 ml), room temperature, 16 h]

**SCHEME 5:**
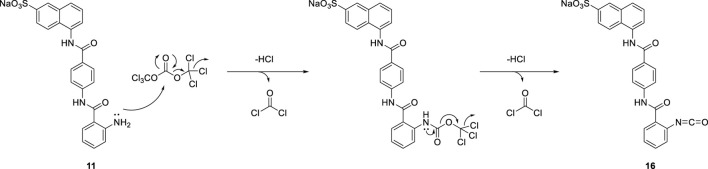
A potential mechanism for the formation of compound **16**.

Due to the steric hindrance on the *ortho*-position of the amino group on precursor **11**, the amidation reaction of activated triphosgene was blocked. The other reaction pathway was *en route.* The potential mechanism for the formation of isocyanate compound **16** is proposed in Scheme 5.

### General acylation procedure and synthesis of nitro derivatives 4, 5, 8, 9, 10, and 19

#### Sodium 5-(4-nitrobenzamido) naphthalene-2-sulfonate (**4**)

In 100 ml water, 5-amino-2-naphthalenesulfonic acid (5 g, 22.42 mmol) was dissolved, and the solution was adjusted to pH 4.0 using 2 M Na_2_CO_3_ solution. Next, in 10 ml toluene, 4-nitrobenzoylchloride (5.80 g, 31.38 mmol) was dissolved and by stirring, it was slowly added to the reaction mixture.

When the coupling reaction was completed, toluene was removed from the reaction mixture. The water phase solution was adjusted to pH 2.0 and then was extracted with diethyl ether (4 × 70 ml). After neutralization of the solution to pH 7.0, the water phase was removed under vacuum. The crude product was purified by recrystallization from methanol in 80% isolated yield (7.06 g). ^1^H NMR (400 MHz, DMSO-*d*_6_) *δ* 10.86 (s, 1H), 8.46–8.27 (m, 4H), 8.25 (s, 1H), 7.90 (dd, *J* = 23.3, 8.1 Hz, 2H), 7.75 (dd, *J* = 8.6, 1.7 Hz, 1H), 7.65–7.52 (m, 2H); LRMS (ESI, *m/z*): 371.1 (M-H)^−^.

#### Sodium 8-(4-nitrobenzamido) naphthalene-1,3,6-trisulfonate (**5**)

Yield: 82%. ^1^H NMR (400 MHz, DMSO-*d*_6_) δ 12.73 (s, 1H), 8.60 (d, *J* = 1.9 Hz, 1H), 8.47 (d, *J* = 1.7 Hz, 1H), 8.40–8.33 (m, 4H), 8.22 (d, *J* = 1.8 Hz, 1H), 8.08 (d, *J* = 1.7 Hz, 1H); LRMS (ESI, *m/z*): 276.1.1 (M + Na-2H)^2−^.

#### Sodium 5-(4-(2-nitrobenzamido) benzamido) naphthalene-2-sulfonate (**8**)

Yield: 67%. ^1^H NMR (400 MHz, DMSO-*d*_6_) δ 11.04 (s, 1H), 10.47 (s, 1H), 8.27 (s, 1H), 8.16 (d, *J* = 8.2 Hz, 1H), 8.10 (d, *J* = 8.3 Hz, 2H), 7.91 (t, *J* = 8.7 Hz, 2H), 7.88–7.78 (m, 4H), 7.75 (t, *J* = 8.7 Hz, 2H), 7.55 (d, *J* = 4.8 Hz, 2H); LRMS (ESI, *m/z*): 492.1 (M + H)^+^.

#### Sodium 5-(4-(4-nitrobenzamido) benzamido) naphthalene-2-sulfonate (**9**)

Yield: 72%. ^1^H NMR (400 MHz, DMSO-*d*_6_) *δ* 10.86 (s, 1H), 10.46 (s, 1H), 8.42–8.34 (m, 2H), 8.29–8.19 (m, 3H), 8.11 (d, *J* = 8.2 Hz, 2H), 7.98 (d, *J* = 8.4 Hz, 2H), 7.91 (dd, *J* = 8.8, 2.9 Hz, 1H), 7.84 (q, *J* = 4.3 Hz, 1H), 7.73 (d, *J* = 8.6 Hz, 1H), 7.54 (d, *J* = 4.3 Hz, 2H); LRMS (ESI, *m/z*): 490.1 (M-H)^−^.

#### Sodium 8-(4-(4-nitrobenzamido) benzamido) naphthalene-1,3,6-trisulfonate (**10**)

Yield: 72%. ^1^H NMR (400 MHz, DMSO-*d*_6_) δ 12.43 (s, 1H), 10.96 (s, 1H), 8.58 (d, *J* = 1.5 Hz, 1H), 8.47 (d, *J* = 1.5 Hz, 1H), 8.34 (d, *J* = 8.7 Hz, 2H), 8.25 (d, *J* = 8.7 Hz, 2H), 8.20–8.13 (m, 3H), 8.02 (s, 1H), 7.95 (d, *J* = 8.6 Hz, 2H); LRMS (ESI, *m/z*): 650.1 (M-H)^-^, 324.8 (M-2H)^2−^.

#### Sodium 8-(5-nitrothiophene-2-carboxamido) naphthalene-1,3,6-trisulfonate (**19**)

Yield: 76%. ^1^H NMR (400 MHz, DMSO-*d*_6_) δ 12.99 (s, 1H), 8.58 (d, *J* = 2.0 Hz, 1H), 8.46 (d, *J* = 1.8 Hz, 1H), 8.21 (s, 2H), 8.16 (d, *J* = 2.0 Hz, 1H), 8.03 (d, *J* = 1.9 Hz, 1H). ); LRMS (ESI, *m/z*): 580.8 (M+2Na-H)^−^.

### General hydrogenation procedure and synthesis of amino derivatives 6, 7, 11, 12, 13, and 20

#### Sodium 5-(4-aminobenzamido) naphthalene-2-sulfonate (**6**)

In 65 ml of water, sodium 5-(4-nitrobenzamido) naphthalene-2-sulfonate **4** (5 g, 12.68 mmol) was dissolved and bubbled by hydrogen and then palladium or carbon (500 mg, 10% of the weight of the nitro compound) was added as a catalyst. After the reduction completion, filtration methods were used for the removal of the palladium catalyst. The water phase was removed. The isolated yield was 82% (3.78 g). ^1^H NMR (400 MHz, DMSO-*d*_*6*_) δ 10.03 (s, 1H), 8.23 (s, 1H), 7.89 (d, J = 8.6 Hz, 1H), 7.80 (d, J = 8.4 Hz, 3H), 7.71 (d, J = 8.4 Hz, 1H), 7.59–7.42 (m, 2H), 6.62 (d, J = 8.4 Hz, 2H), 5.76 (s, 2H); LRMS (ESI, *m/z*): 341.1 (M-H)^−^.

#### Sodium 8-(4-aminobenzamido) naphthalene-1,3,6-trisulfonate (**7**)

Yield: 83%. ^1^H NMR (600 MHz, DMSO-*d*_6_) δ 12.07 (s, 1H), 8.55 (s, 1H), 8.39 (s, 1H), 8.16 (s, 1H), 7.96 (s, 1H), 7.85 (d, *J* = 8.3 Hz, 2H), 6.59 (d, *J* = 8.3 Hz, 2H), 5.65 (s, 2H); LRMS (ESI, *m/z*): 545.0 (M+2Na-H)^−^.

#### Sodium 5-(4-(2-aminobenzamido) benzamido) naphthalene-2-sulfonate (**11**)

Yield: 82%. ^1^H NMR (400 MHz, DMSO-*d*_6_) δ 10.42 (s, 1H), 10.28 (s, 1H), 8.26 (s, 1H), 8.07 (d, *J* = 8.7 Hz, 2H), 7.91 (dd, *J* = 8.7, 2.1 Hz, 3H), 7.83 (t, *J* = 8 Hz, 1H), 7.73 (dd, *J* = 8.5, 1.3 Hz, 1H), 7.67 (d, *J* = 7.1 Hz, 1H), 7.54 (d, *J* = 5.2 Hz, 2H), 7.21 (t, *J* = 8.2 Hz, 1H), 6.76 (d, *J* = 8.1 Hz, 1H), 6.60 (t, *J* = 7.4 Hz, 1H), 6.37 (s, 2H); LRMS (ESI, *m/z*): 462.1 (M + H)^+^.

#### Sodium 5-(4-(4-aminobenzamido) benzamido) naphthalene-2-sulfonate (**12**)

Yield: 70%. ^1^H NMR (400 MHz, DMSO-*d*_6_) *δ* 10.38 (s, 1H), 10.04 (s, 1H), 8.25 (s, 1H), 8.05 (d, J = 8.5 Hz, 2H), 7.93 (dd, J = 15.9, 8.5 Hz, 3H), 7.83 (dd, J = 6.1, 3.4 Hz, 1H), 7.74 (t, J = 9.4 Hz, 3H), 7.59–7.50 (m, 2H), 6.61 (d, J = 8.2 Hz, 2H), 5.80 (s, 2H); LRMS (ESI, *m/z*): 460.1 (M-H)^−^.

#### Sodium 8-(4-(4-aminobenzamido) benzamido) naphthalene-1,3,6-trisulfonate (**13**)

Yield: 75%. ^1^H NMR (600 MHz, DMSO-*d*_6_) δ 12.38 (s, 1H), 10.05 (s, 1H), 8.57 (s, 1H), 8.45 (s, 1H), 8.15 (s, 1H), 8.10 (d, *J* = 8.0 Hz, 2H), 7.98 (s, 1H), 7.91–7.88 (m, 2H), 7.75 (d, *J* = 7.4 Hz, 2H), 6.61 (d, *J* = 7.4 Hz, 2H), 5.81 (s, 2H); LRMS (ESI, *m/z*): 620.1 (M-H)^−^.

#### Sodium 8-(5-aminothiophene-2-carboxamido) naphthalene-1,3,6-trisulfonate (**20**)

Yield: 80%. ^1^H NMR (400 MHz, DMSO-*d*_6_) δ 12.14 (s, 1H), 8.60 (s, 1H), 8.55 (s, 1H), 8.18 (s, 1H), 7.96 (s, 1H), 7.74 (d, *J* = 3.6 Hz, 1H), 6.36 (s, 2H), 5.93 (d, *J* = 3.6 Hz, 1H).

### Synthesis of urea derivatives 14, 15, 17, 18, and 21 and isocyanate compound 16.

#### Sodium 5,5'-((4,4'-(carbonylbis (azanediyl)) bis (benzoyl)) bis (azanediyl)) bis (naphthalene-2-sulfonate) (**14**)

In 10 ml water, sodium 5-(4-aminobenzamido) naphthalene-2-sulfonate **6** (1 g, 2.74 mmol) was dissolved, and the solution was adjusted to pH 4.0 using 2 M Na_2_CO_3_ solution. Triphosgene (0.4 g, 1.37 mmol), dissolved in 5 ml toluene, was added to the reaction mixture slowly by stirring.

Toluene was removed after the completion of the reaction from the water layer, and then the water phase was neutralized using 2 M Na_2_CO_3_. Under vacuum, the water phase was evaporated. The crude product was purified by washing with methanol (2 × 5 ml) to obtain a pure compound in 65% (1.34 g). ^1^H NMR (400 MHz, DMSO-*d*_6_) δ 10.41 (s, 2H), 10.18 (s, 2H), 8.28 (s, 2H), 8.06 (d, *J* = 7.8 Hz, 4H), 7.93 (d, *J* = 8.3 Hz, 2H), 7.86–7.81 (m, 2H), 7.75 (d, *J* = 8.3 Hz, 2H), 7.68 (d, *J* = 7.8 Hz, 4H), 7.58–7.51 (m, 4H); ^13^C NMR (400 MHz, DMSO-*d*_6_) *δ* 166.1, 153.0, 145.6, 143.6, 135.3, 134.0, 129.4, 129.3, 128.1, 127.6, 126.5, 126.3, 125.2, 124.4, 120.3, and 117.4; LRMS (ESI, *m/z*): 755.0 (M + H)^+^; HRMS (ESI) calcd for C_35_H_24_N_4_NaO_9_S_2_ *m/z*: 731.0882; found 731.0885 (M + Na-H)^−^; C_35_H_24_N_4_O_9_S_2_ *m/z*: 708.0984; found 708.0968 (M-2H)^2−^.

#### Sodium 8,8'-((4,4'-(carbonylbis (azanediyl)) bis (benzoyl)) bis (azanediyl))bis (naphthalene-1,3,6-trisulfonate) (**15**)

Yield: 73%. ^1^H NMR (400 MHz, DMSO-*d*_6_) δ 12.32 (s, 2H), 9.80 (s, 2H), 8.56 (d, *J* = 1.9 Hz, 2H), 8.42 (d, *J* = 1.8 Hz, 2H), 8.15 (d, *J* = 1.8 Hz, 2H), 8.09 (d, *J* = 8.8 Hz, 4H), 7.98 (d, *J* = 1.7 Hz, 2H), 7.59 (d, *J* = 8.8 Hz, 4H); ^13^C NMR (400 MHz, DMSO-*d*_6_) *δ* 165.3, 153.2, 145.1, 143.5, 143.4, 142.0, 134.9, 133.9, 129.4, 128.7, 128.5, 126.5, 122.9, 122.5, and 117.3; LRMS (ESI, *m/z*): 514.0 (M-2H)^2−^.

#### Sodium 5-(4-(2-isocyanatobenzamido) benzamido) naphthalene-2-sulfonate (**16**)

Yield: 71%. ^1^H NMR (400 MHz, DMSO-*d*_6_) δ 11.61 (s, 1H), 10.64 (s, 1H), 8.32 (s, 1H), 8.15 (d, *J* = 7.0 Hz, 2H), 7.95 (dd, *J* = 14.2, 8.1 Hz, 2H), 7.86 (d, *J* = 8 Hz, 2H), 7.73 (dd, *J* = 18.1, 7.7 Hz, 2H), 7.59–7.53 (m, 3H), 7.30–7.19 (m, 2H); ^13^C NMR (400 MHz, DMSO-*d*_6_) *δ* 166.3, 162.5, 150.4, 145.9, 140.3, 139.1, 135.7, 134.9, 134.7, 134.0, 129.7, 129.1, 128.7, 128.2, 128.0, 126.6, 126.5, 125.1, 124.5, 123.0, 120.0, 115.7, and 114.7; LRMS (ESI, *m/z*): 486.0 (M-H)^−^; HRMS (ESI) calcd for C_49_H_37_N_6_Na1O_11_S_2_ *m/z*: 972.1859; found 972.1459 (M-H)^−^.

#### Sodium 5,5'-((4,4'-((4,4'-(carbonylbis (azanediyl)) bis (benzoyl)) bis (azanediyl)) bis (benzoyl)) bis(azanediyl)) bis (naphthalene-2-sulfonate) (**17**)

Yield: 73%. ^1^H NMR (400 MHz, DMSO-*d*_6_): *δ* 10.62 (s, 2H), 10.47 (s, 2H), 10.41 (s, 2H), 8.34 (s, 2H), 8.19 (s, 2H), 8.10 (d, *J* = 8.5 Hz, 4H), 8.05–7.97 (m, 6H), 7.95 (d, *J* = 8.9 Hz, 2H), 7.89 (d, *J* = 8.0 Hz, 2H), 7.76–7.71 (m, 2H), 7.67 (d, *J* = 8.3 Hz, 4H), 7.61 (d, *J* = 6.8 Hz, 2H), 7.55 (t, *J* = 7.7 Hz, 2H); ^13^C NMR (400 MHz, DMSO-*d*_6_) *δ* 166.3, 165.8, 152.8, 145.4, 145.1, 143.5, 142.9, 134.2, 133.3, 129.5, 129.4, 129.2, 129.0, 127.7, 127.3, 126.6, 125.1, 124.9, 124.0, 123.7, 120.0, and 117.5; LRMS (ESI, *m/z*): 947.2 (M-H)^-^, 473.3 (M-2H)^2-^; HRMS (ESI) calcd for C_49_H_36_N_6_O_11_S_2_ *m/z*: 947.1811; found 947.1885 (M-H)^−^.

#### Sodium 8,8'-((4,4'-((4,4'-(carbonylbis (azanediyl)) bis (benzoyl)) bis (azanediyl)) bis (benzoyl)) bis(azanediyl)) bis (naphthalene-1,3,6-trisulfonate) (**18**)

Yield: 70%. ^1^H NMR (600 MHz, DMSO-*d*_6_) δ 12.38 (s, 2H), 10.42 (s, 2H), 9.92 (s, 2H), 8.58 (s, 2H), 8.45 (s, 2H), 8.19 (s, 2H), 8.13 (d, *J* = 7.1 Hz, 4H), 8.02 (s, 2H), 8.01–7.96 (m, 4H), 7.95–7.88 (m, 4H), 7.64 (s, 4H); LRMS (ESI, *m/z*): 677.3 (M+4Na-2H)^2−^

#### Sodium 8,8'-((5,5'-(carbonylbis (azanediyl)) bis (thiophene-5,2-diyl-2-carbonyl)) bis (azanediyl)) bis (naphthalene-1,3,6-trisulfonate) (**21**)

Yield: 70%. ^1^H NMR (400 MHz, DMSO-*d*_6_) δ 12.38 (s, 4H), 8.57 (d, *J* = 6.9 Hz, 4H), 8.14 (s, 2H), 7.95 (d, *J* = 6.9 Hz, 4H), 6.60 (s, 2H); LRMS (ESI, *m/z*): 519.9 (M-2H)^2−^.

The NMR results for all the compounds are shown in the supporting file Supplementary Figure S1.

### Exposure Stability Studies

The stability of the compounds was verified in both aqueous and DMSO solutions. In aqueous medium, the stability of the suramin derivatives **15** and **18** were considered to verify the stability. A control experiment consisting of a DMSO-*d*_*6*_ (0.017 mol/L) solution of **15** and a D_2_O solution of **15** (0.017 mol/L) was maintained on the laboratory bench. All experiments were performed in NMR tubes containing 0.017 mol/L solutions of compounds **15** and **18** in DMSO-*d*_*6*_ and D_2_O, respectively. NMR spectra were recorded over 11 days (initially daily for 1 week). As shown in the ^1^H NMR spectra, the suramin derivatives **15** and **18** were remarkably stable under these solutions (Supplementary Figure S2).

#### Exposure Stability Study in DMSO

10 mg of the newly synthesized compound was diluted in 500 µL DMSO-*d*_*6*_ under investigation in a 5-mm NMR tube. The procedure was maintained as it is for 11 days in the solution. The ^1^H NMR spectrum was recorded daily for 4 days up to 11 days.

#### Exposure Stability Study in Water

10 mg of the novel compound was diluted in 500 µL D_2_O under investigation in a 5-mm NMR tube. The procedure was maintained as it is for 11 days in the solution. The ^1^H NMR spectrum was recorded daily for 4 days up to 11 days.

### Characterization of the Novel Compounds

The physical properties of new compounds were determined to define accurate mass data using high-resolution mass spectrometry (HRMS) to support the molecular formula assignment.

#### Sodium 5-(4-nitrobenzamido) naphthalene-2-sulfonate (**4**)

In 100 ml water, 5-amino-2-naphthalenesulfonic acid (5 g, 22.42 mmol) was dissolved, and the solution was adjusted to pH 4 by adding 2 M Na_2_CO_3_ solution. Next, 4-nitrobenzoylchloride **2** (5.80 g, 31.38 mmol) was dissolved in 10 ml toluene and was added slowly by stirring to the reaction mixture.

When the coupling reaction was completed, toluene was removed from the reaction mixture. The pH of the water phase solution was adjusted to 2.0, and diethyl ether was used for the extraction of the water phase (4 × 70 ml). Under vacuum, the water phase was removed after the neutralization of the solution to pH 7.0. The crude product was purified by recrystallization from methanol in 80% isolated yield (7.06 g). Brown solid; ^1^H NMR (400 MHz, DMSO-*d*_6_) *δ* 10.86 (s, 1H), 8.46–8.27 (m, 4H), 8.25 (s, 1H), 7.90 (dd, *J* = 23.3, 8.1 Hz, 2H), 7.75 (dd, *J* = 8.6, 1.7 Hz, 1H), 7.65–7.52 (m, 2H); LRMS (ESI, *m/z*): 371.1 (M-H)^−^.

#### Sodium 8-(4-nitrobenzamido) naphthalene-1, 3, 6-trisulfonate (**5**)

Yellow solid; Yield: 82%. ^1^H NMR (400 MHz, DMSO-*d*_6_) δ 12.73 (s, 1H), 8.60 (d, *J* = 1.9 Hz, 1H), 8.47 (d, *J* = 1.7 Hz, 1H), 8.40–8.33 (m, 4H), 8.22 (d, *J* = 1.8 Hz, 1H), 8.08 (d, *J* = 1.7 Hz, 1H); LRMS (ESI, *m/z*): 276.1.1 (M + Na-2H)^2−^.

#### Sodium 5-(4-(2-nitrobenzamido) benzamido) naphthalene-2-sulfonate (**8**)

Brown solid; Yield: 67%. ^1^H NMR (400 MHz, DMSO-*d*_6_) δ 11.04 (s, 1H), 10.47 (s, 1H), 8.27 (s, 1H), 8.16 (d, *J* = 8.2 Hz, 1H), 8.10 (d, *J* = 8.3 Hz, 2H), 7.91 (t, *J* = 8.7 Hz, 2H), 7.88–7.78 (m, 4H), 7.75 (t, *J* = 8.7 Hz, 2H), 7.55 (d, *J* = 4.8 Hz, 2H); LRMS (ESI, *m/z*): 492.1 (M + H)^+^.

#### Sodium 5-(4-(4-nitrobenzamido) benzamido) naphthalene-2-sulfonate (**9**)

Brown solid; Yield: 72%. ^1^H NMR (400 MHz, DMSO-*d*_6_) *δ* 10.86 (s, 1H), 10.46 (s, 1H), 8.42–8.34 (m, 2H), 8.29–8.19 (m, 3H), 8.11 (d, *J* = 8.2 Hz, 2H), 7.98 (d, *J* = 8.4 Hz, 2H), 7.91 (dd, *J* = 8.8, 2.9 Hz, 1H), 7.84 (q, *J* = 4.3 Hz, 1H), 7.73 (d, *J* = 8.6 Hz, 1H), 7.54 (d, *J* = 4.3 Hz, 2H); LRMS (ESI, *m/z*): 490.1 (M-H)^−^.

#### Sodium 8-(4-(4-nitrobenzamido) benzamido) naphthalene-1,3,6-trisulfonate (**10**)

White solid; Yield: 72%. ^1^H NMR (400 MHz, DMSO-*d*_6_) δ 12.43 (s, 1H), 10.96 (s, 1H), 8.58 (d, *J* = 1.5 Hz, 1H), 8.47 (d, *J* = 1.5 Hz, 1H), 8.34 (d, *J* = 8.7 Hz, 2H), 8.25 (d, *J* = 8.7 Hz, 2H), 8.20–8.13 (m, 3H), 8.02 (s, 1H), 7.95 (d, *J* = 8.6 Hz, 2H); LRMS (ESI, *m/z*): 650.1 (M-H)^-^, 324.8 (M-2H)^2−^.

#### Sodium 8-(5-nitrothiophene-2-carboxamido) naphthalene-1,3,6-trisulfonate (**19**)

Yellow solid; Yield: 76%. ^1^H NMR (400 MHz, DMSO-*d*_6_) δ 12.99 (s, 1H), 8.58 (d, *J* = 2.0 Hz, 1H), 8.46 (d, *J* = 1.8 Hz, 1H), 8.21 (s, 2H), 8.16 (d, *J* = 2.0 Hz, 1H), 8.03 (d, *J* = 1.9 Hz, 1H). ); LRMS (ESI, *m/z*): 580.8 (M+2Na-H)^−^.

#### Sodium 5-(4-aminobenzamido) naphthalene-2-sulfonate (**6**)

In 65 ml of water, sodium 5-(4-nitrobenzamido) naphthalene-2-sulfonate **4** (5 g, 12.68 mmol) was dissolved and bubbled by hydrogen, and then palladium or carbon was added as catalysts (500 mg, 10% of the weight of the nitro compound). When the reduction was completed, the palladium catalyst was removed by the filtration method. The water phase was removed. The isolated yield was 82% (3.78 g). Brown solid; ^1^H NMR (400 MHz, DMSO-*d*_*6*_) δ 10.03 (s, 1H), 8.23 (s, 1H), 7.89 (d, J = 8.6 Hz, 1H), 7.80 (d, J = 8.4 Hz, 3H), 7.71 (d, J = 8.4 Hz, 1H), 7.59–7.42 (m, 2H), 6.62 (d, J = 8.4 Hz, 2H), 5.76 (s, 2H); LRMS (ESI, *m/z*): 341.1 (M-H)^−^.

Sodium 8-(4-aminobenzamido) naphthalene-1,3,6-trisulfonate (**7**)

Yellow solid; Yield: 83%. ^1^H NMR (600 MHz, DMSO-*d*_6_) δ 12.07 (s, 1H), 8.55 (s, 1H), 8.39 (s, 1H), 8.16 (s, 1H), 7.96 (s, 1H), 7.85 (d, *J* = 8.3 Hz, 2H), 6.59 (d, *J* = 8.3 Hz, 2H), 5.65 (s, 2H); LRMS (ESI, *m/z*): 545.0 (M+2Na-H)^−^.

#### Sodium 5-(4-(2-aminobenzamido) benzamido) naphthalene-2-sulfonate (**11**)

Brown solid; Yield: 82%. ^1^H NMR (400 MHz, DMSO-*d*_6_) δ 10.42 (s, 1H), 10.28 (s, 1H), 8.26 (s, 1H), 8.07 (d, *J* = 8.7 Hz, 2H), 7.91 (dd, *J* = 8.7, 2.1 Hz, 3H), 7.83 (t, *J* = 8 Hz, 1H), 7.73 (dd, *J* = 8.5, 1.3 Hz, 1H), 7.67 (d, *J* = 7.1 Hz, 1H), 7.54 (d, *J* = 5.2 Hz, 2H), 7.21 (t, *J* = 8.2 Hz, 1H), 6.76 (d, *J* = 8.1 Hz, 1H), 6.60 (t, *J* = 7.4 Hz, 1H), 6.37 (s, 2H); LRMS (ESI, *m/z*): 462.1 (M + H)^+^.

#### Sodium 5-(4-(4-aminobenzamido) benzamido) naphthalene-2-sulfonate (**12**)

Compound **12** was synthesized from sodium 5-(4-(4-nitrobenzamido) benzamido) naphthalene-2-sulfonate **9** according to the method described for the synthesis of sodium 5-(4-aminobenzamido ) naphthalene-2-sulfonate **6**. Brown solid; Yield: 70%. ^1^H NMR (400 MHz, DMSO-*d*_6_) *δ* 10.38 (s, 1H), 10.04 (s, 1H), 8.25 (s, 1H), 8.05 (d, J = 8.5 Hz, 2H), 7.93 (dd, J = 15.9, 8.5 Hz, 3H), 7.83 (dd, J = 6.1, 3.4 Hz, 1H), 7.74 (t, J = 9.4 Hz, 3H), 7.59–7.50 (m, 2H), 6.61 (d, J = 8.2 Hz, 2H), 5.80 (s, 2H); LRMS (ESI, *m/z*): 460.1 (M-H)^−^.

#### Sodium 8-(4-(4-aminobenzamido) benzamido) naphthalene-1,3,6-trisulfonate (**13**)

White solid; Yield: 75%. ^1^H NMR (600 MHz, DMSO-*d*_6_) δ 12.38 (s, 1H), 10.05 (s, 1H), 8.57 (s, 1H), 8.45 (s, 1H), 8.15 (s, 1H), 8.10 (d, *J* = 8.0 Hz, 2H), 7.98 (s, 1H), 7.91–7.88 (m, 2H), 7.75 (d, *J* = 7.4 Hz, 2H), 6.61 (d, *J* = 7.4 Hz, 2H), 5.81 (s, 2H); LRMS (ESI, *m/z*): 620.1 (M-H)^−^.

#### Sodium 8-(5-aminothiophene-2-carboxamido) naphthalene-1,3,6-trisulfonate (**20**)

Yellow solid; Yield: 80%. ^1^H NMR (400 MHz, DMSO-*d*_6_) δ 12.14 (s, 1H), 8.60 (s, 1H), 8.55 (s, 1H), 8.18 (s, 1H), 7.96 (s, 1H), 7.74 (d, *J* = 3.6 Hz, 1H), 6.36 (s, 2H), 5.93 (d, *J* = 3.6 Hz, 1H).

#### Sodium 5,5'-((4,4'-(carbonylbis (azanediyl)) bis (benzoyl)) bis (azanediyl)) bis (naphthalene-2-sulfonate) (**14**)

In 10 ml of water, sodium 5-(4-aminobenzamido) naphthalene-2-sulfonate **6** (1 g, 2.74 mmol) was dissolved, and the solution was adjusted to pH 4 by using solution of 2 M Na_2_CO_3_. 5 ml toluene in which triphosgene (0.4 g, 1.37 mmol) was dissolved was added slowly by stirring in the reaction mixture.

Toluene was removed from the water layer after the reaction was completed, and by using the 2 M Na_2_CO_3,_ the water phase was neutralized. Under vacuum, evaporation of the water phase was performed, and the purification of the crude product was carried out by washing with methanol (2 × 5 ml) to obtain a pure compound in 65% (1.34 g). Brown solid; ^1^H NMR (400 MHz, DMSO-*d*_6_) δ 10.41 (s, 2H), 10.18 (s, 2H), 8.28 (s, 2H), 8.06 (d, *J* = 7.8 Hz, 4H), 7.93 (d, *J* = 8.3 Hz, 2H), 7.86–7.81 (m, 2H), 7.75 (d, *J* = 8.3 Hz, 2H), 7.68 (d, *J* = 7.8 Hz, 4H), 7.58–7.51 (m, 4H); ^13^C NMR (400 MHz, DMSO-*d*_6_) *δ* 166.1, 153.0, 145.6, 143.6, 135.3, 134.0, 129.4, 129.3, 128.1, 127.6, 126.5, 126.3, 125.2, 124.4, 120.3, and 117.4; LRMS (ESI, *m/z*): 755.0 (M + H)^+^; HRMS (ESI) calcd for C_35_H_24_N_4_NaO_9_S_2_ *m/z*: 731.0882; found 731.0885 (M + Na-H)^-^; C_35_H_24_N_4_O_9_S_2_ *m/z*: 708.0984; found 708.0968 (M-2H)^2−^.

#### Sodium 8,8'-((4,4'-(carbonylbis (azanediyl)) bis (benzoyl)) bis (azanediyl)) bis (naphthalene-1,3,6-trisulfonate) (**15**)

White solid; Yield: 73%. ^1^H NMR (400 MHz, DMSO-*d*_6_) δ 12.32 (s, 2H), 9.80 (s, 2H), 8.56 (d, *J* = 1.9 Hz, 2H), 8.42 (d, *J* = 1.8 Hz, 2H), 8.15 (d, *J* = 1.8 Hz, 2H), 8.09 (d, *J* = 8.8 Hz, 4H), 7.98 (d, *J* = 1.7 Hz, 2H), 7.59 (d, *J* = 8.8 Hz, 4H); ^13^C NMR (400 MHz, DMSO-*d*_6_) *δ* 165.3, 153.2, 145.1, 143.5, 143.4, 142.0, 134.9, 133.9, 129.4, 128.7, 128.5, 126.5, 122.9, 122.5, and 117.3; LRMS (ESI, *m/z*): 514.0 (M-2H)^2−^.

#### Sodium 5-(4-(2-isocyanatobenzamido) benzamido) naphthalene-2-sulfonate (**16**)

White solid; Yield: 71%. ^1^H NMR (400 MHz, DMSO-*d*_6_) δ 11.61 (s, 1H), 10.64 (s, 1H), 8.32 (s, 1H), 8.15 (d, *J* = 7.0 Hz, 2H), 7.95 (dd, *J* = 14.2, 8.1 Hz, 2H), 7.86 (d, *J* = 8 Hz, 2H), 7.73 (dd, *J* = 18.1, 7.7 Hz, 2H), 7.59–7.53 (m, 3H), 7.30–7.19 (m, 2H); ^13^C NMR (400 MHz, DMSO-*d*_6_) *δ* 166.3, 162.5, 150.4, 145.9, 140.3, 139.1, 135.7, 134.9, 134.7, 134.0, 129.7, 129.1, 128.7, 128.2, 128.0, 126.6, 126.5, 125.1, 124.5, 123.0, 120.0, 115.7, and 114.7; LRMS (ESI, *m/z*): 486.0 (M-H)^-^; HRMS (ESI) calcd for C_49_H_37_N_6_Na1O_11_S_2_ *m/z*: 972.1859; found 972.1459 (M-H)^−^.

#### Sodium 5,5'-((4,4'-((4,4'-(carbonylbis (azanediyl)) bis (benzoyl)) bis (azanediyl)) bis (benzoyl)) bis (azanediyl)) bis (naphthalene-2-sulfonate) (**17**)

Brown solid; Yield: 73%. ^1^H NMR (400 MHz, DMSO-*d*_6_) *δ* 10.62 (s, 2H), 10.47 (s, 2H), 10.41 (s, 2H), 8.34 (s, 2H), 8.19 (s, 2H), 8.10 (d, *J* = 8.5 Hz, 4H), 8.05–7.97 (m, 6H), 7.95 (d, *J* = 8.9 Hz, 2H), 7.89 (d, *J* = 8.0 Hz, 2H), 7.76–7.71 (m, 2H), 7.67 (d, *J* = 8.3 Hz, 4H), 7.61 (d, *J* = 6.8 Hz, 2H), 7.55 (t, *J* = 7.7 Hz, 2H); ^13^C NMR (400 MHz, DMSO-*d*_6_) *δ* 166.3, 165.8, 152.8, 145.4, 145.1, 143.5, 142.9, 134.2, 133.3, 129.5, 129.4, 129.2, 129.0, 127.7, 127.3, 126.6, 125.1, 124.9, 124.0, 123.7, 120.0, 117.5; LRMS (ESI, *m/z*): 947.2 (M-H)^-^, 473.3 (M-2H)^2-^; HRMS (ESI) calcd for C_49_H_36_N_6_O_11_S_2_ *m/z*: 947.1811; found 947.1885 (M-H)^−^.

#### Sodium 8,8'-((4,4'-((4,4'-(carbonylbis (azanediyl)) bis (benzoyl)) bis (azanediyl)) bis (benzoyl)) bis (azanediyl)) bis (naphthalene-1,3,6-trisulfonate) (**18**)

White solid; Yield: 70%. ^1^H NMR (600 MHz, DMSO-*d*_6_) δ 12.38 (s, 2H), 10.42 (s, 2H), 9.92 (s, 2H), 8.58 (s, 2H), 8.45 (s, 2H), 8.19 (s, 2H), 8.13 (d, *J* = 7.1 Hz, 4H), 8.02 (s, 2H), 8.01–7.96 (m, 4H), 7.95–7.88 (m, 4H), 7.64 (s, 4H); LRMS (ESI, *m/z*): 677.3 (M+4Na-2H)^2−^

#### Sodium 8,8'-((5,5'-(carbonylbis (azanediyl)) bis (thiophene-5,2-diyl-2-carbonyl)) bis (azanediyl)) bis (naphthalene-1,3,6-trisulfonate) (**21**)

Yellow solid; Yield: 70%. ^1^H NMR (400 MHz, DMSO-*d*_6_) δ 12.38 (s, 4H), 8.57 (d, *J* = 6.9 Hz, 4H), 8.14 (s, 2H), 7.95 (d, *J* = 6.9 Hz, 4H), 6.60 (s, 2H); LRMS (ESI, *m/z*): 519.9 (M-2H)^2−^.

### Purification of FGF1 and FGFR Proteins

The expression and the purification of FGF1 and FGFRD2 together with the respective experimental procedures are reported in the literature (Wu et al., 2016). The purification of the protein was performed using HPLC, and the protein band was confirmed by SDS-PAGE followed by electrospray ionization (ESI)-mass spectroscopy, and the results are shown in Supplementary Figures S3–S5.

### Sample Preparation for the NMR Experiments

A minimal (M9) medium with ^15^NH_4_Cl was prepared to obtain ^15^N isotope labeling. The same concentration of vitamins was added to the M9 medium for the maximum expression yield. Thiamine was added in the medium as the host strain BL21 (DE3) and pLyss does not have a sufficient amount of vitamin B1.

### NMR Experiments

The NMR experiment was performed on a Varian 700-MHz equipped with a cryoprobe spectrometer. A concentration of approximately 0.5 mM was used for the ^1^H-^15^N heteronuclear single quantum coherence (HSQC) experiments. The buffer used for the sample preparation contained 10% D_2_O, 50 mM AMS, 20 mM phosphate buffer, and 50 mM NaCl at pH 6.5.

### Docking Experiments

Docking of FGF1 and the synthesized compounds was performed using the software HADDOCK (Chang et al., 2016; Khan et al., 2018). The PDB files for the HADDOCK experiment were all obtained from the Protein Data Bank with the following ID’s: FGF1: 1BAR and FGF1-D2 complex: 3CU1. The PDB files for the suramin derivatives were generated using PyMOL (DeLano, 2020).

### Fluorescence Spectroscopy

The binding constant K_d_ was calculated for FGF1 and the five derivatives in a buffer containing 10% D_2_O, 50 mM AMS, 20 mM phosphate buffer, and 50 mM NaCl at pH 6.5 using an F-2500 Hitachi fluorescence spectrophotometer.

### Water-Soluble Tetrazolium Salt Assay for Cell Proliferation and Cytotoxicity

The water-soluble tetrazolium salt (WST-1) assay was performed to assess the effects of the compounds on cell proliferation and cytotoxicity. DMEM/F12 containing 10% FBS was used for the cell culture, and incubation was performed in 5% CO_2_ till the logarithmic growth phase was achieved. On the day before the experiment, the cells were trypsinized and seeded in a 96-well plate at a density of 5000 cells/well. For the FGF1-stimulated cell proliferation assay, the cells were then incubated for 24 h in a serum-free medium containing 0.1% bovine serum albumin (BSA). Serum-starved cells with or without a specified concentration of suramin, **14**, **15**, **17**, **18**, **21**, and FGFRD2 were stimulated with 100 nM of FGF1 again for 48 h. For the purpose of the cytotoxicity assay, the cells were treated with an increased concentration of suramin and compounds **14**, **17**, **15**, **18**, and **21** (Figure 6) for 48 h. Also, 1/10 of the volume of WST-1 cell reagent was inserted in every well and maintained at 37°C for another 4 h before harvesting. There was gentle agitation of the medium in the cell culture plate for 10 min using a shaker. A Synergy 2 microplate reader (BioTek Instruments, Inc., VT, United States) was used to measure the absorbance at 450 nm. The relative absorbance was compared with the control treatment to determine the relative cell number.

## Results and Discussion

Suramin is used successfully for onchocerciases treatment and parasitic infections. In particular, suramin has been tested for NSCLC’s, prostate cancer, and brain tumors. Most studies have been carried out based on suramin’s capability to behave as an antagonist against growth factors that are frequently overexpressed by tumors. Suramin and its five derivatives indicated in Figure 1 show strong antitumor activity and a capacity to disrupt the interaction of FGF1 from its receptors of the D2 and D3 domains of the FGFR (Sola et al., 1999; Zamai et al., 2002; Manetti et al., 2005).

### Characterization of Suramin Analog Binding Sites on FGF1

The NMR HSQC experiments perform an essential role in the determination of the backbone conformation of a protein. Chemical shift perturbation and decreases in peak intensity occur as shown in the HSQC spectra upon the addition of suramin derivatives to the protein. These changes provide significant data about protein-drug binding and provide insight about the binding site between the protein and drugs (Yu et al., 2003). All the residues that are clearly absent in the tested compounds (**14**, **15**, **17**, **18**, and **21**) represent the backbone amide of FGF1 (Ogura et al., 1999; Chi et al., 2002).

**FIGURE 2:**
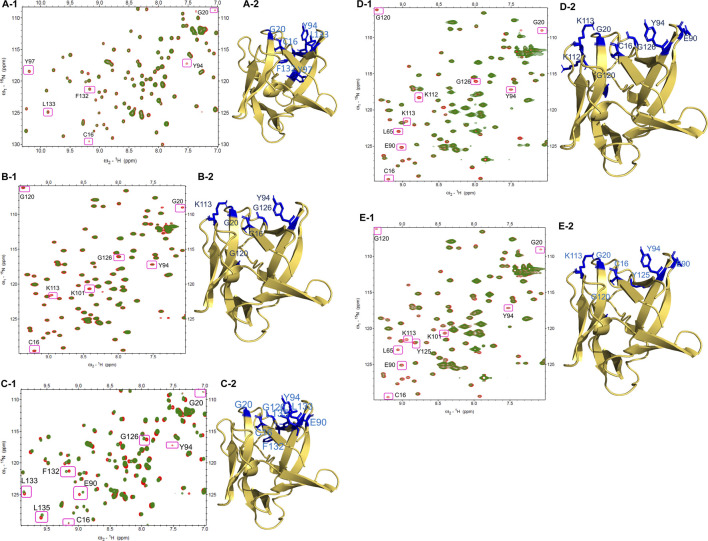
HSQC Spectra of free FGF1 (red) overlapped with different compounds to 1: 1 ratio (green): **(A-1)** HSQC spectrum showing peak intensity decrease and some peak disappearances upon addition of compound **14**. **(A-2)** Residues that shows peak intensity decrease or disappeared are highlighted in blue (side-chain) in the illustration of cartoon form of FGF1. **(B-1)** HSQC spectrum showing peak intensity decrease and some peak disappearances by compound **15**. **(B-2)** Residues that showing decreased peak intensity or disappeared are highlighted in blue (side-chain) in the illustration of cartoon form of FGF1. **(C-1)** HSQC spectrum showing perturbation and peak disappearance by compound **17** (green). **(C-2)** Residues that were perturbed or disappeared are highlighted in blue (side-chain) in the illustration of cartoon form of FGF1. **(D-1)** HSQC spectrum displaying peak intensity decrease and peak disappearance by compound **18**. **(D-2)** Residues that decrease in their peak intensity or disappeared are highlighted in blue (side-chain) in the illustration of cartoon form of FGF1. **(E-1)** HSQC spectrum displaying peak intensity decrease and peak disappearance by compound **21**. **(E-2)** Residues showing peak intensity decrease or disappeared are highlighted in blue (side-chain) in the illustration of cartoon form of FGF1.

The changes in the residues of the ^1^H-^15^N HSQC spectra of FGF1 indicate the putative binding sites of compounds on the protein acquired mostly at 1:1 molar ratio of protein to drug. At a 1:1 ratio of FGF1 to compound **14**, three residues in FGF1 display peak disappearance (C16, G20, and Y94), and another three cross-peaks show a decrease in intensity (Y97, L132, and L133), as shown in Figure 2A-1. The side chains of these residues (stick form in blue) are shown in the structure of FGF1 in Figure 2A-2.

For the titration of FGF1 and compound **15** at a ratio of 1:1, four residues showed a decrease in peak intensity (C16, Y94, G120, and G126), and three residues showed peak disappearance (G20, K101, and K113) (Figure 2B-1). Representations of the side chains of these residues (stick form in blue) are shown in the structure of FGF1 in Figure 2B-2. At a 1:1 ratio of FGF1 to compound **17**, one peak disappeared (Y94) after titration, and seven peaks showed chemical shift perturbation (C16, G20, E90, G126, F132, L133, and L135) (Figure 2C-1). The side chains of the perturbed residues (stick form painted in blue) are illustrated in the structure of FGF1 in Figure 2C-2. The titration of FGF1 and compound **18** at a ratio of 1:1 displayed a decrease in peak intensity for six residues (C16, L65, E90, Y94, K112, and G120), and three residues completely disappeared (G20, K113, and G126) (Figure 2D-1). A representation of the side chains of the altered residues (stick form in blue) is shown in Figure 2D-2. At a 1:0.5 ratio of FGF1 to compound **21**, the titration resulted in a decrease in peak intensity for five residues (L65, E90, Y94, K101 and Y125), and four residues showed peak disappearance (C16, G20, K113, and G120) (Figure 2E-1). The side chains of the residues that were altered or disappeared (stick form in blue) are shown in the structure of FGF1 in Figure 2E-2.

**FIGURE 3:**
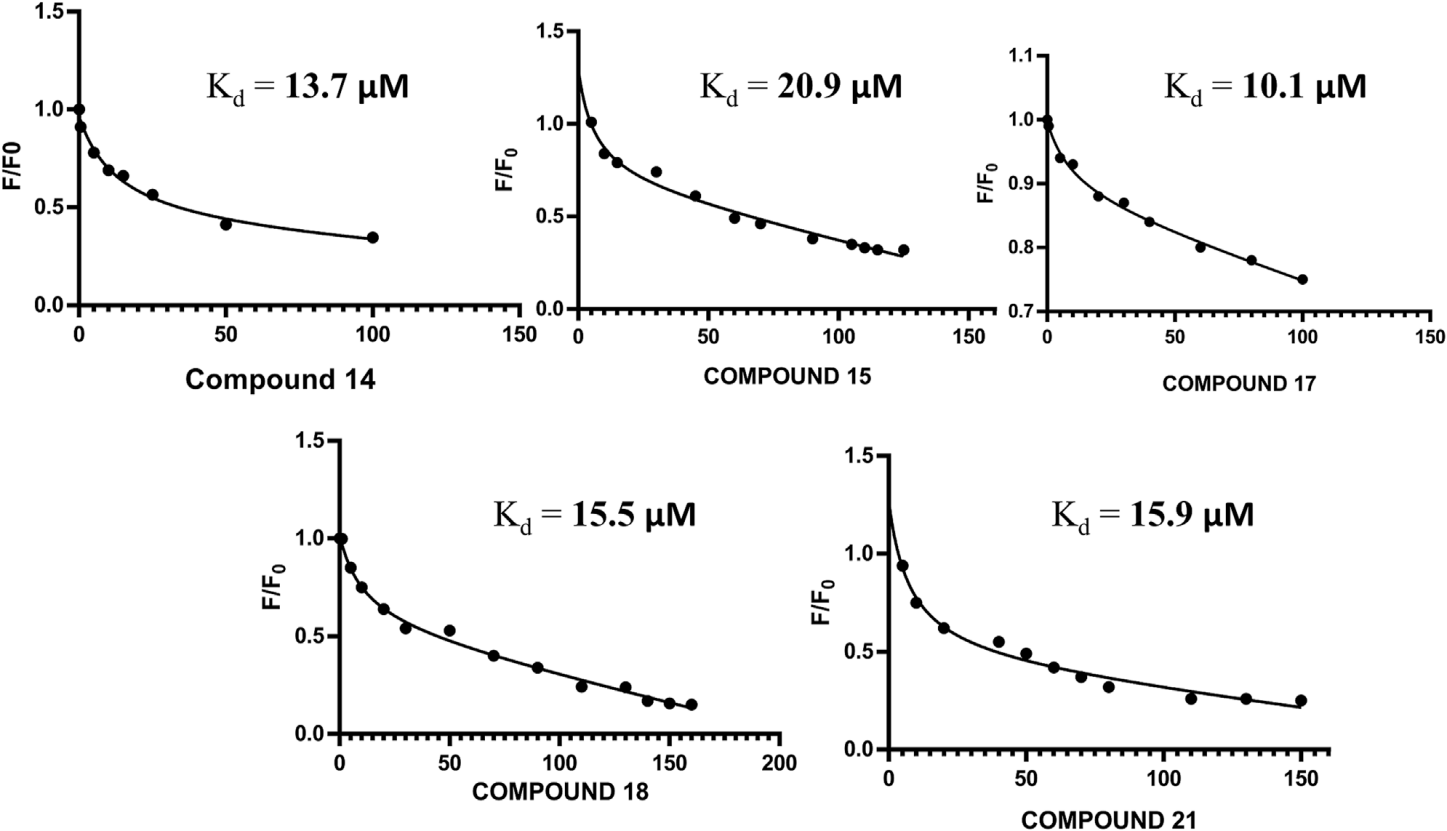
**(A)** Non-linear regression curve for compound **14** and FGF1 with calculated Kd of 13.70 μM. **(B)** Non-linear regression curve for compound **15** and FGF1 with calculated Kd of 20.9 μM. **(C)** Non-linear regression curve for compound **17** and FGF1 with calculated Kd of 10.1 μM. **(D)** Non-linear regression curve for compound **18** and FGF1 with calculated Kd of 15.50 μM. **(E)** Non-linear regression curve for compound **21** and FGF1 with calculated Kd of 15.9 μM.

The altered residues shown in the HSQC spectra may possibly constitute the binding sites of the derivatives. For all five compounds (**14**, **15**, **17**, **18**, and **21**), the intensities of the altered residues after titration with FGF1 are shown in Figure 2. They mostly constitute the same region of FGF1, which is the interface between FGF1 and FGFRD2 in the X-ray structure of the FGF1–FGFRD2 complex. In addition, derivatives including sulfonated naphthalene showed that five compounds mostly targeted the FGF1-heparin interface (Lozano et al., 1998; Sola et al., 1999; Zamai et al., 2002; Fernández-Tornero et al., 2003; Manetti et al., 2005). Binding between FGF1 and the five ligands was also supported by the fluorescence spectroscopy results, which showed that FGF1 binds to the five ligands (Figure 3).

### Structure–Activity Relationships

Taking suramin as the template, compounds carrying a replacement of the aryl sulfonic acid moieties by mono and trisulfonic acids of naphthalene from compounds **14–18** and the replacement of benzene by thiophene in compound **21** were incorporated in the suramin structure as shown in Figures 1A–E. Five compounds (**14–18** and **21**) were prepared for biological evaluation as described below in the WST-1 assays for cell proliferation and cytotoxicity. The derivatives (nitro and amino compounds) of the precursor were tested for interactions with FGF1 as shown in Figure 2. The interaction between the derivatives and FGF1 was determined using ^1^H-^15^N HSQC experiments. After analyzing the ^1^H-^15^N HSQC results, some derivatives displayed the chemical shift perturbation or peak intensity decrease. Considering these changes, HADDOCK simulations were performed and docking results of all the five derivatives (compounds **14**, **15**, **17**, **18,** and **21**) showed an interaction at the interface region blocking the interaction between FGF1 and its receptor FGFR2D2. To define biological activity of the compounds, we performed the water-soluble tetrazolium salt (WST-1) assay using the breast cancer cell line MCF7 to verify its effects on cell proliferation. After the addition of these five derivatives to FGF1 in the MCF7 cell cultures, cell proliferation was decreased compared to the addition of FGF1 alone. All nitro and amino derivatives bound to and blocked FGF1 activity by decreasing cell proliferation, which indicated an inhibition of the interaction of FGF1 with its receptor FGFRD2.

### Mechanism of Action of Compounds 14, 15, 17, 18, and 21

Fluorescence anisotropy has tremendous significance for biochemical application because it provides valuable knowledge about the probability of quencher activity. The anti-mitogenic activity of the five compounds was achieved by the same mechanism in which suramin inhibits the mitogenic activity of FGF1. A non-linear regression curve was plotted for the relative intensities of activity versus drug concentration using the Stern–Volmer equation (Eq. 1).

$$\log\frac{\left(F_{0}-F\right)}{F}=\log K+n\log\left[Q\right],$$ (1)

where F_0_ represents the fluorescence intensity in the absence of the compounds, and F represents the fluorescence intensity in the presence of the [Q] concentration of the compounds.

Increasing concentrations of these five suramin derivatives were added to a solution containing 5 µM FGF1. A reduction in the fluorescence intensity was reported for all five suramin derivatives. The Stern–Volmer equation was used to calculate the binding constant between FGF1 and compounds **14, 15, 17, 18,** and **21** for relative intensities versus the total concentration in a non-linear regression curve.

### Docking of FGF1 and Five Compounds and Their Complex Formation

The HADDOCK tool (van Zundert and Bonvin, 2014) is a modular method of docking based on the knowledge of bimolecular simulations. This tool can solve a wide variety of modeling problems encountered in different protein complexes, such as the ligand-protein complex and protein–protein complex. The core principle of HADDOCK is to use experiments to direct the docking and molecular refining of simulations (including, though not limited to, chemical cross linking, mutagenesis data, and NMR data) (van Zundert and Bonvin, 2014; Van Zundert et al., 2016; Imran Khan et al., 2019), such that:

$$HADDOCK_{score}=1.0*E_{vdw}+0.2*E_{elec}+1.0*E_{desol}+0.1*E_{air}.$$ (2)

The terms E_vdw_ (intermolecular van der Waals energy), E_elec_ (intermolecular electrostatic energy), E_desol_ (empirical desolvation energy), and E_air_ (ambiguous interaction restraint (AIR) energy) were adapted from the study by Fernandez et al. (de Vries. et al., 2007; Ezgi; Karaca, et al., 2010; Parveen et al., 2020).

HSQC NMR titration results were compared for the binding interface of FGF1with compounds **14**, **15**, **17**, **18**, and **21**. We found that the maximum number of residues showing peak disappearance/or perturbation after titration with the five suramin derivatives were located in the region of the binding interface between FGF1 and FGFRD2. The five complexes were simulated by structural modeling. The identification of ambiguous interaction restraints (AIRs) was based on differences in intensity from the HSQC results among the compounds and FGF1. The crystal structure of FGF1 for the HADDOCK calculations was obtained from PDB (1BAR), and the five compound PDB structures were created using PyMOL (DeLano, 2020).

**FIGURE 4:**
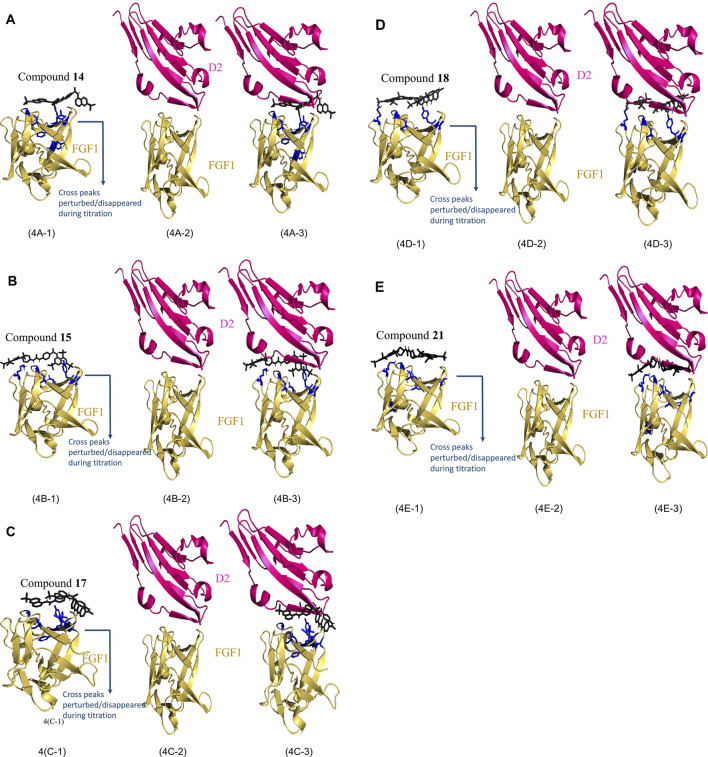
HADDOCK results of FGF1 with the five compounds. **(4A)**: **(4A-1)** Calculated HADDOCK of Compound **14** (black) complex with FGF1 (Yellow) with AIR painted in blue, **(4A-2)** X-ray crystallographic structure of FGF1 and D2 (PDB ID: 3CU1). **(4A-3)** overlap of **(4A-1)** and **(4A-2)**. **(4B)**: **(4B-1)** Calculated HADDOCK of Compound **15** (black) complex with FGF1 (Yellow) with AIR painted in blue, **(4B-2)** X-ray crystallographic structure of FGF1 and D2 (PDB ID: 3CU1). **(4B-3)** overlap of **(4B-1)** and **(4B-2)**. **(4C)**: **(4C-1)** Calculated HADDOCK of Compound **17** (black) complex with FGF1 (Yellow) with AIR painted in blue, **(4C-2)** X-ray crystallographic structure of FGF1 and D2 (PDB ID: 3CU1). **(4C-3)** overlap of **(4C-1)** and **(4C-2)**. **(4D)**: **(4D-1)** Calculated HADDOCK of Compound **18** (black) complex with FGF1 (Yellow) with AIR painted in blue, **(4D-2)** X-ray crystallographic structure of FGF1 and D2 (PDB ID: 3CU1). **(4D-3)** overlap of **(4D-1)** and **(4D-2)**. **(4E)**: **(4E-1)** Calculated HADDOCK of Compound **21** (black) complex with FGF1 (Yellow) with AIR painted in blue, **(4E-2)** X-ray crystallographic structure of FGF1 and D2 (PDB ID: 3CU1). **(4E-3)** overlap of **(4E-1)** and **(4E-2)**.

The best 200 structures were refined on the basis of their overall lower energy. All 200 complexes were divided into approximately seven clusters for each compound. The first cluster was the most robust due to a high degree of similarity and lower energy of refined water interactions than the other clusters. The HADDOCK complex between FGF1 and the compounds is shown in Figures 4A-1(FGF1 complexed with compound **14**), Figure 4B-1 (FGF1 complexed with compound **15**), Figure 4C-1 (FGF1 complexed with compound **17**), Figure 4D-1 (FGF1 complexed with compound **18**), and Figure 4E-1 (FGF1 complexed with compound **21**). When these HADDOCK complexes overlapped with the X-ray crystal structure of the FGF1-D2 complex (PDB:3CU1) Figures 4A-2,B-2,C-2,D-2,E-2, it generated tertiary complexes as shown in Figures 4A-3,B-3,C-3,D-3,E-3) with respect to the different compounds **14**, **15**, **17**, **18**, and **21**. All five compounds effectively blocked the interaction between FGF1 and its receptor D2 of FGFRD2 (Figure 4). The figure shows an overlapped view of the ternary complex of FGF1, the five compounds, and D2 of FGFRD2. These five suramin derivatives block in between the FGF1 and D2 and interrupted the signal transduction cascade and, thus, could interfere with downstream cell proliferation.

In the docking calculations the binding energies between suramin derivatives and proteins were calculated using PRODIGY HADDOCK software. PRODIGY is a webserver used for predicting the protein–protein complex binding affinity, which was initially limited to protein–protein complex interactions, and later was extended to the protein-ligand complex in the PRODIGY-LIG module. The latter predicted the protein-ligand complex affinity by atomic contacts inspite of amino acid contacts. Most of the atomic contacts within a 10.5 Å distance cutoff between the ligand and the protein are considered and grouped on the basis of their atomic interaction (N-nitrogen, C-carbon, O-oxygen, and X-all other atoms). The final predictor models of the binding energy ∆G_score_ and ∆G_prediction_ used to predict the binding affinities and ranking ligands, respectively, are as follows:

∆G_score_ = 0.343794 + E_elec_−0.037597 * AC_CC_ + 0.138738 * AC_NN_ + 0.160043 * AC_OO_−3.088861 * AC_XX_ + 187.011384.

∆G_prediction_ = 0.0115148 * E_elec_−0.0014852 AC_CC_ + 0.0057097 * AC_NN_−0.1301806 * AC_XX_−5.1002233.

AC_CC_–(atomic contact between carbon and carbon), AC_NN_ - (atomic contact between nitrogen and nitrogen), AC_OO_–(atomic contact between oxygen and oxygen), AC_XX_–(atomic contact between all other atoms).

In PRODIGY-LIG, only atomic contacts are considered, and thus, only ∆G_noelec_ was obtained after using this program.

∆G_noelec_ = 0.0354707 * AC_NN–_0.1277895 * AC_CC_ −0.0072166 * AC_CN_ −5.1923181 (Anna Vangone et al., 2019).

The PRODIGY-LIG binding energy calculated for all the five complexes is shown below

FGF1-comp **14**, ∆G_noelec_ = −7.6 kcal/mol, FGF1-comp **15**, ∆G_noelec_ = −8.3 kcal/mol, FGF1-comp 1**7**, ∆G_noelec_ = −13.8 kcal/mol, FGF1-comp **18**, ∆G_noelec_ = −8.3 kcal/mol, FGF1-comp **21**, ∆G_noelec_ = −11.5 kcal/mol.

The interactions between the FGF1 and the five compounds were hydrophobic interactions and in addition, hydrogen bond interaction was identified between the compounds and the protein.

### Validation of the HADDOCK Protocol

HADDOCK which has been implemented in the CNS is used to calculate structure interactions using Python scripts. The docking follows the three steps, 1) randomization of orientations and rigid body energy minimization, 2) semi-rigid simulated annealing in torsion angle space (TAD-SA), and 3) final refinement in the Cartesian space with an explicit solvent. The average interaction energies such as van der Waals, desolvation and electrostatic energies, and buried surface area were used to finalize the final structures. HADDOCK simulations were carried out for the five ligand complexes using ^1^H-^15^N HSQC perturbation, and the intensity of the decrease in data defined the ambiguous interaction restraints. Only the active ambiguous interaction restraints were used for the HADDOCK simulations. The complexes obtained from the HADDOCK stimulations are shown in Figure 4, and were validated by Ramachandran plots. Ramachandran plots were obtained by the RAMPAGE server (https://zlab.umassmed.edu/bu/rama/where the favored and disallowed regions were calculated. The residues in the favored region for the complex FGF1-comp**14** were 99.065%, complex FGF1-comp**15** were 92.523%, complex FGF1-comp**17** were 97.342%, complex FGF1-comp **18** were 98.131%, and for the complex FGF1-comp**21** were 96.177%. The Ramachandran plots for all the five complexes are shown in supporting information Supplementary Figure S6.

### Analysis of Cell Proliferation

**FIGURE 5:**
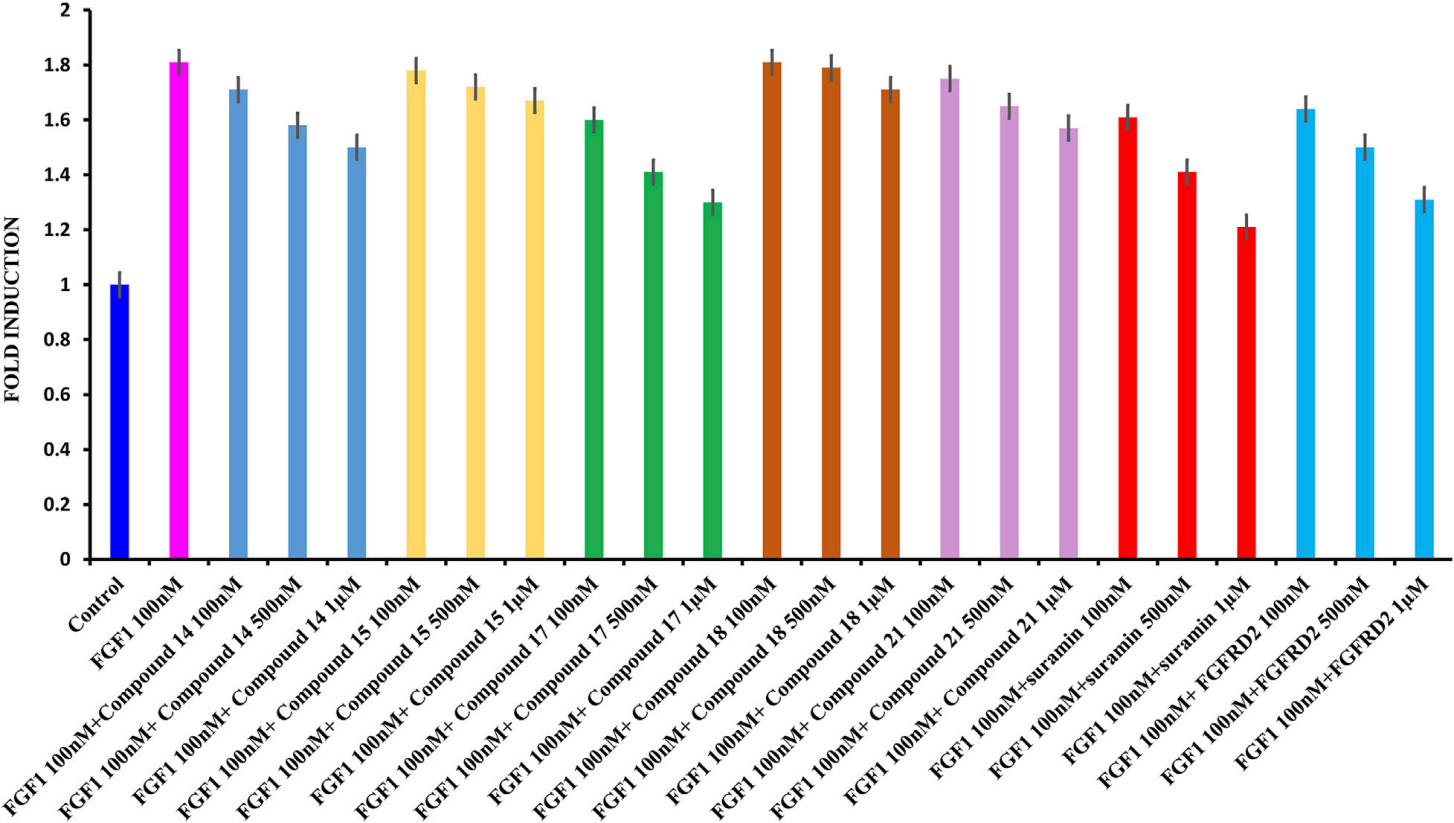
WST1 ASSAY results for cell proliferation. **(A)**: From left to right: control in blue; FGF1 in pink (100 nM) and 100 nM, 500 nM, and 1 μM of compound **14** in blue; 100 nM, 500 nM, and 1 μM of compound **15** in yellow; 100 nM, 500 nM, and 1 μM of compound **17** in green, 100 nM, 500 nM, and 1 μM of compound **18** in brown, 100 nM, 500 nM, and 1 μM of compound **21** in light pink, 100 nM, 500 nM, and 1 μM of suramin in red, and 100 nM, 500 nM, and 1 μM of D2 in cyan.

Cell proliferation was measured using the WST-1 assay. The breast cancer cell line MCF-7 was used, which is known to express the FGFR. As shown in Figure 5, treating serum-starved MCF-7 cells with 100 nM FGF1 significantly stimulated cell proliferation of breast cancer cells. To determine the effects of suramin and its derivatives (compounds **14**, **15**, **17**, **18**, and **21**) on the disruption of FGF1–EGFRD2-mediated cellular activity, increasing concentrations of 100 nM, 500 nM, and 1 µM were used to treat cells in combination with FGF1. As shown in Figure 5, the results revealed that the FGF1-stimulated cell proliferation could be attenuated by co-treatment with all five suramin derivatives in a dose-dependent manner. Compounds **17** and **14** showed more potent inhibitory effects on FGF1-stimulated cell proliferation. A similar phenomenon was also observed for the suramin-treated group (Figure 5). Exogenous recombinant FGFRD2 protein was used as the control, as the inhibitor could block the interaction between FGF1 and endogenous FGFR2D2 on the cell membrane. A decrease in the cell number was observed in the exogenous FGFRD2 competition group (Figure 5). These data indicated that suramin and its derivatives attenuated FGF1-stimulated cell proliferation likely through the disruption of the interaction between FGF1 and FGFRD2. The WST-1 assay was used to analyze cytotoxicity.

**FIGURE 6:**
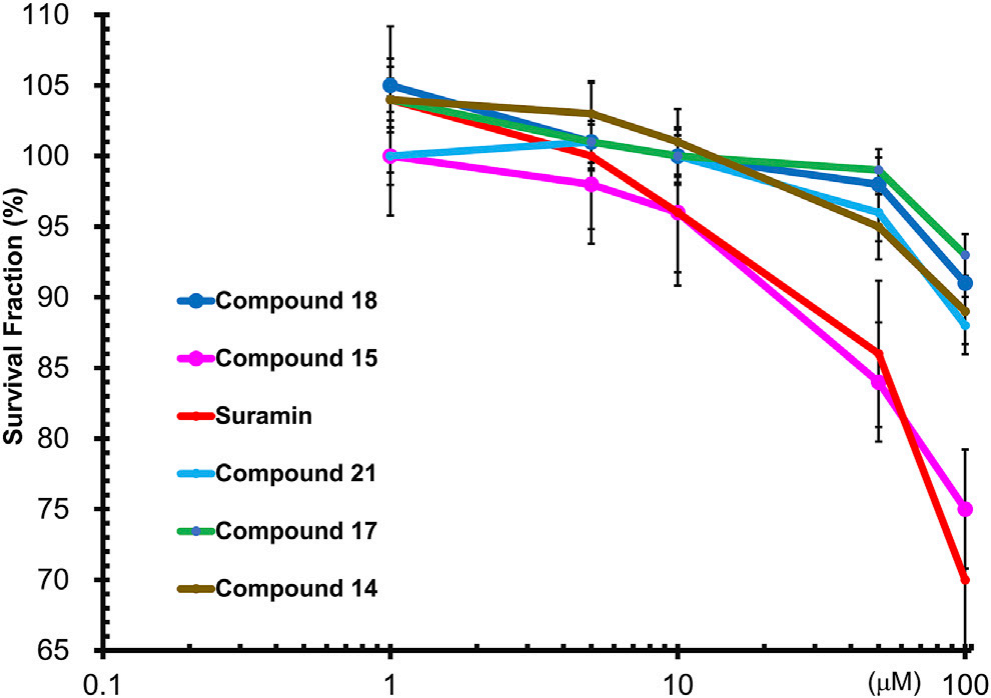
Wst1-Assay results of cytotoxicity of FGF1 with five compounds (**14, 15, 17, 18** and **21**): Compound **14** in brown, Compound **15** in pink, Compound **17** in green, Compound **18** in blue, Compound **21** in cyan, and suramin in red.

To determine the cytotoxic effects of suramin and its derivatives, compounds **14**, **15**, **17**, **18,** and **21**, with increasing concentrations of each compound (1, 5, 10, 50, and 100 µM) were used to treat MCF-7 cells for 48 h, and the WST-1 assay was performed. The results showed that compounds **14**, **15**, **17**, **18**, and **21** were less toxic to MCF-7 cells than to suramin, especially at concentrations higher than 10 µM (Figure 6). The cytotoxicity of suramin was the highest among all the analogs. The reason for the higher cytotoxicity was the presence of methyl groups on the suramin structure (Martello et al., 2001).

Suramin-induced neuropathy may depend on the effects on calcium (Ca^2+^) homeostasis as a potential pathological mechanism, and the full or partial positive charge of the element when directly attached to the ring at each of the sulphonic groups (HSO_3_). These interactions exert a moderate to strong electron-withdrawing inductive effect, which may be responsible for the observed suramin cytotoxicity (Prasetyaningrum et al., 2018; Von Der Ahe et al., 2018). The cytotoxicity of the synthesized analogs was lower as there were methyl groups and no sulfonic acid groups attached to the suramin backbone. The IC50 values of the synthesized compounds in MCF-7 breast cancer cells were analyzed using SigmaPlot software. The estimated IC50 values of suramin and its derivatives in MCF-7 cells are as follows: suramin: 153.96 ± 1.44 μM, compound **14**: 408.8 ± 3.08 μM, compound **15**: 193.04 ± 1.57 μM, compound **17**: 619.4 ± 1.73 μM, compound **18**: 325.63 ± 2.03 μM, and compound **21**: 417.01 ± 0.99 μM.

In order to gain some insight about the selectivity of the compounds between tumorigenic and non-tumorigenic cells, the cytotoxicity was also being evaluated against normal breast cell lines by examining the cytotoxicity of suramin and its derivatives, compounds **15, 18,** and **21**, in a normal breast epithelial cell line (non-tumorigenic), MCF-10A. The results revealed that not only suramin but also the derivatives were not very toxic (IC50 > 100 μM) to the non-tumorigenic MCF-10A cells (Supplementary Figure S7). Compared to their cytotoxicity in tumorigenic MCF-7 cells (Figure 6), the estimated IC50 values in MCF-10A were higher than those in MCF-7 cells. The estimated IC50 values in non-tumorigenic cells (MCF-10A) vs. those in tumorigenic cells (MCF-7) were as following: suramin: 222.10 ± 2.40 μM vs. 153.96 ± 1.44 μM; compound **15**: 220.84 ± 3.57 μM vs. 193.04 ± 1.57 μM; compound **18**: 653.39 ± 2.31 μM vs. 325.63 ± 2.03 μM; and compound **21**: 628.04 ± 1.45 μM vs. 417.01 ± 0.99 μM. However, the estimated IC50 value of suramin and its derivatives in both non-tumorigenic MCF-10A and tumorigenic MCF-7 cells were over 100 μM. The effective concentrations of suramin and its derivatives to attenuate FGF1-stimulated cell proliferation in MCF-7 cells were around 1 μM (Figure 5), which is non-toxic concentration to both MCF-7 cells and MCF-10A cells. Previous reports have demonstrated that FGFR gene loci were amplified and overexpressed in different subtypes of breast cancers (Maria Francesca Santolla, 2020). Therefore, in addition to the specificity of each drug, the selectivity of suramin and its derivatives to disrupt the FGF1/FGFR-mediated breast cancer growth might rely on the overexpression or amplification of FGFR2 in breast cancer cells, but not in normal cells, and the secreted FGF1 ligand by autocrine or paracrine effects from cancer cells or their surrounding stroma cells or adipocytes (tumor microenvironment). The statistical data for the cytotoxicity are shown in Supplementary Figure S8.

### Limitations

The *ex vivo* cell line model was a simpler and direct method to demonstrate the functional effects of the suramin derivatives when compared to an *in vivo* animal model (Chan et al., 2016). We used the MCF-7 breast cancer cell line as an *ex vivo* model to evaluate cell proliferation and cytotoxicity (Figures 5, 6). Historically, human tumor-derived cell lines play a crucial role in the development, design, and discovery of novel therapies for cancer. The *ex vivo* cell line model is widely used in preclinical drug screening and discovery using different human cancer-derived cell lines. For example, 60 different human tumor cell lines were used to develop a high-throughput platform and the NCI-60 human tumor cell line screen, to identify and characterize novel compounds with growth inhibition or killing of tumor cell lines (NCI). However, there are limitations in the *ex vivo* cell line model. The results from *ex vivo* cell line models need to be validated by *in vivo* animal models, including genetically engineered mouse models (Kersten et al., 2017), mice xenograft models, and patient-derived xenograft (PDX) models (Koga, 2019) to better determine their effects on human tumorigenesis and as new agents for anticancer therapy. In addition, the cancer organoid model is an emerging approach to develop personalized anticancer therapy to reduce the use of mice (Fan et al., 2019). The validated drug candidates will be further verified in clinical trials.

## Data Availability

The datasets presented in this study can be found in online repositories. The names of the repository/repositories and accession number(s) can be found in the article/Supplementary Material.

## Abbreviations

FGF, fibroblast growth factor; FGFR, fibroblast growth factor receptor; HSQC, heteronuclear single quantum coherence; MCF7, Michigan Cancer Foundation-7.
