# Supplementary material for: Synthesis of Novel Suramin Analogs With Anti-Proliferative Activity via FGF1 and FGFRD2 Blockade
Source: Front Chem. 2022 Jan 3;9:764200. doi: 10.3389/fchem.2021.764200 (PMC8763243; doi:10.3389/fchem.2021.764200)
Supplement: Supplementary file 1 [file DataSheet1.docx]

**Supporting information**

**Synthesis of Novel Suramin Analogs with Anti-Proliferative Activity via FGF1 and FGFRD2 blockade**

**Nuzhat Parveen,^1^ Yan-Liang Lin,^2^**

1. Chemistry Department, National Tsing Hua University, Hsinchu 300, Taiwan.
2. Department of Applied Chemistry, National Yang Ming Chiao Tung University, Hsinchu 300, Taiwan.

*** Correspondence:**

**Chin Yu:** [cyu.nthu@gmail.com](mailto:cyu.nthu@gmail.com), **Chung-Ming Sun:** [cmsun@nctu.edu.tw](mailto:cmsun@nctu.edu.tw), **Ruey-Hwang Chou:** [rhchou@gmail.com](mailto:rhchou@gmail.com)

**Ruey-Hwang Chou,^1,2,3,4*^ Chung-Ming Sun,^5,6*^ Chin Yu^7*^**

1. Graduate Institute of Biomedical Sciences, China Medical University, Taichung 40402, Taiwan, R.O.C
2. The Ph.D. Program of Biotechnology and Biomedical Industry, China Medical University, Taichung 40402, Taiwan, R.O.C.
3. Center for Molecular Medicine, China Medical University Hospital, Taichung 40402, Taiwan, R.O.C.
4. Department of Medical Laboratory and Biotechnology, Asia University, Taichung 41354, Taiwan, R.O.C.
5. Department of Medicinal and Applied Chemistry, Kaohsiung Medical University, Kaohsiung 807, Taiwan.
6. Department of Applied Chemistry, National Yang Ming Chiao Tung University, Hsinchu 300, Taiwan.
7. Chemistry Department, National Tsing Hua University, Hsinchu 300, Taiwan.

*authors to whom correspondence should be addressed.

Prof. Chin Yu^*^

Department of Chemistry

National Tsing Hua University Hsinchu, Taiwan.

Email: [cyu.nthu@gmail.com](mailto:cyu.nthu@gmail.com)

Prof. Chung Ming Sun*

Department of Applied Chemistry

National Chiao Tung University Hsinchu, Taiwan.

Email: [cmsun@nctu.edu.tw](mailto:cmsun@nctu.edu.tw)

Prof. Ruey-Hwang Chou*

Associate Professor, Graduate Institute of Biomedical Sciences,

Center for Molecular Medicine,

China Medical University,

9F, No. 6, Hsueh-Shih Road, Taichung, 40454, Taiwan

Tel:  886-4-22052121 ext. 7935 (Office) or 7913 (Lab)

Fax: 886-4-22333496

E-mail: [rhchou@cmu.edu.tw](mailto:rhchou@mail.cmu.edu.tw)


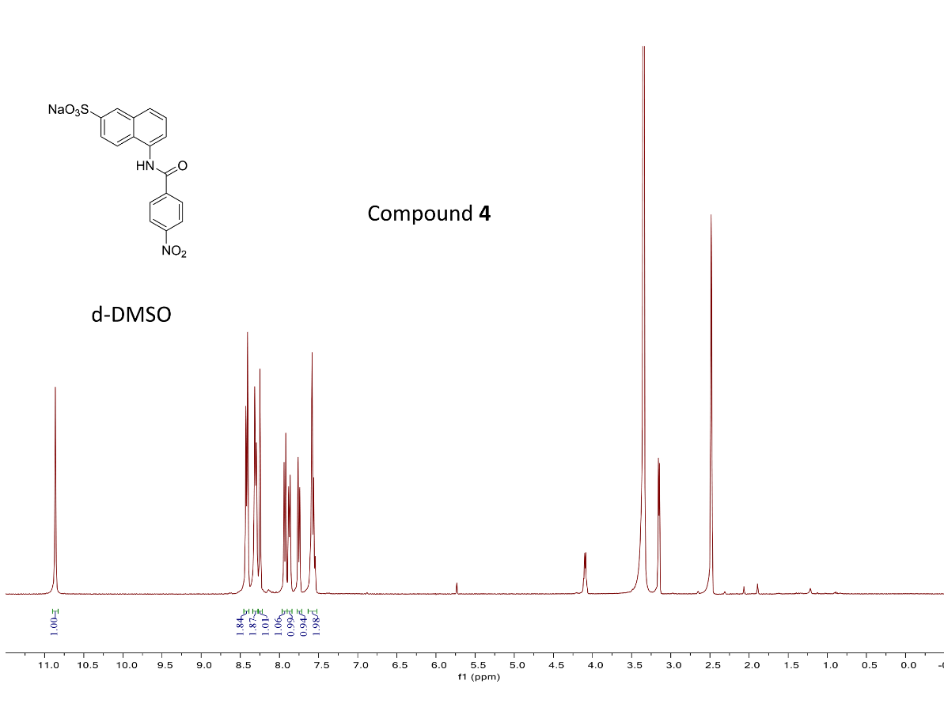

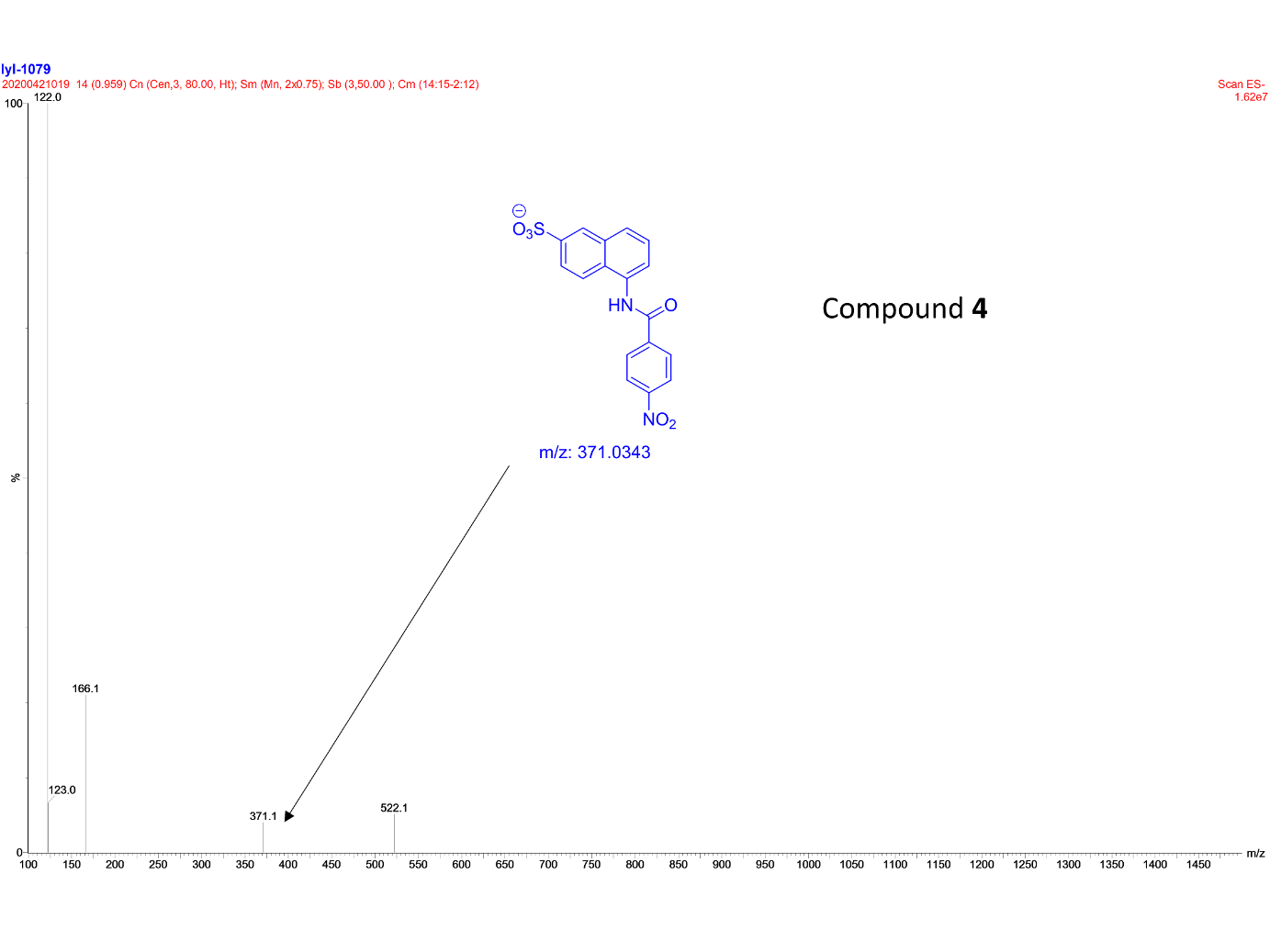


**S1.NMR Results of all the Compounds (compound 4-compound21)**


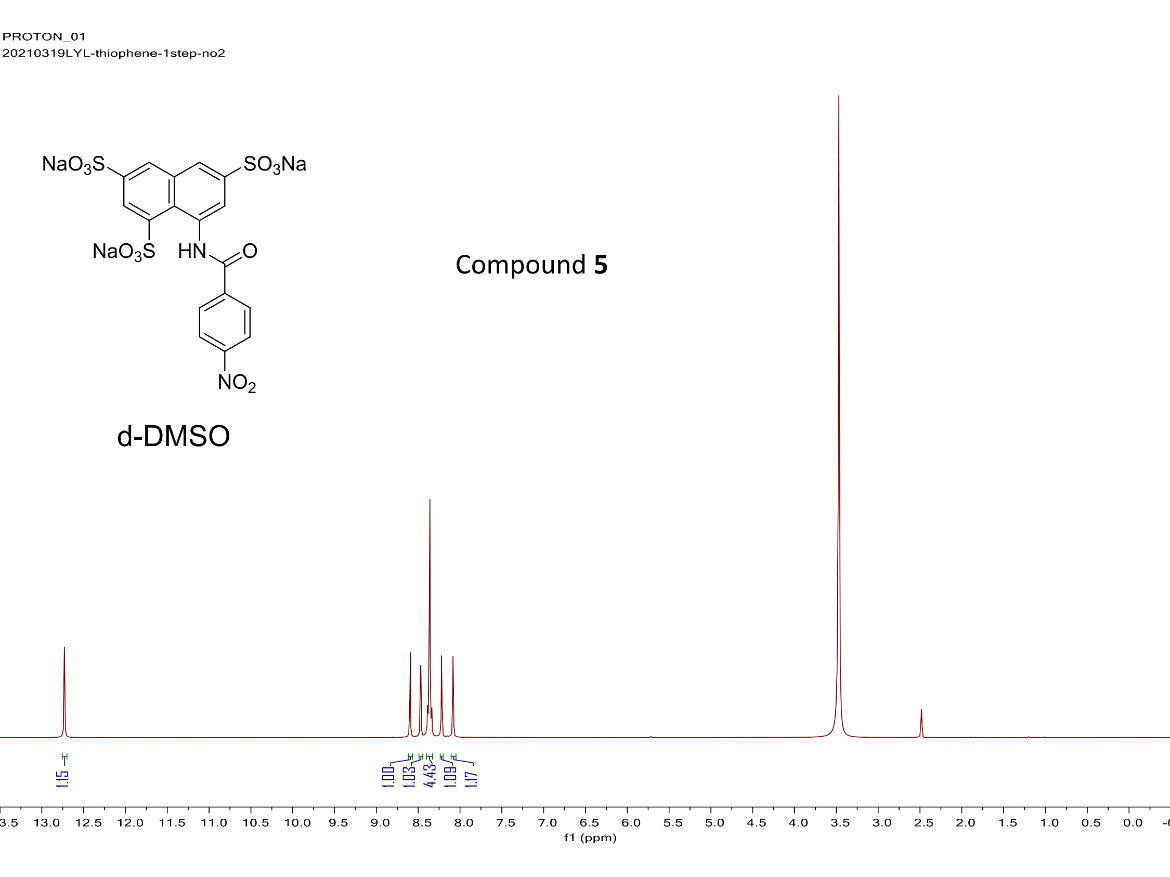


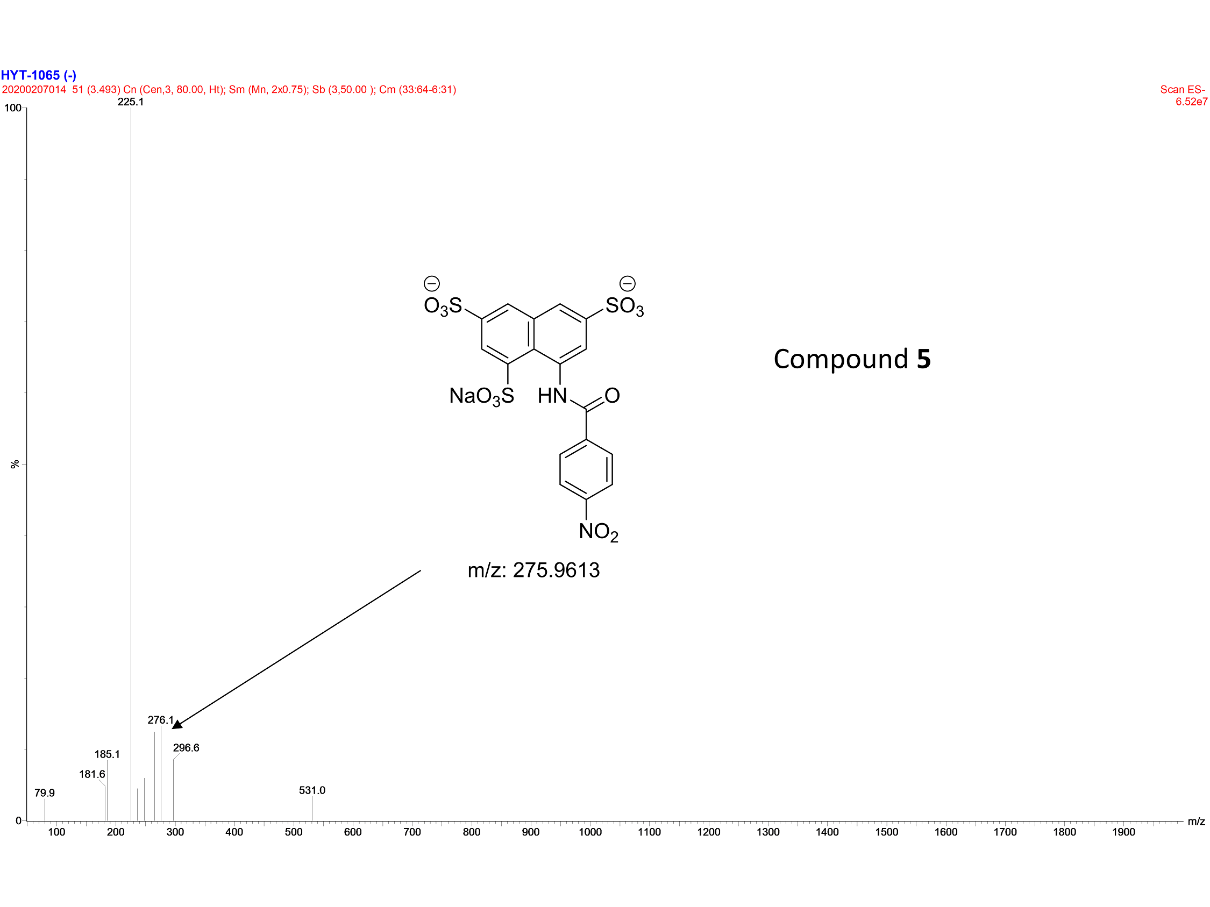


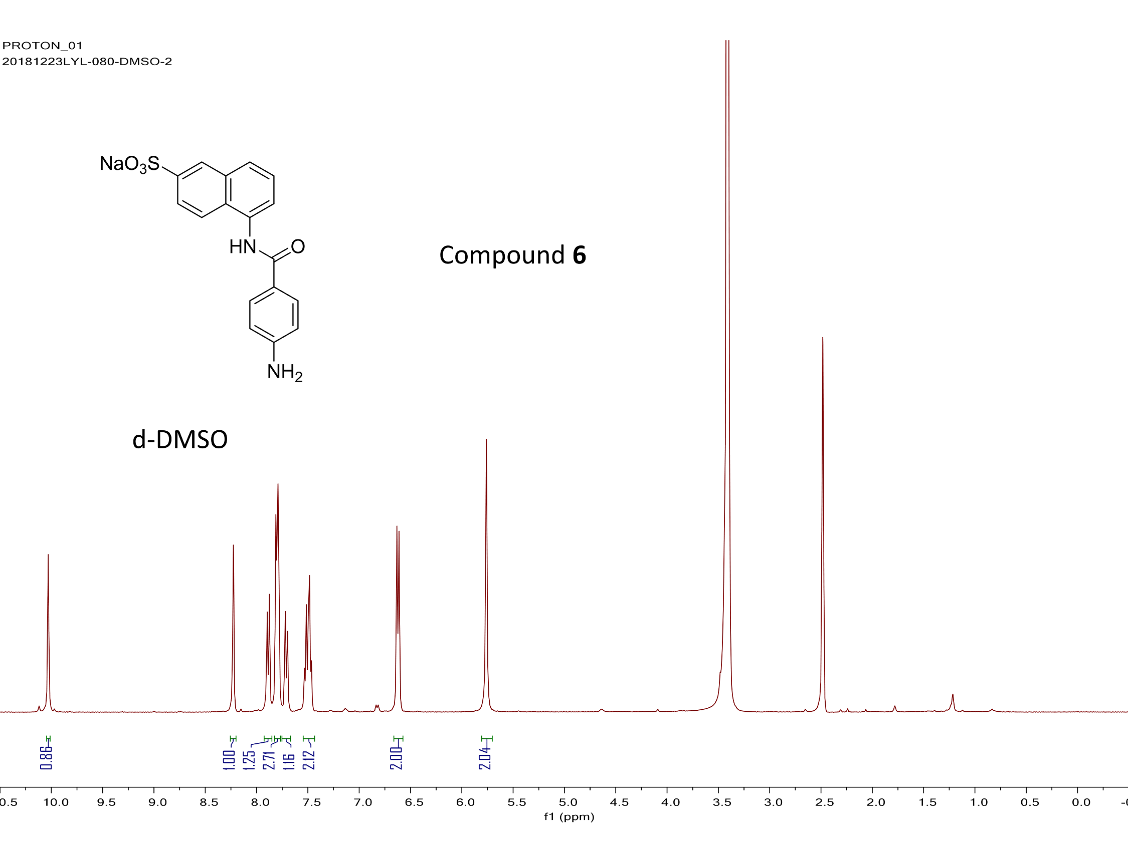


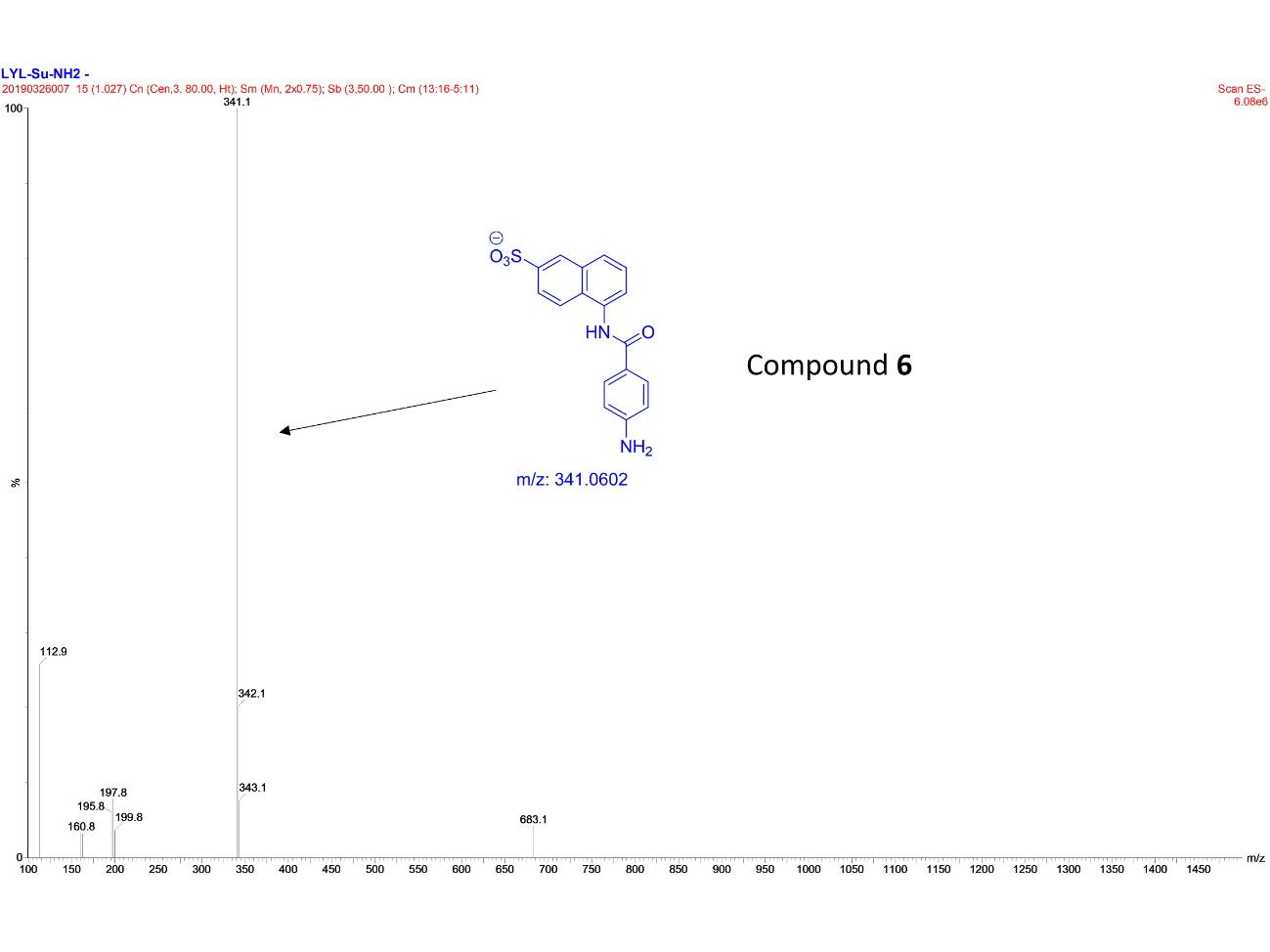


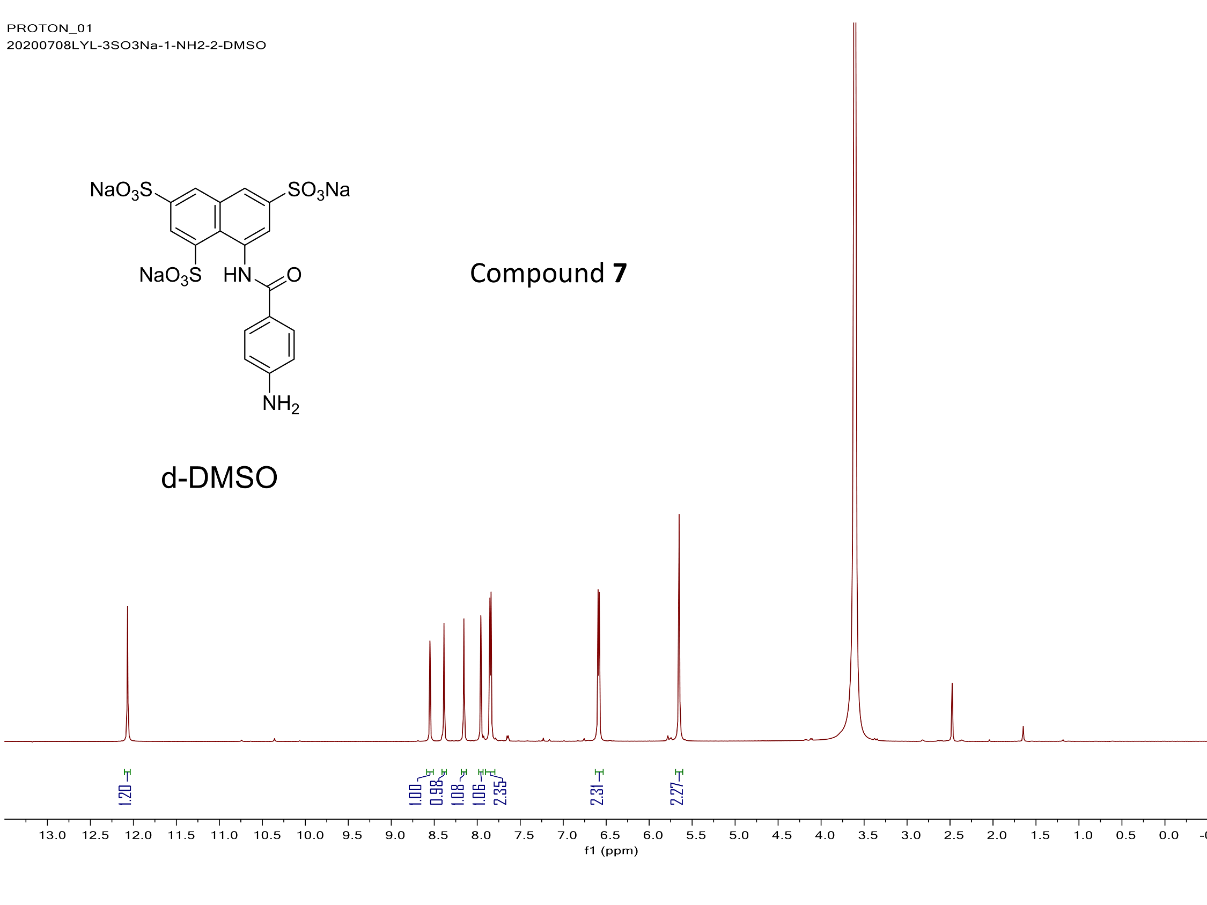


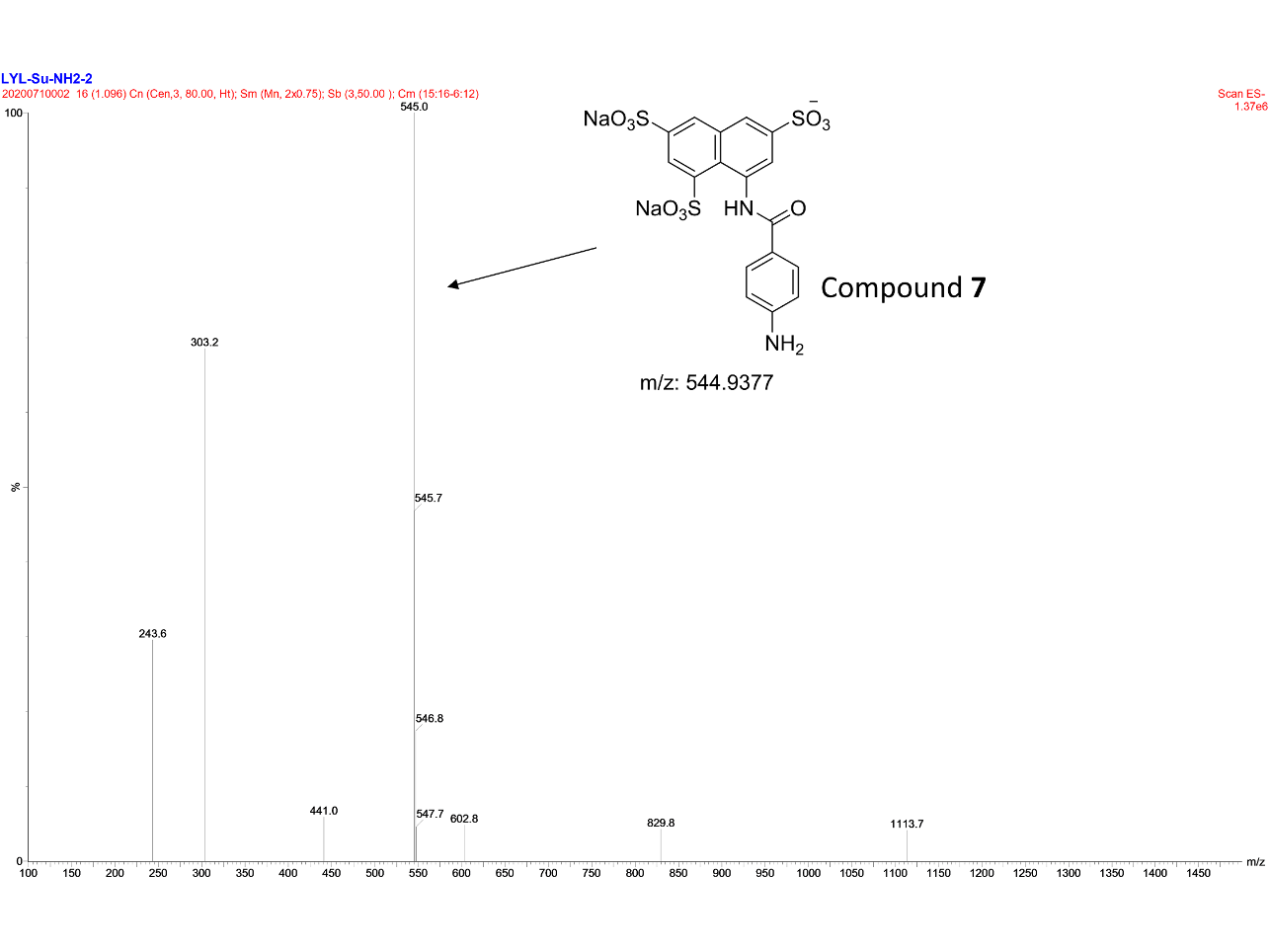


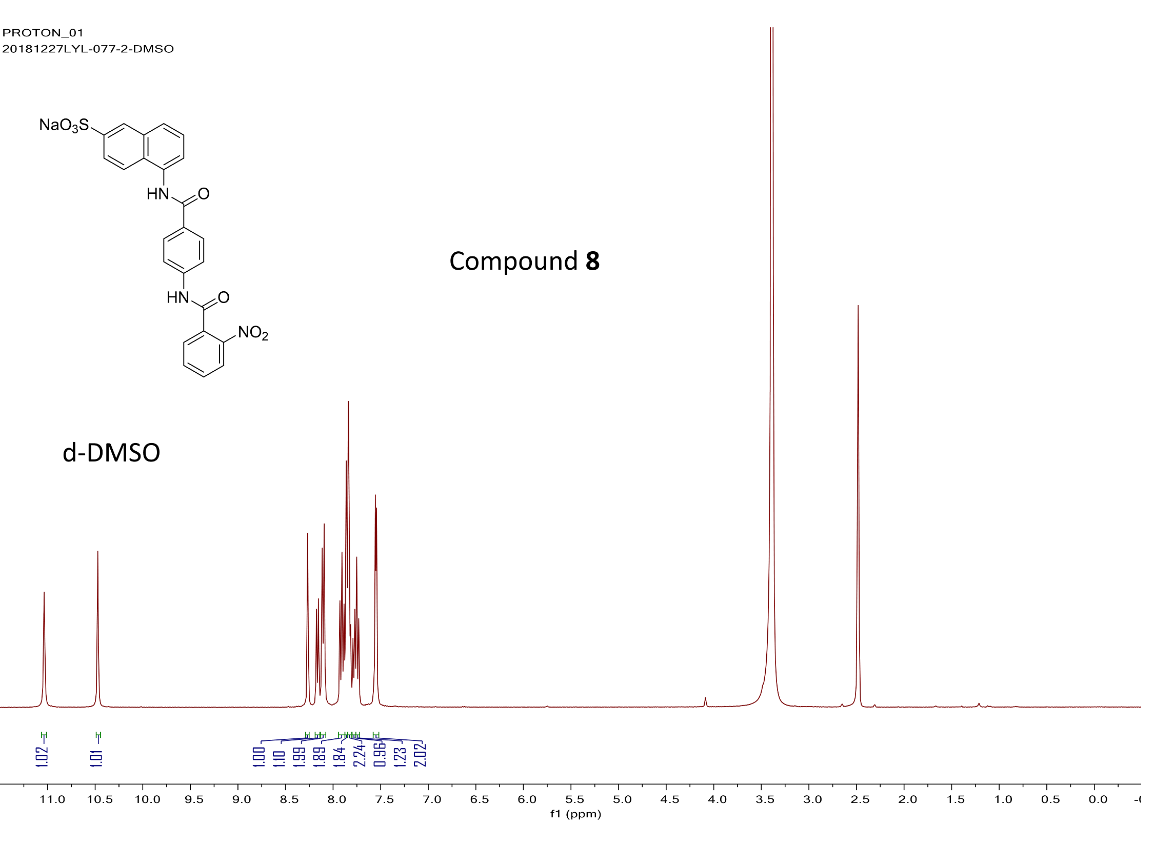


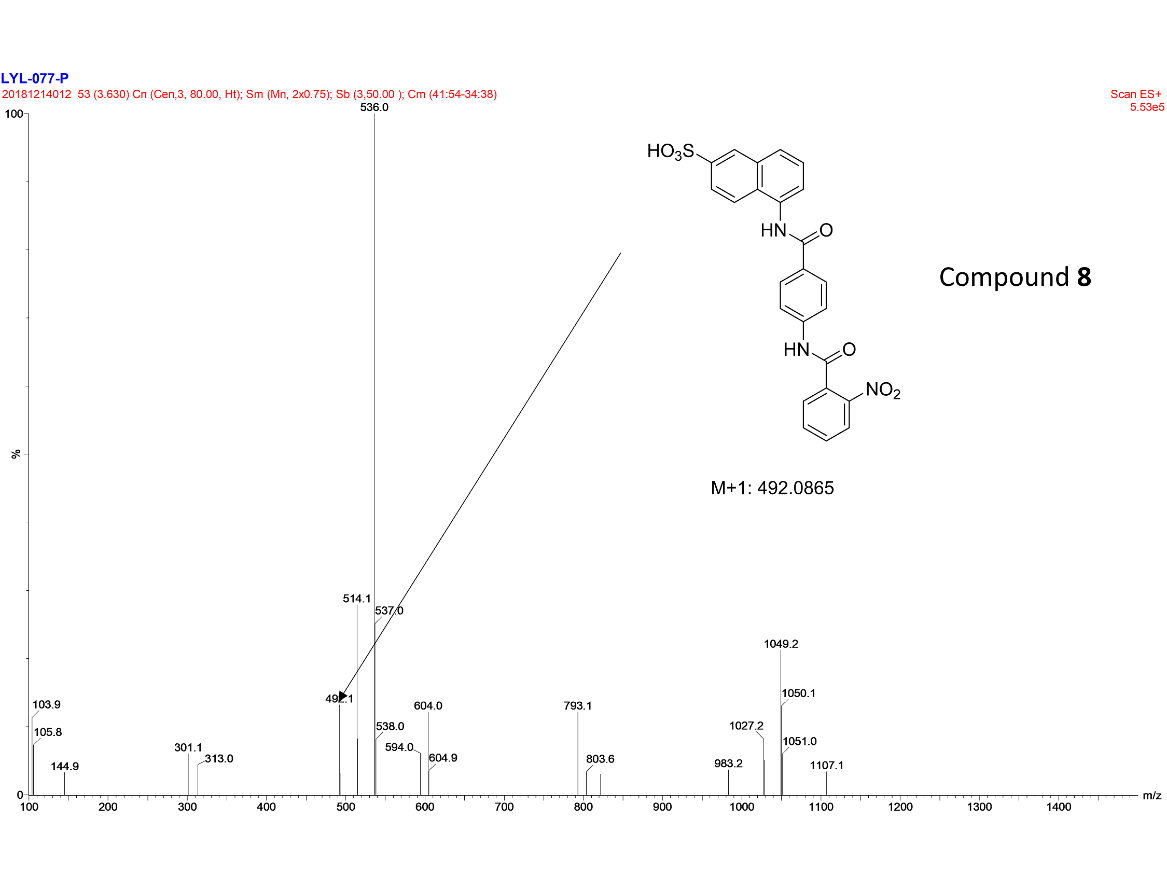


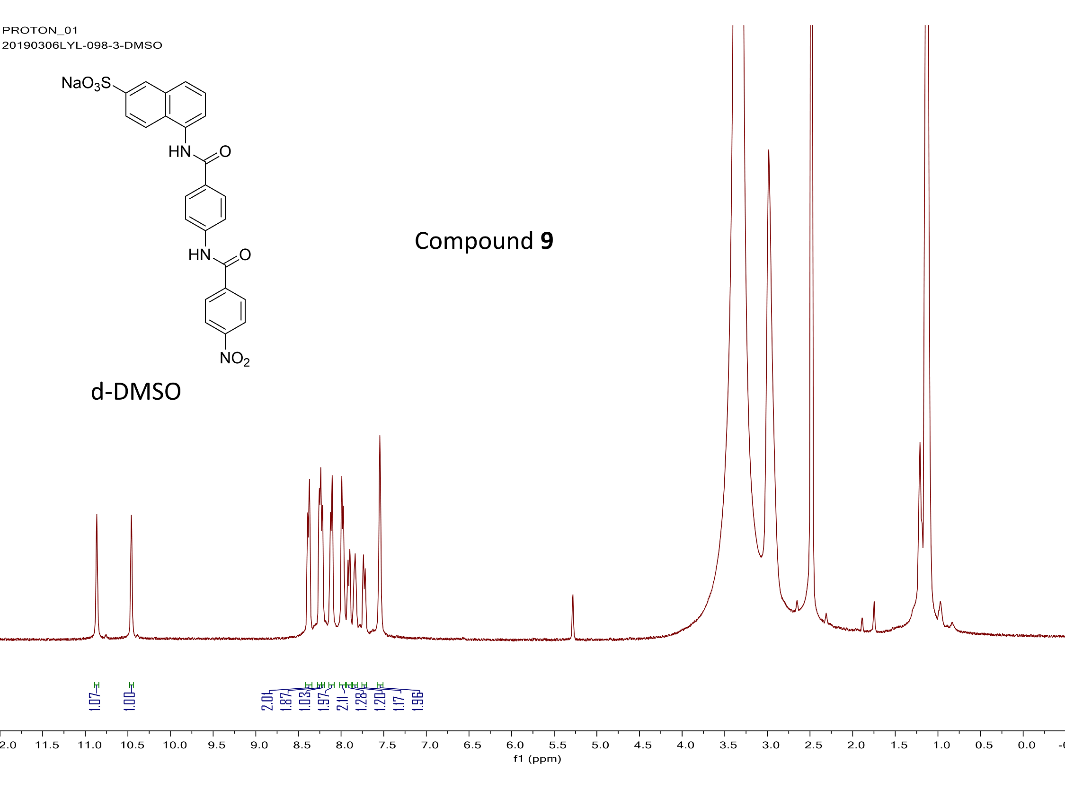


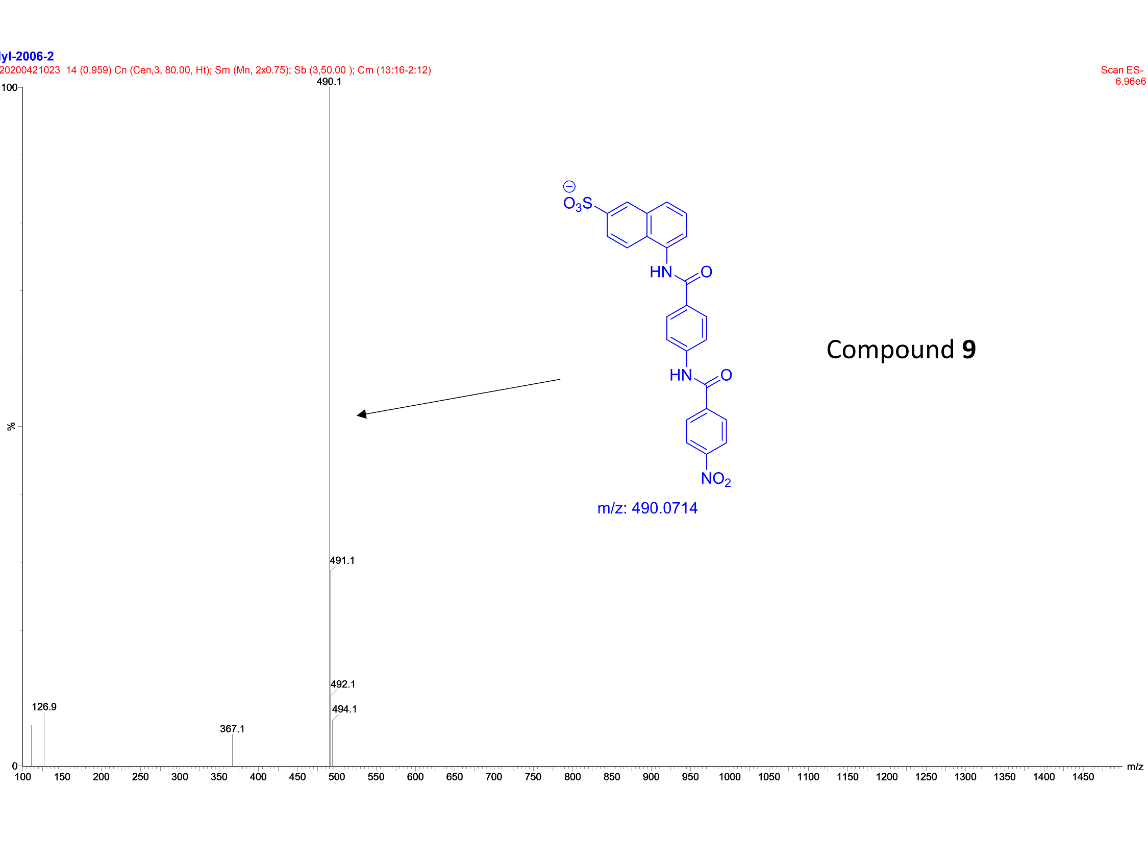


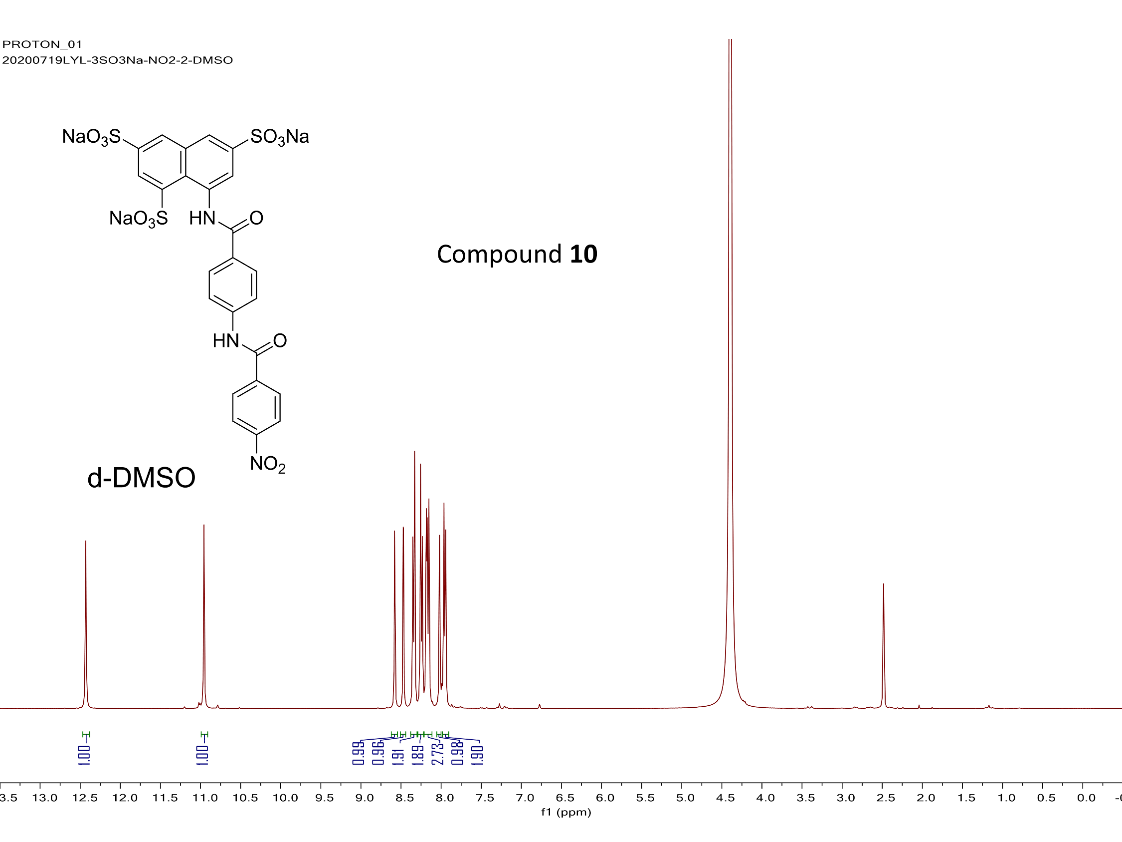


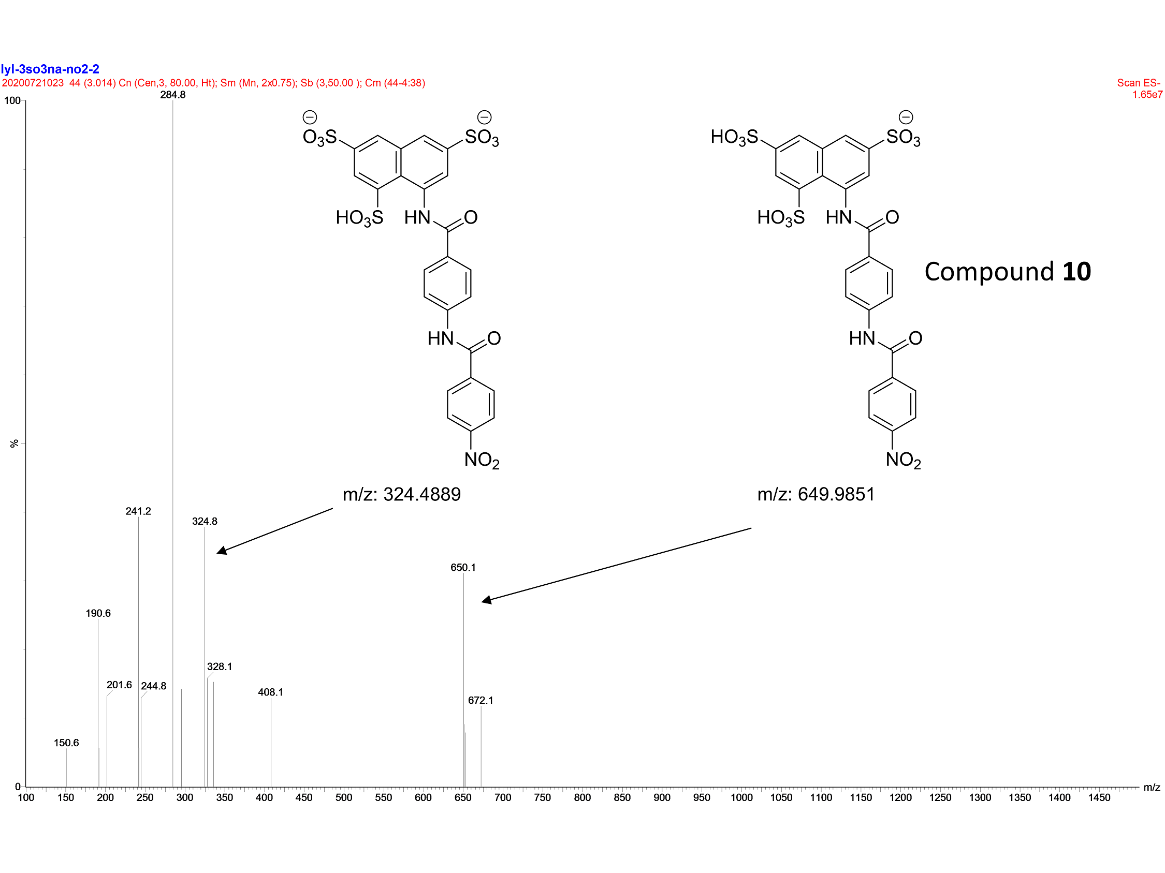


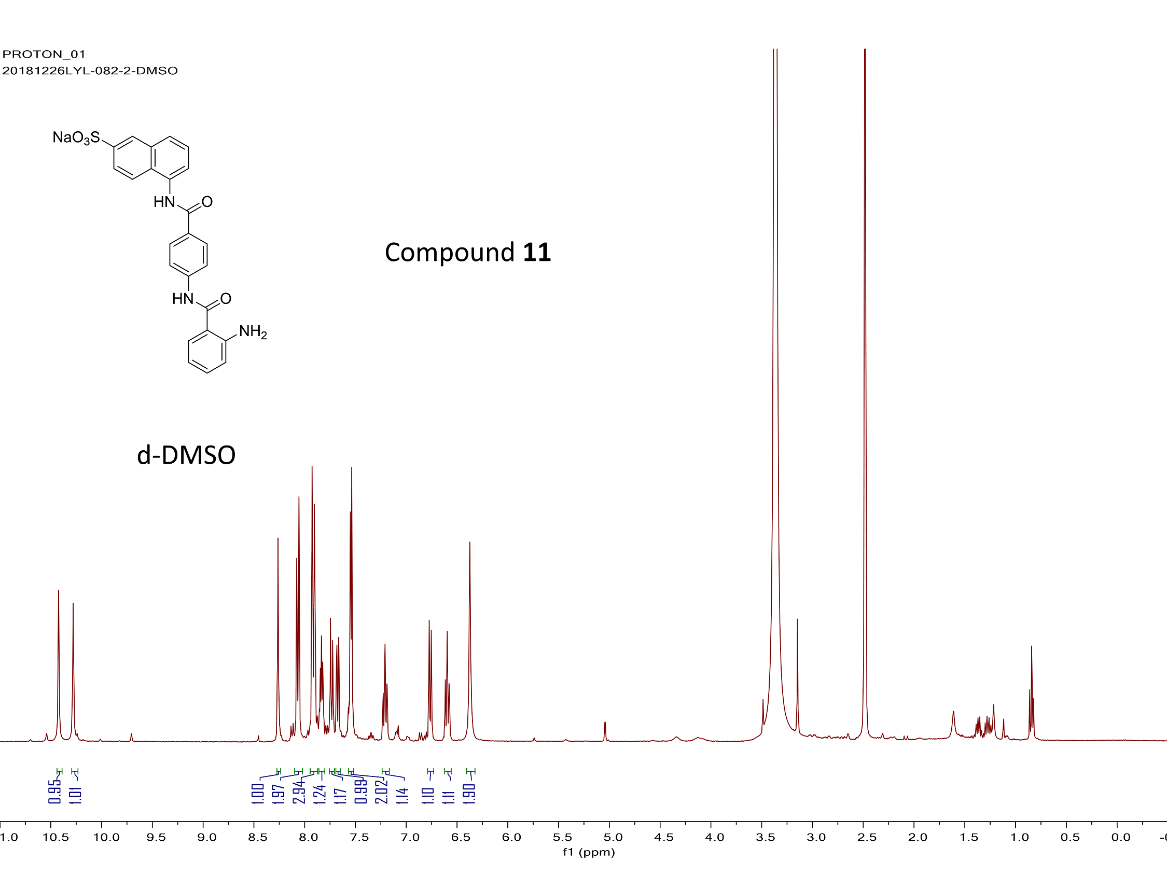


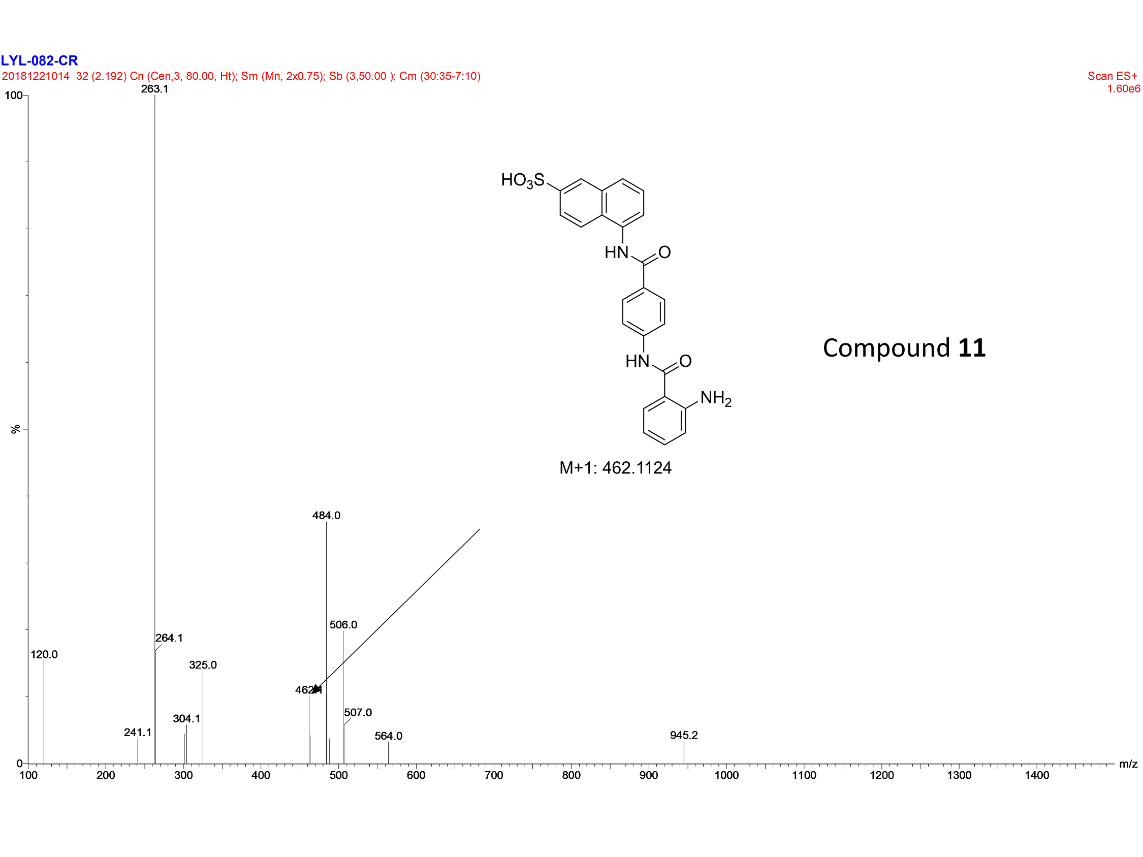


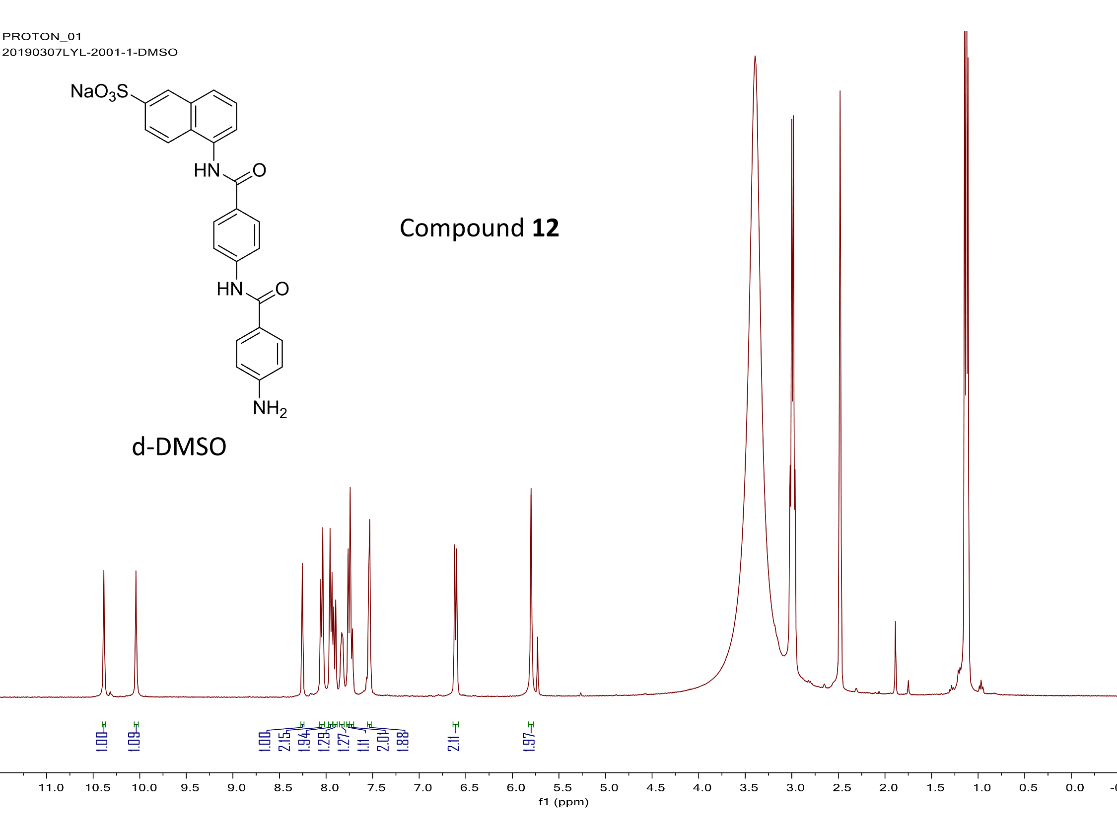


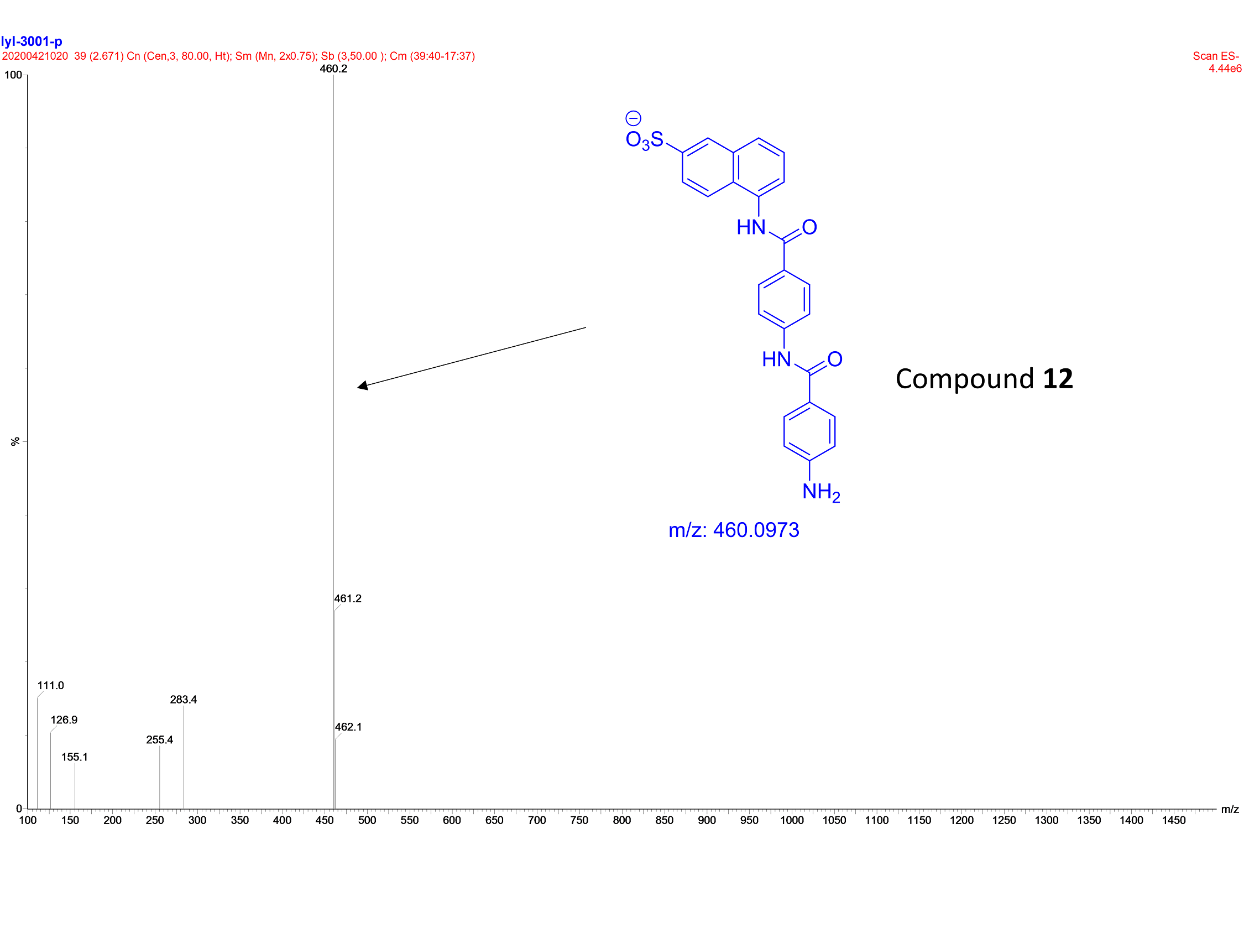


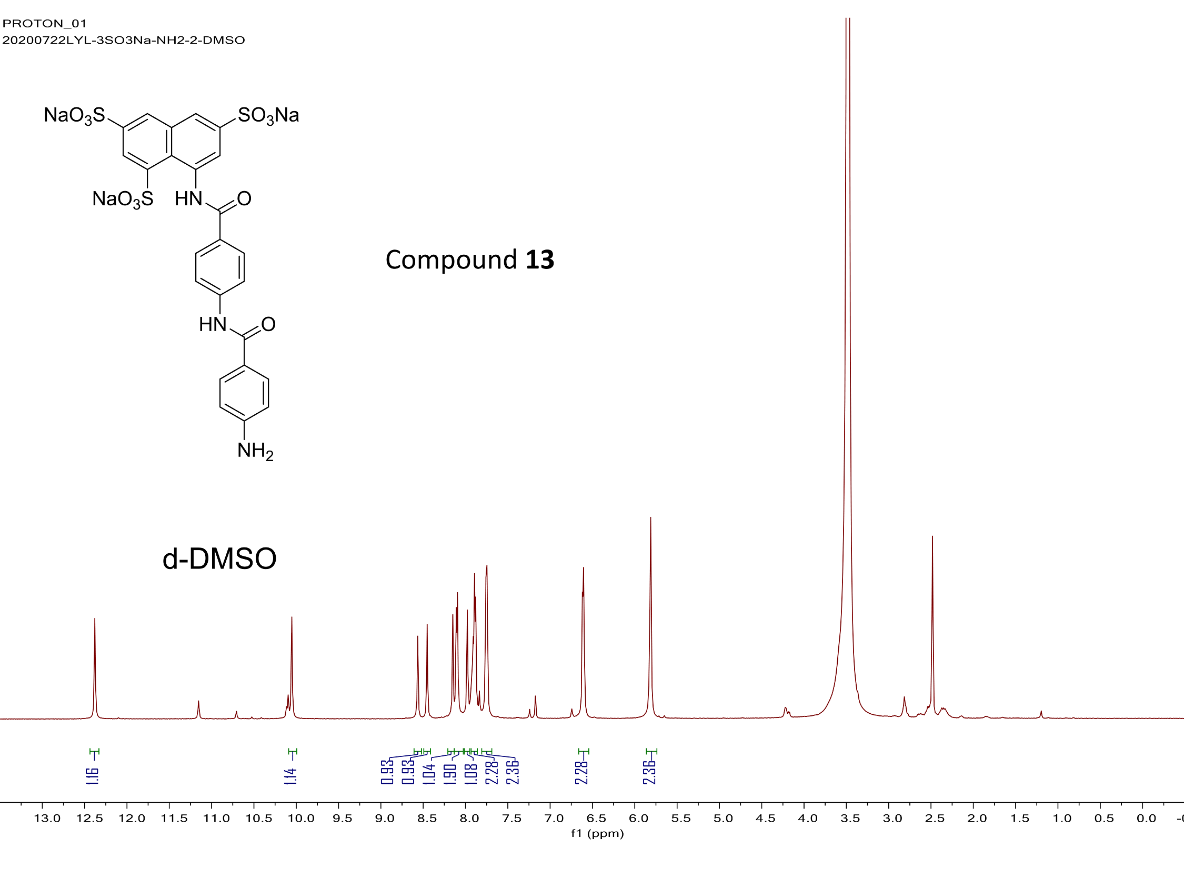


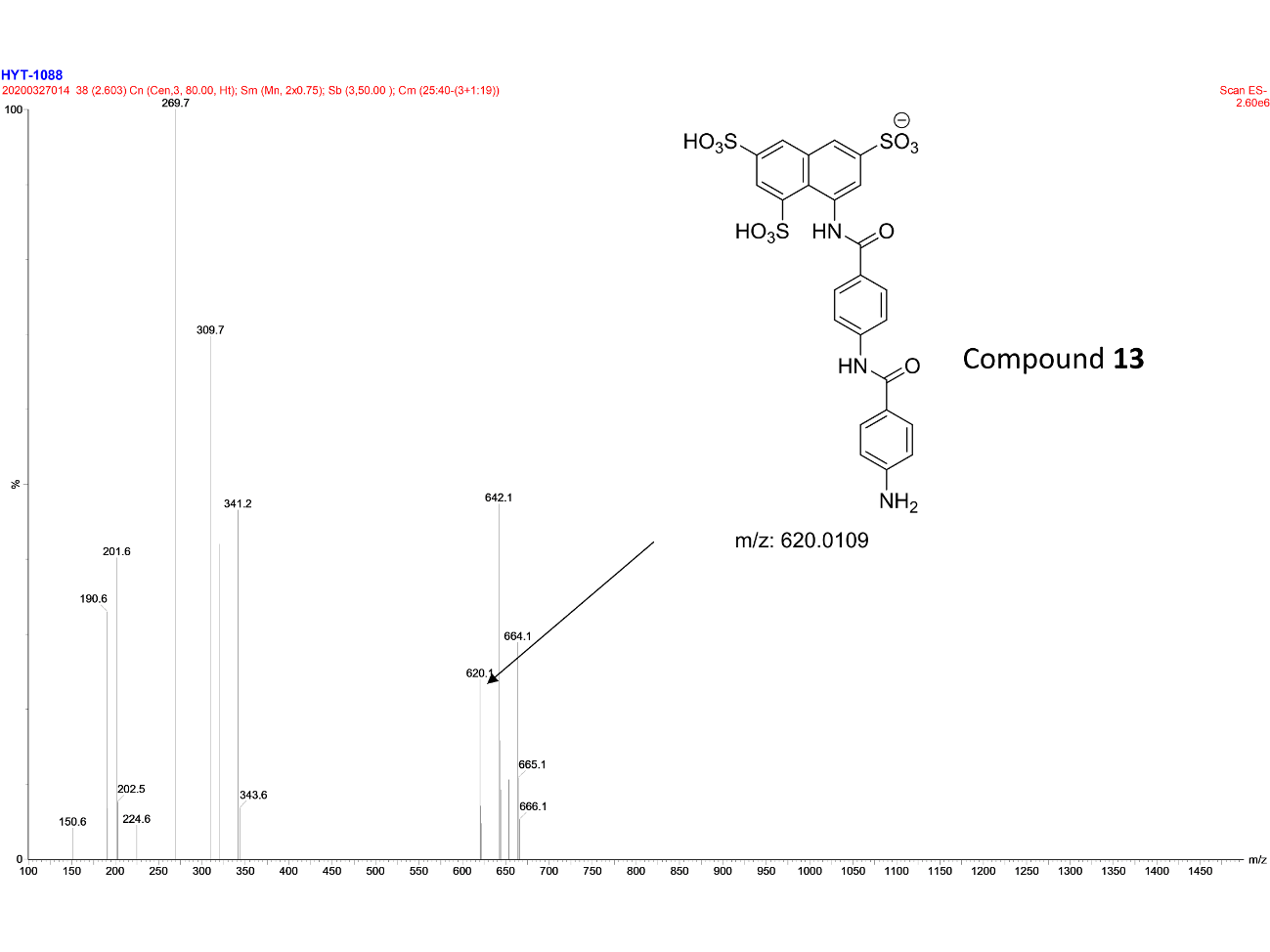


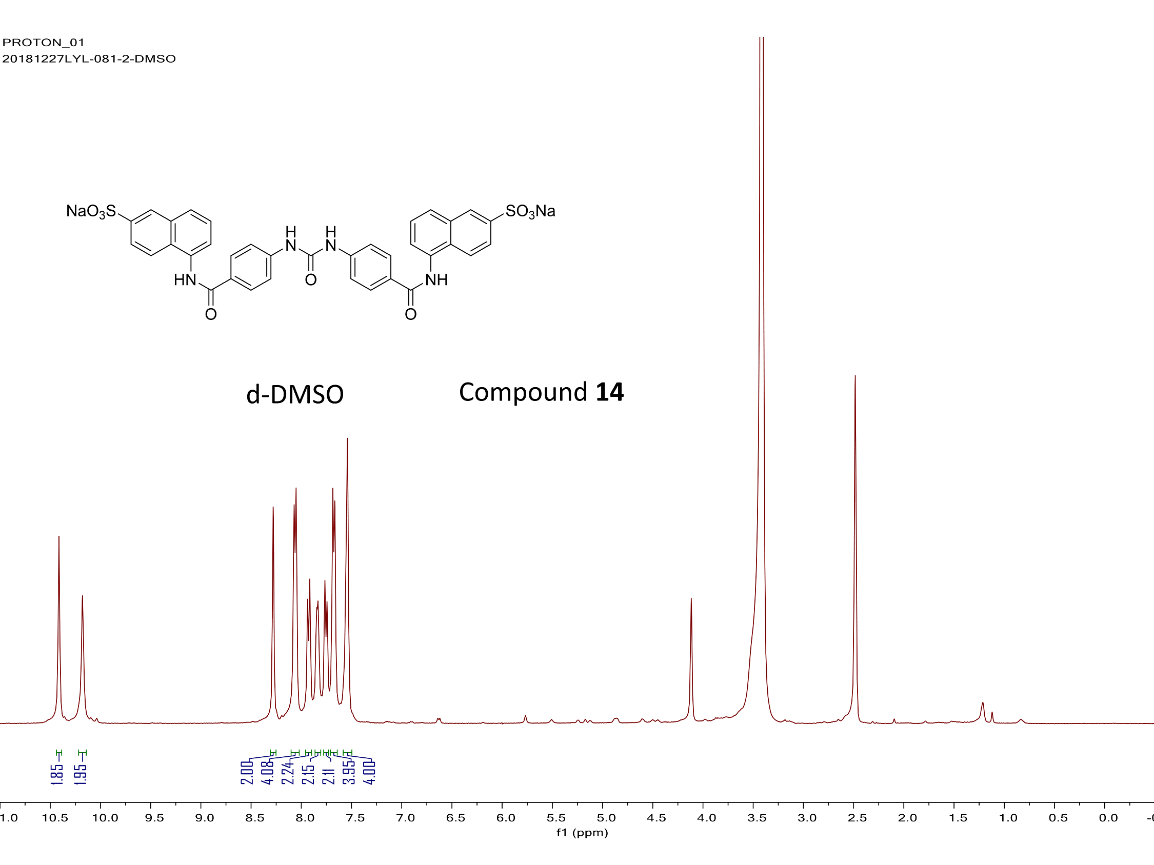


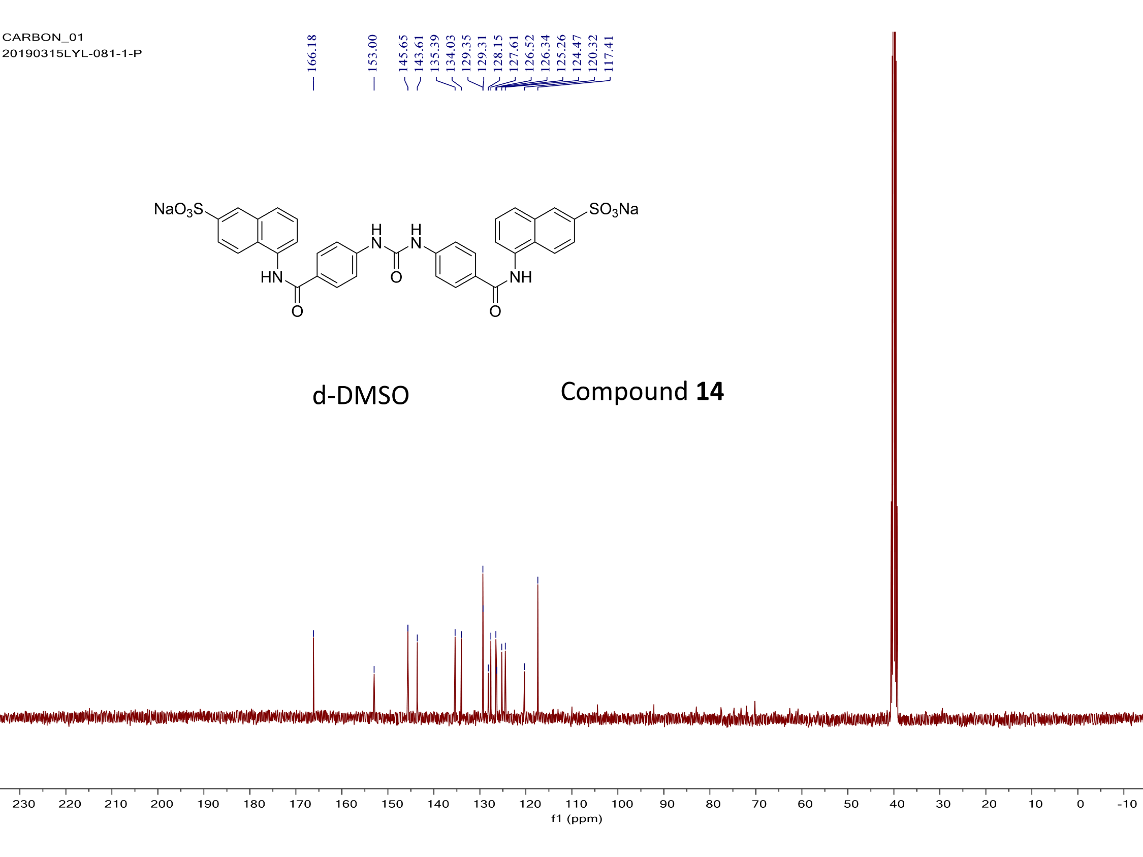


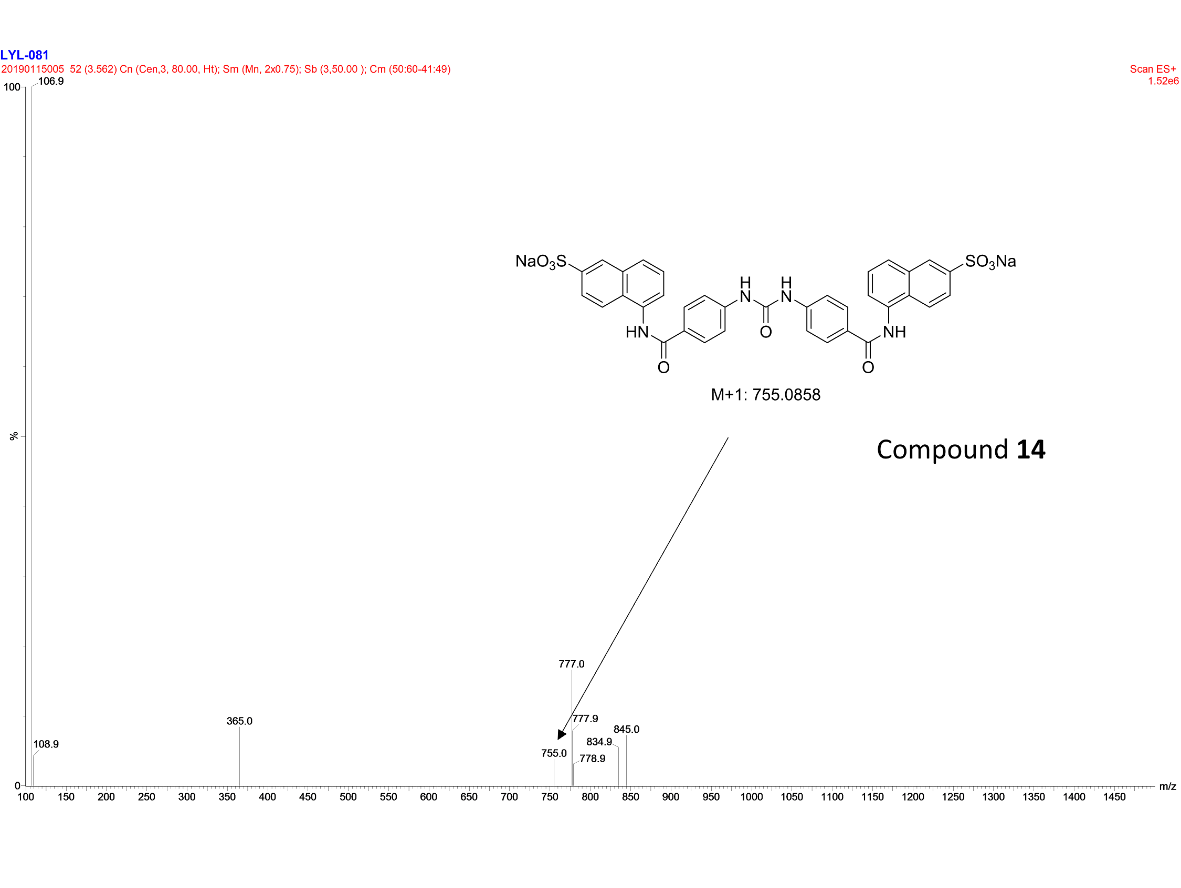


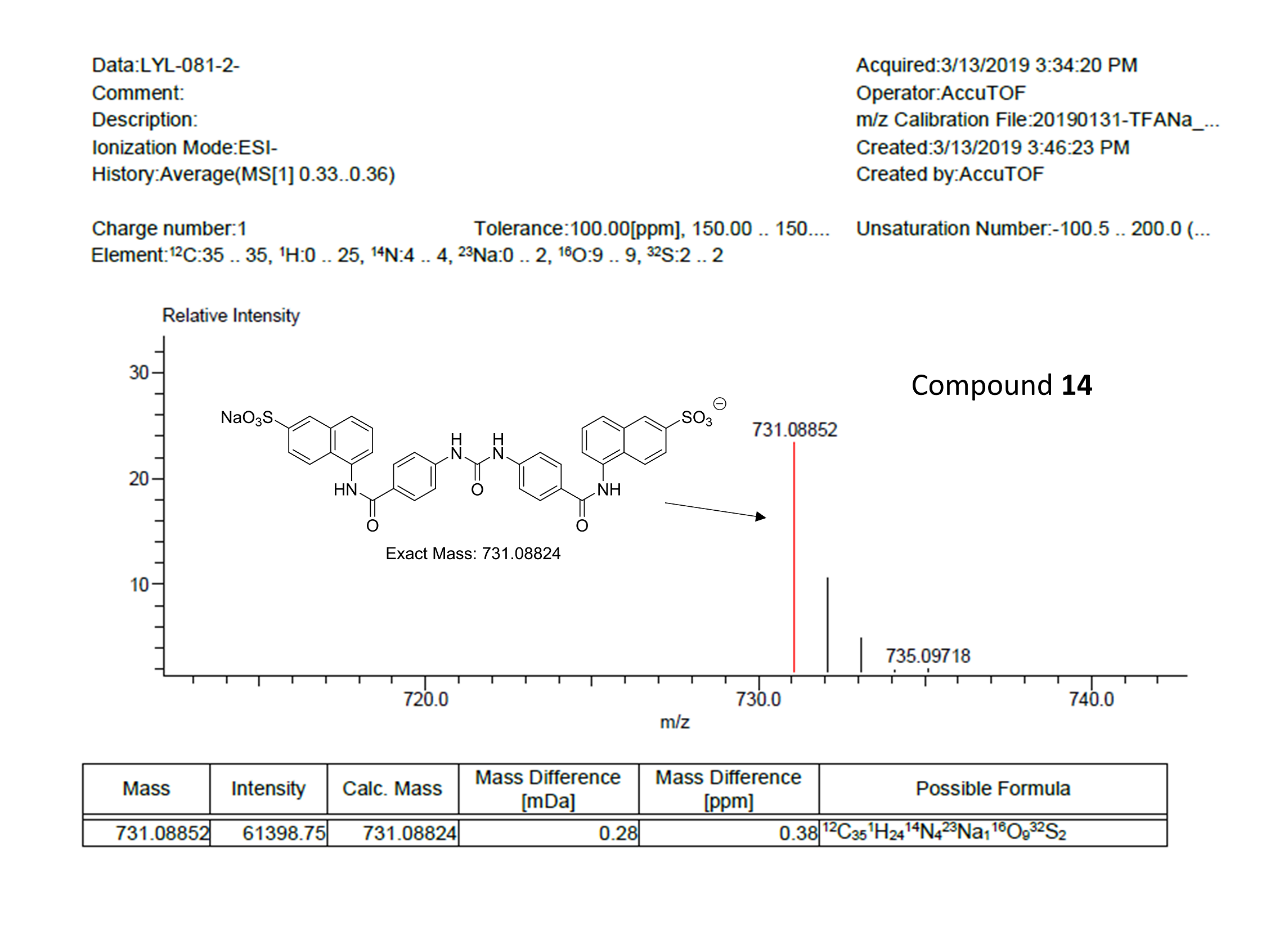


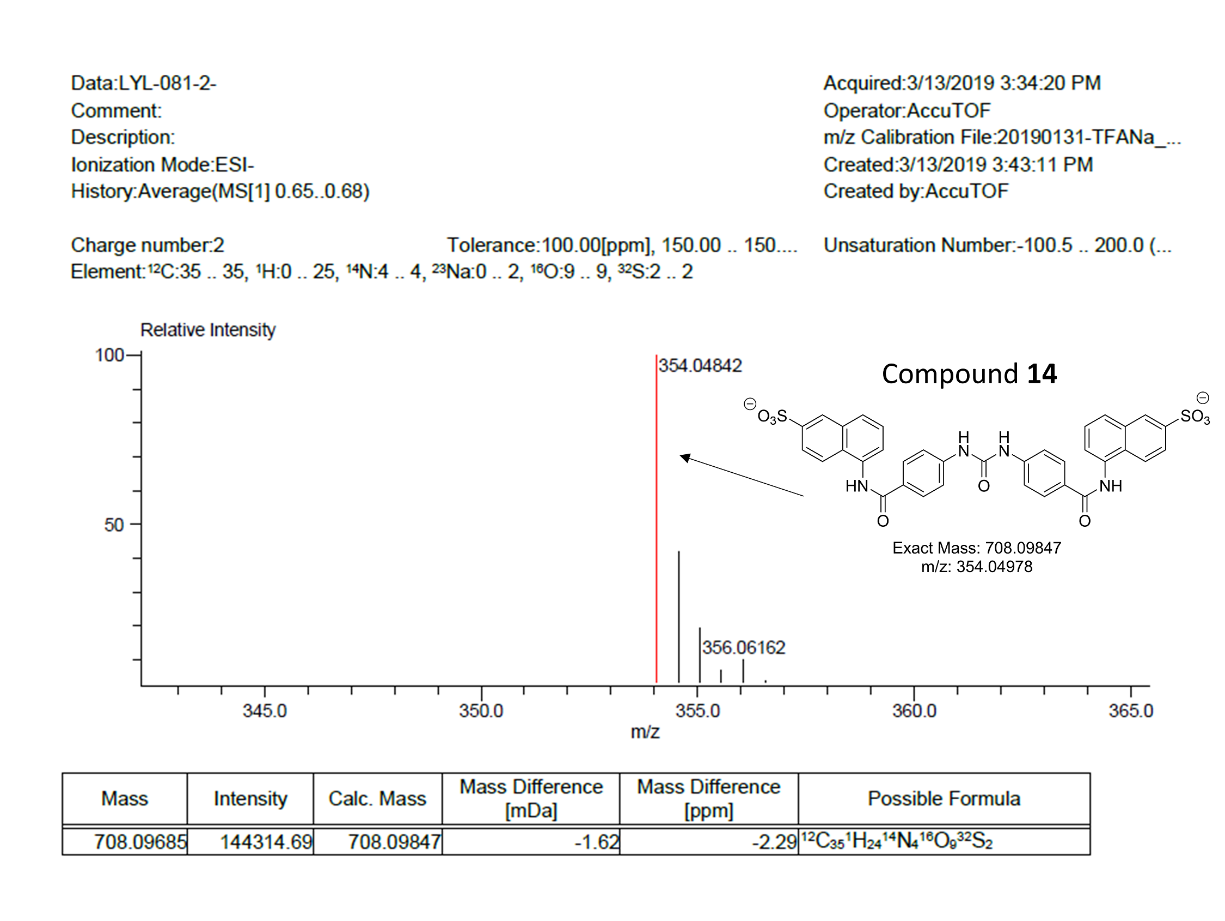


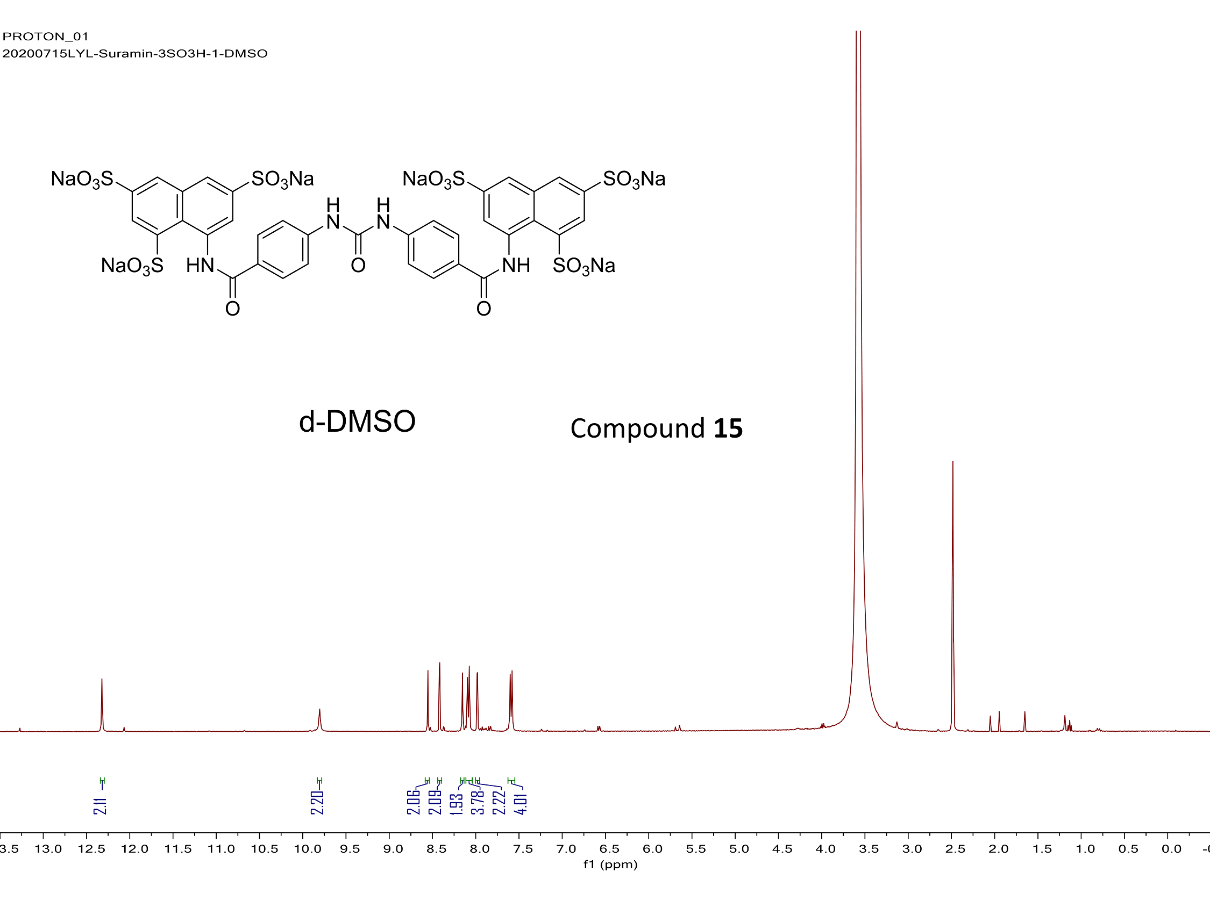


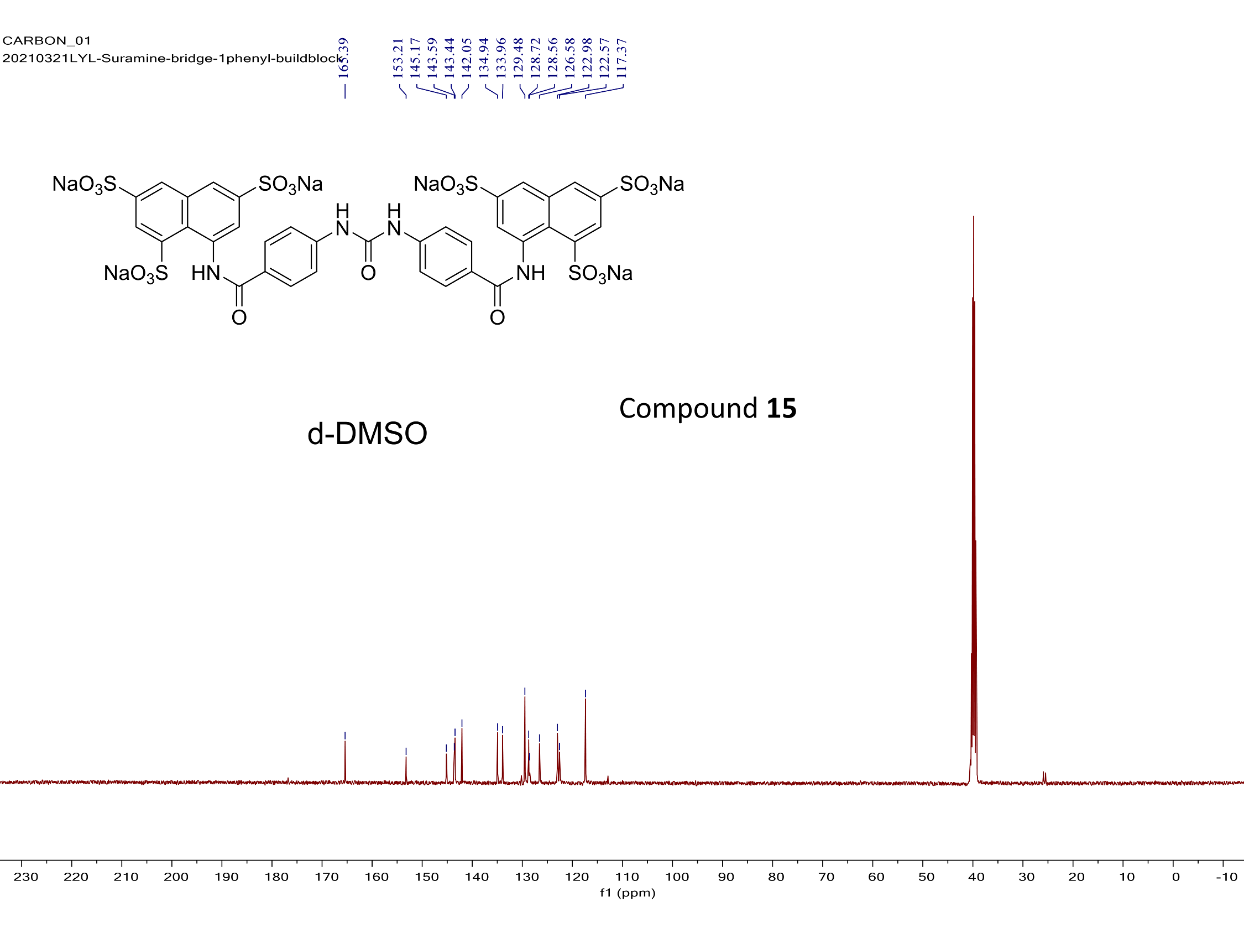


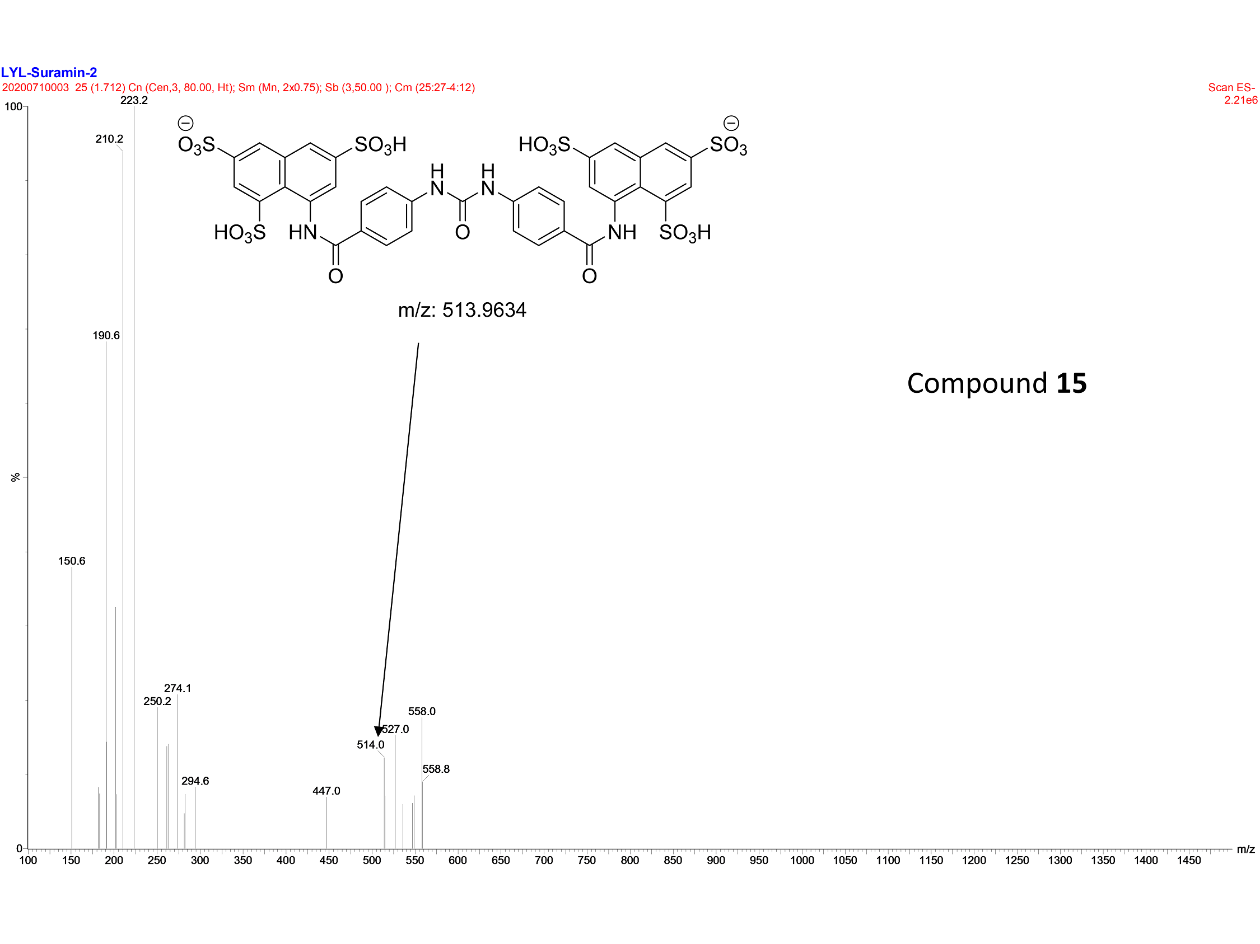


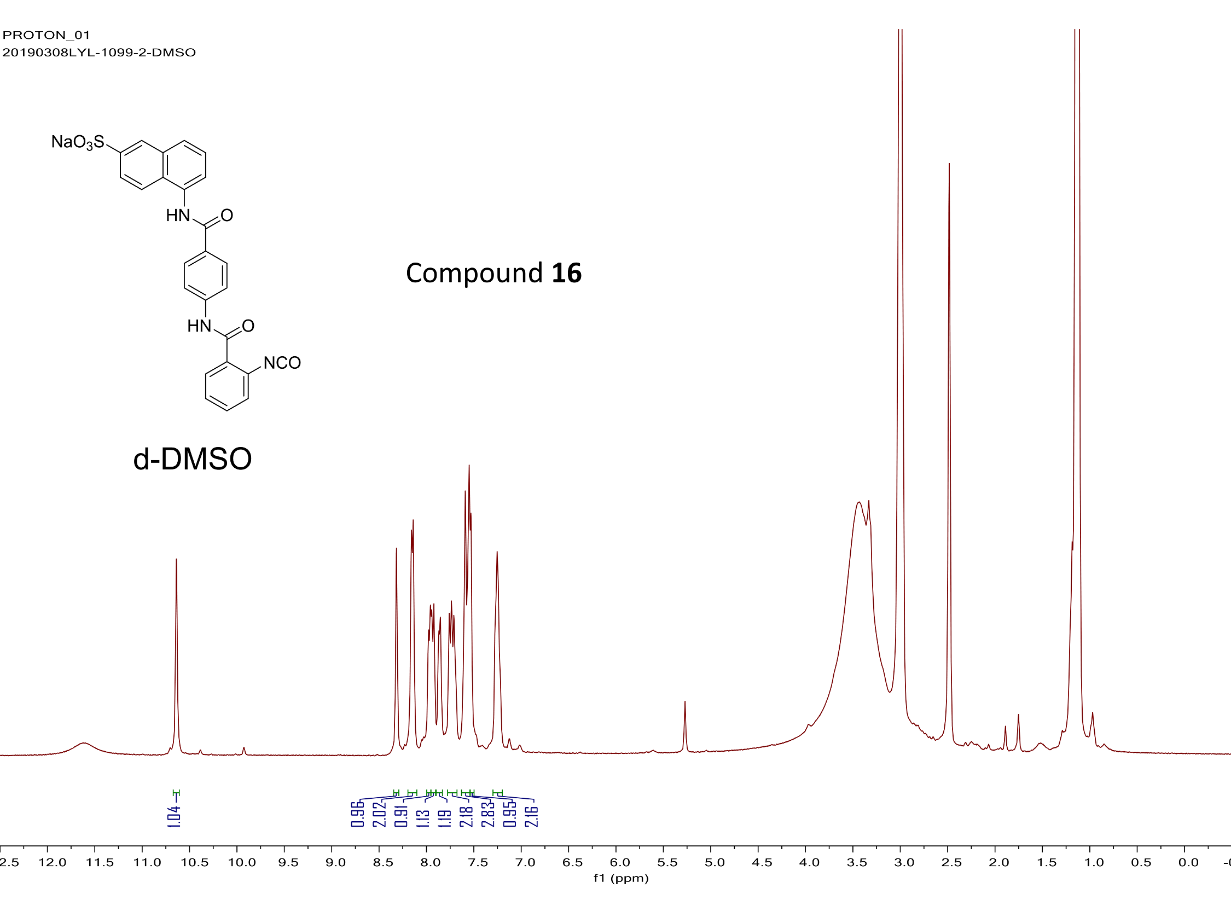


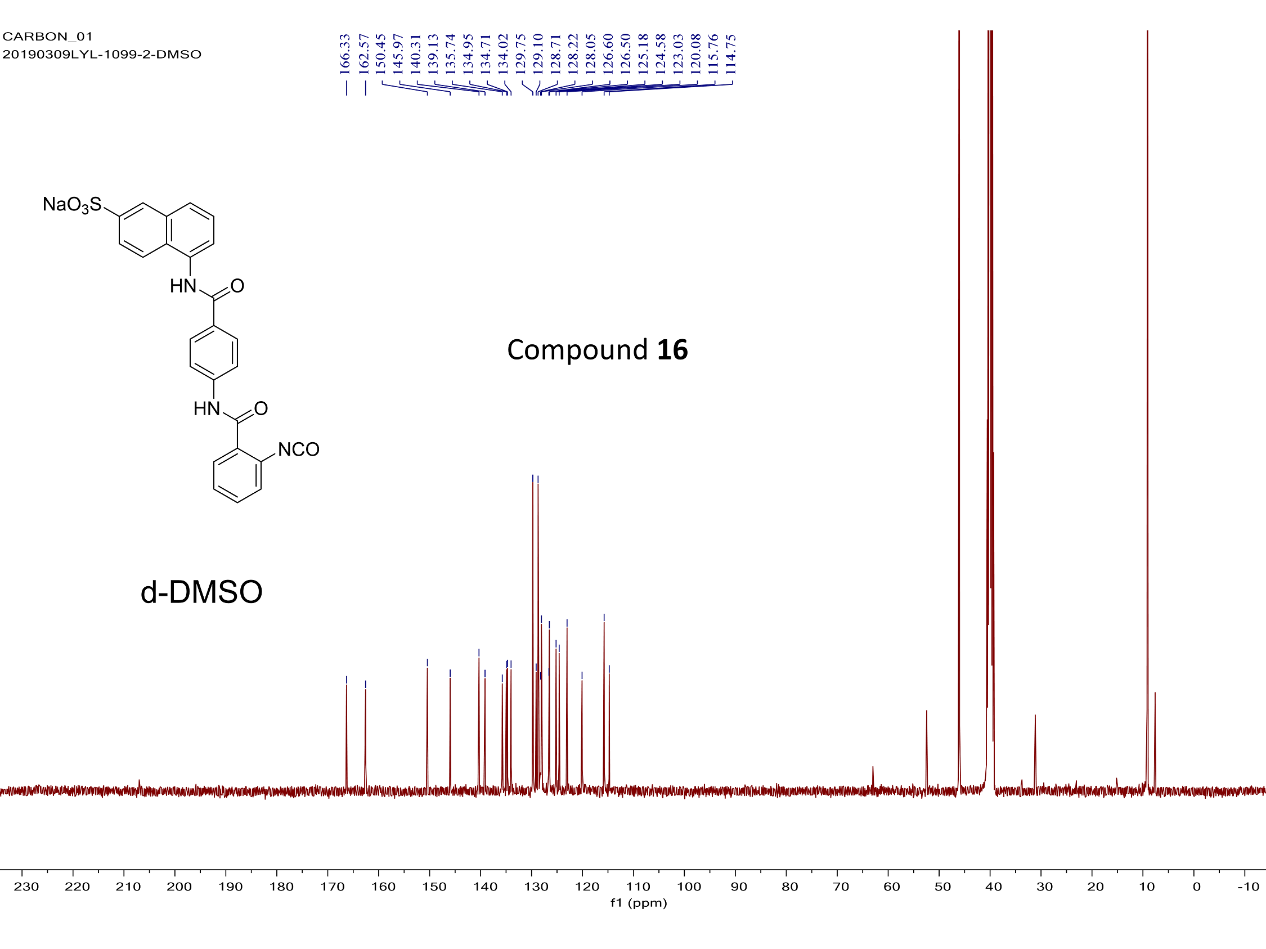


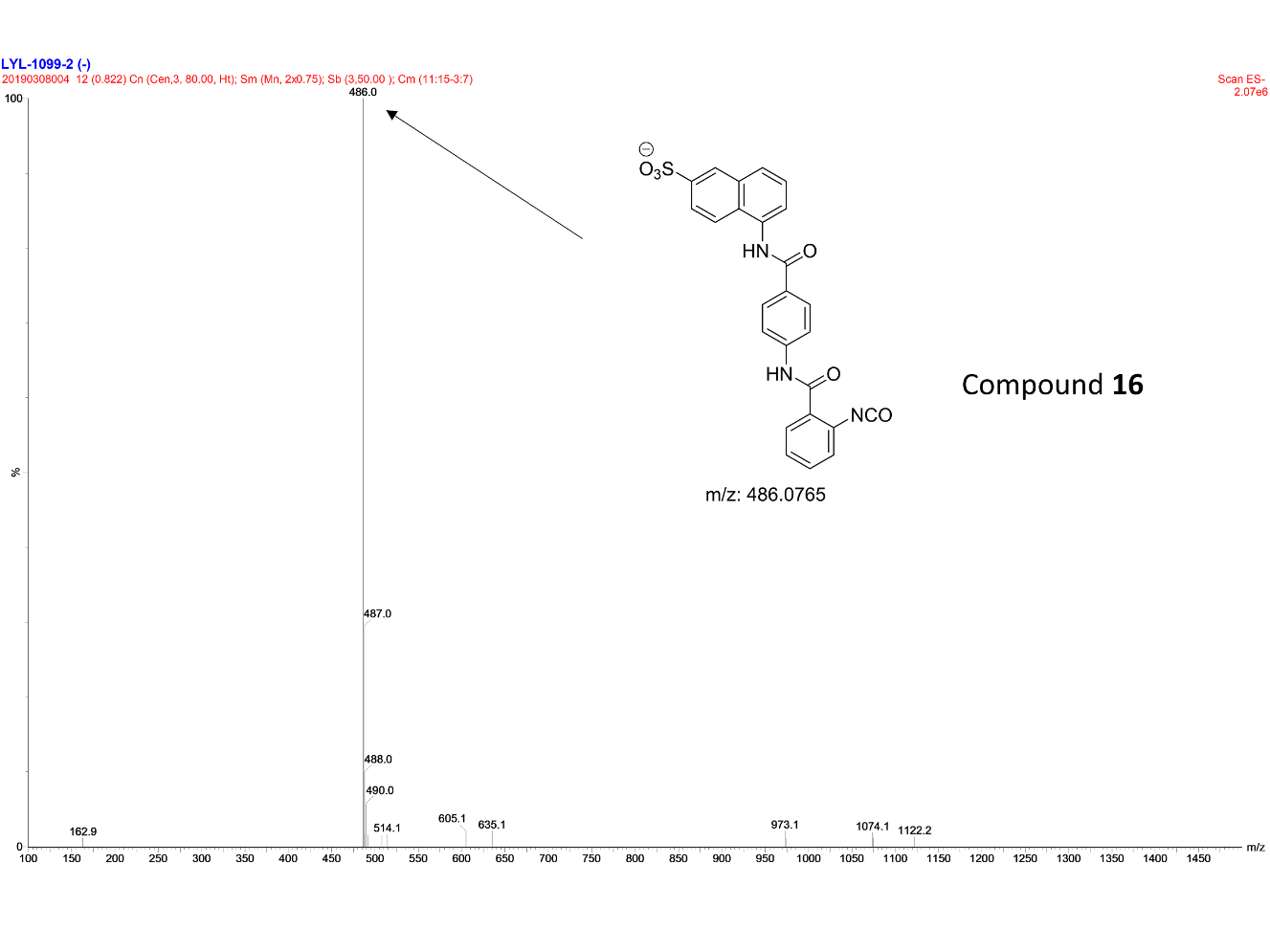


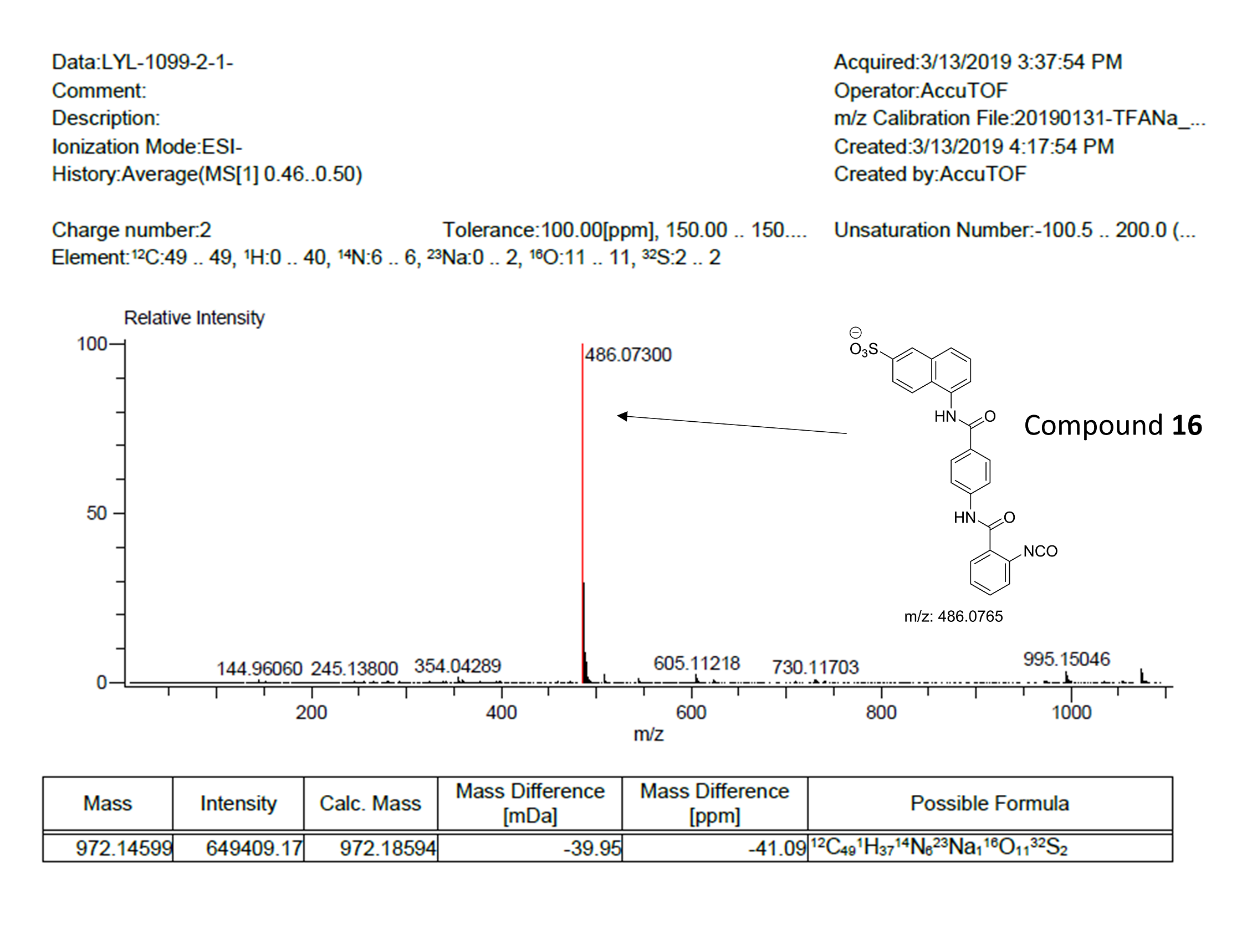


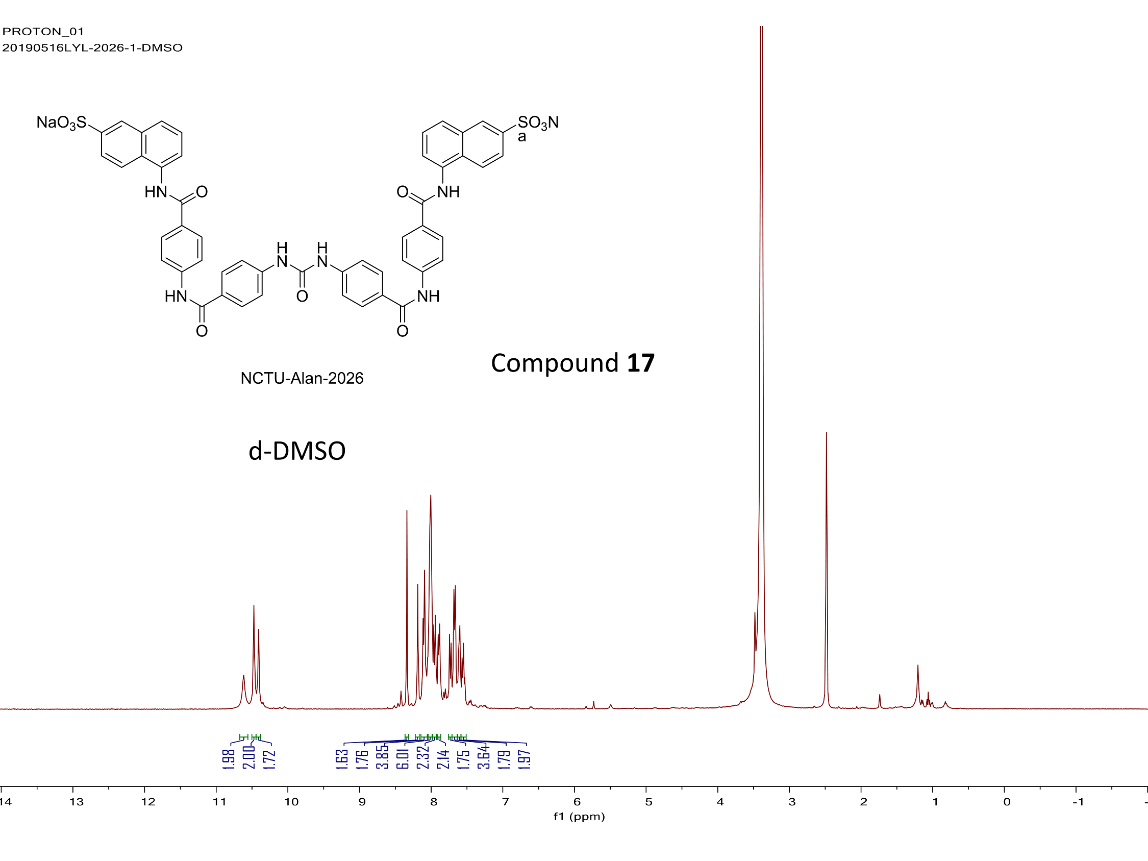


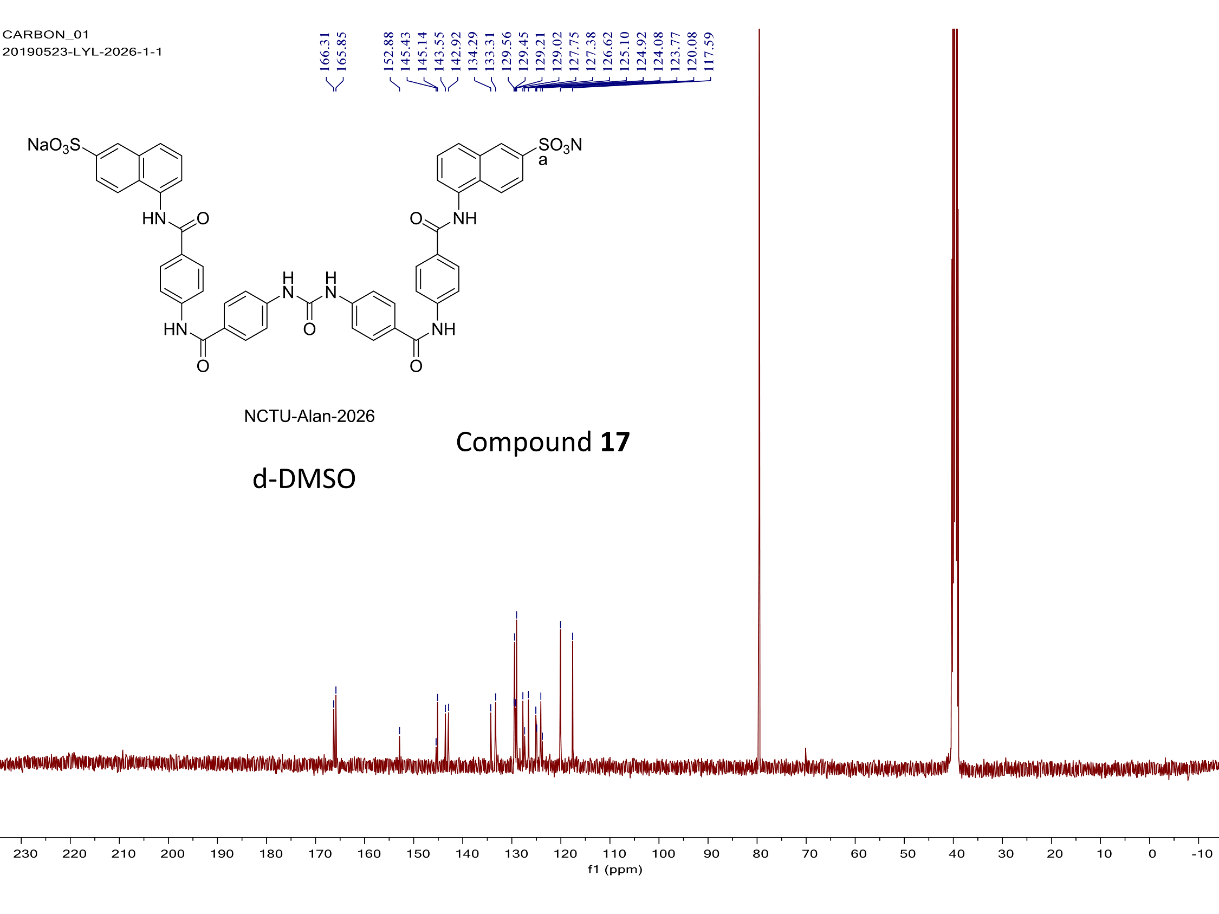


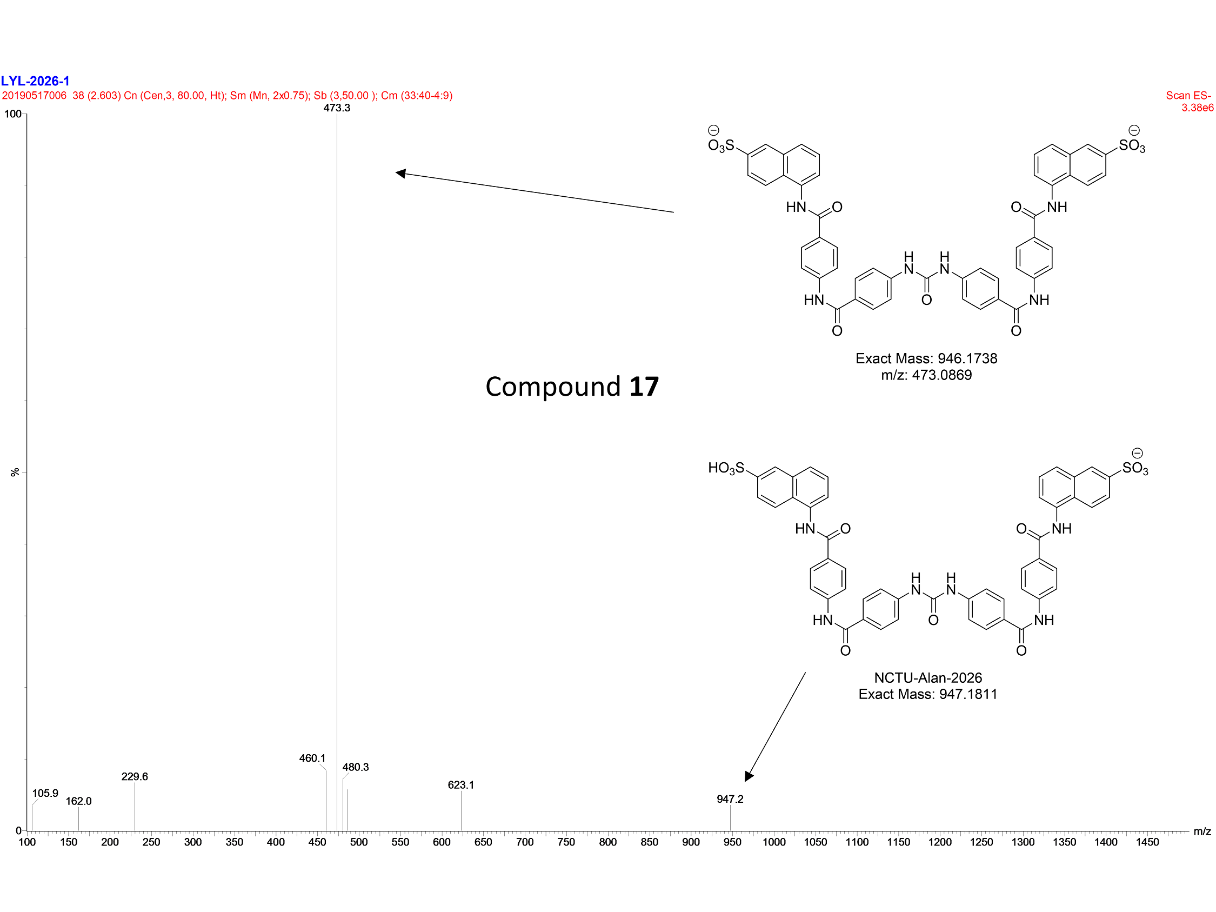


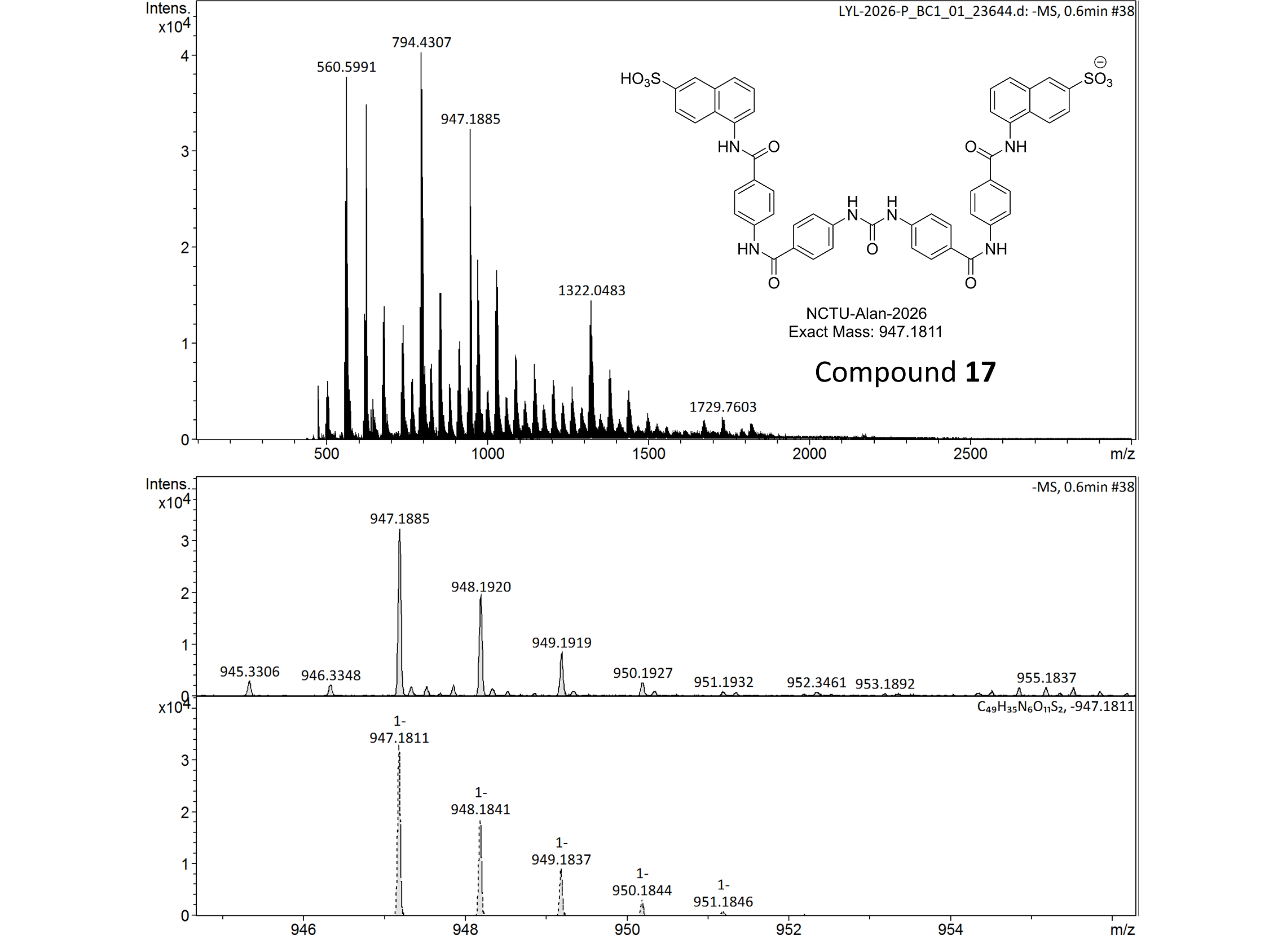


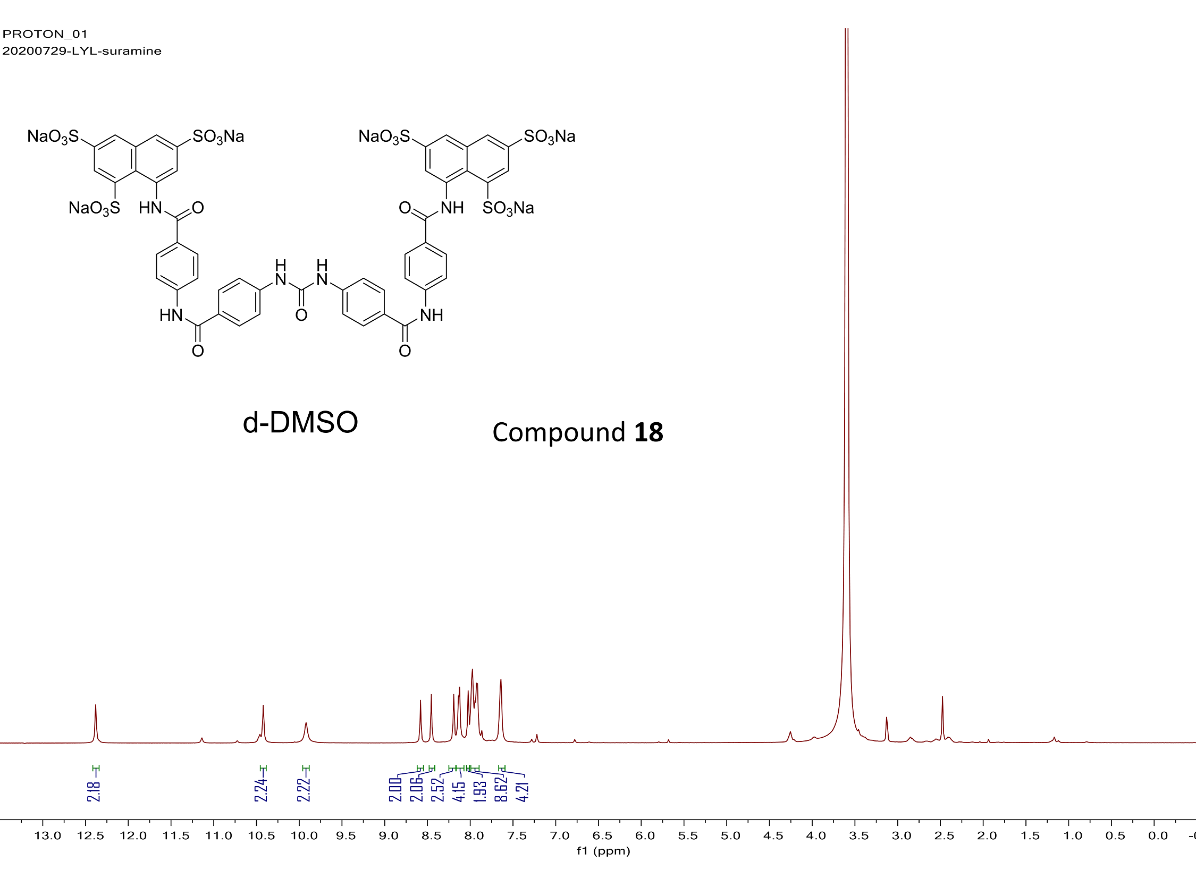


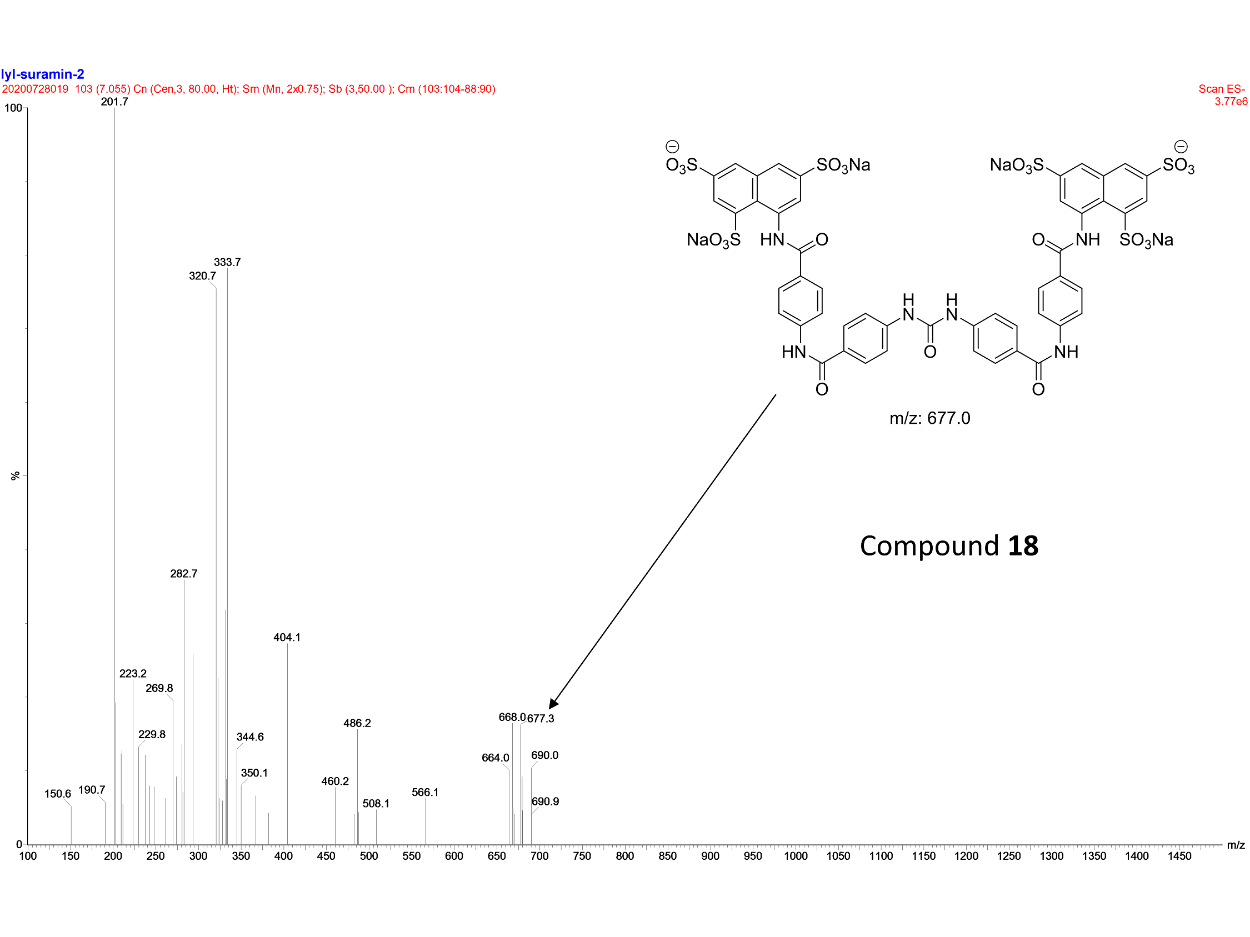


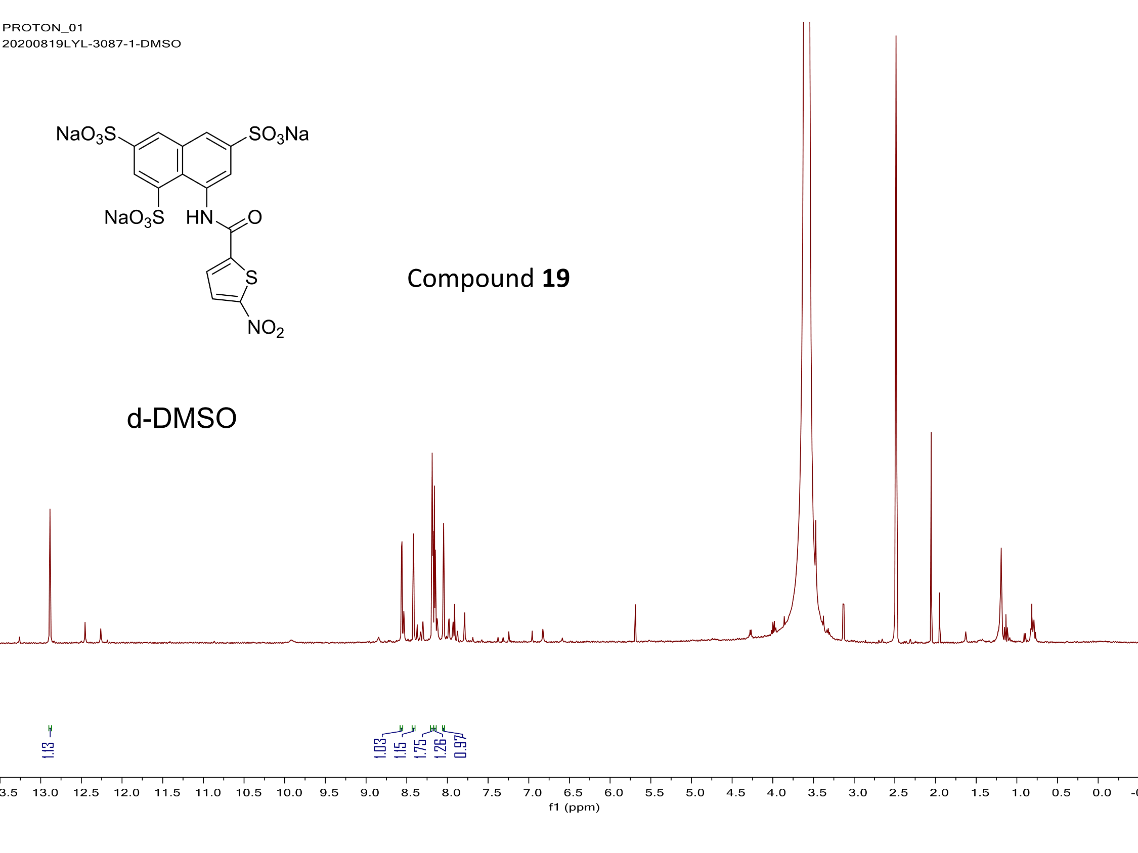


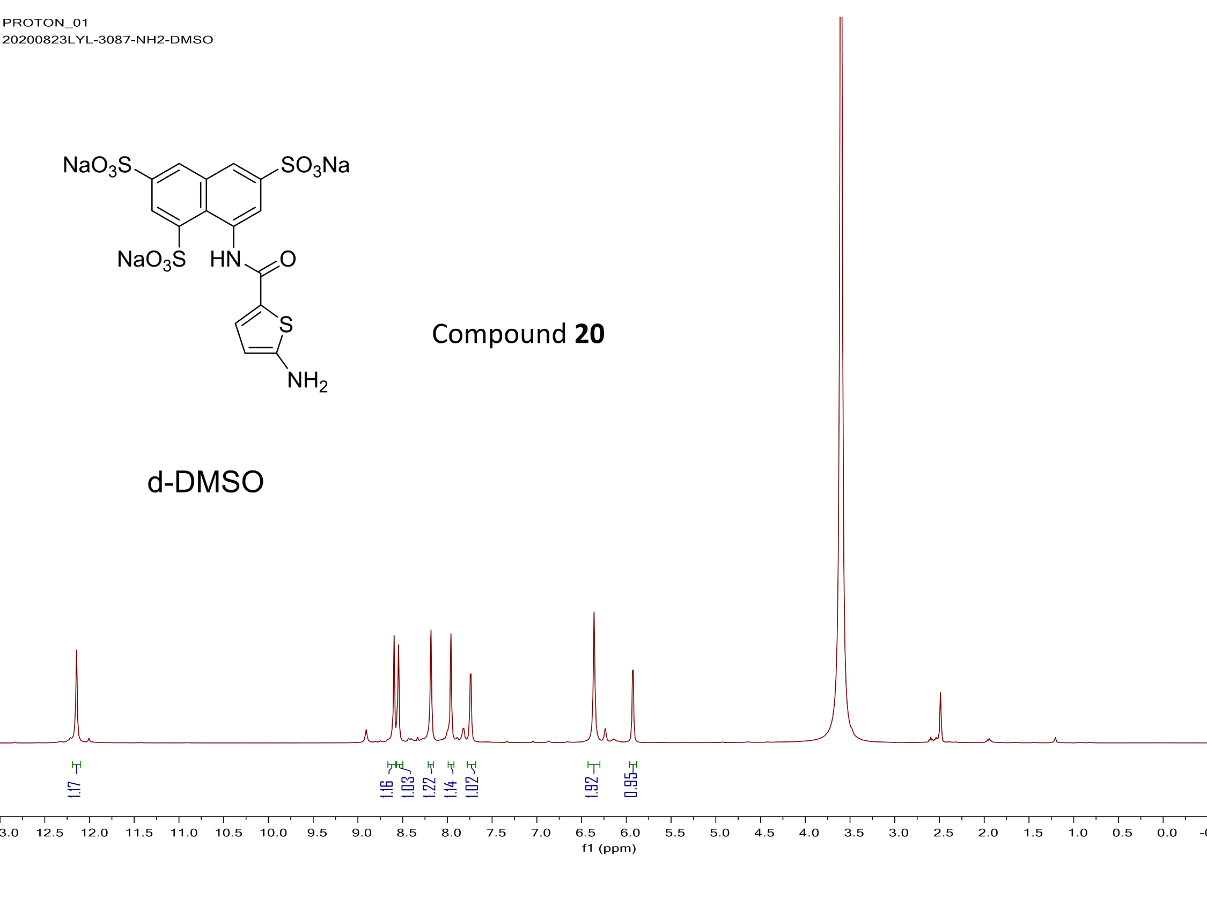


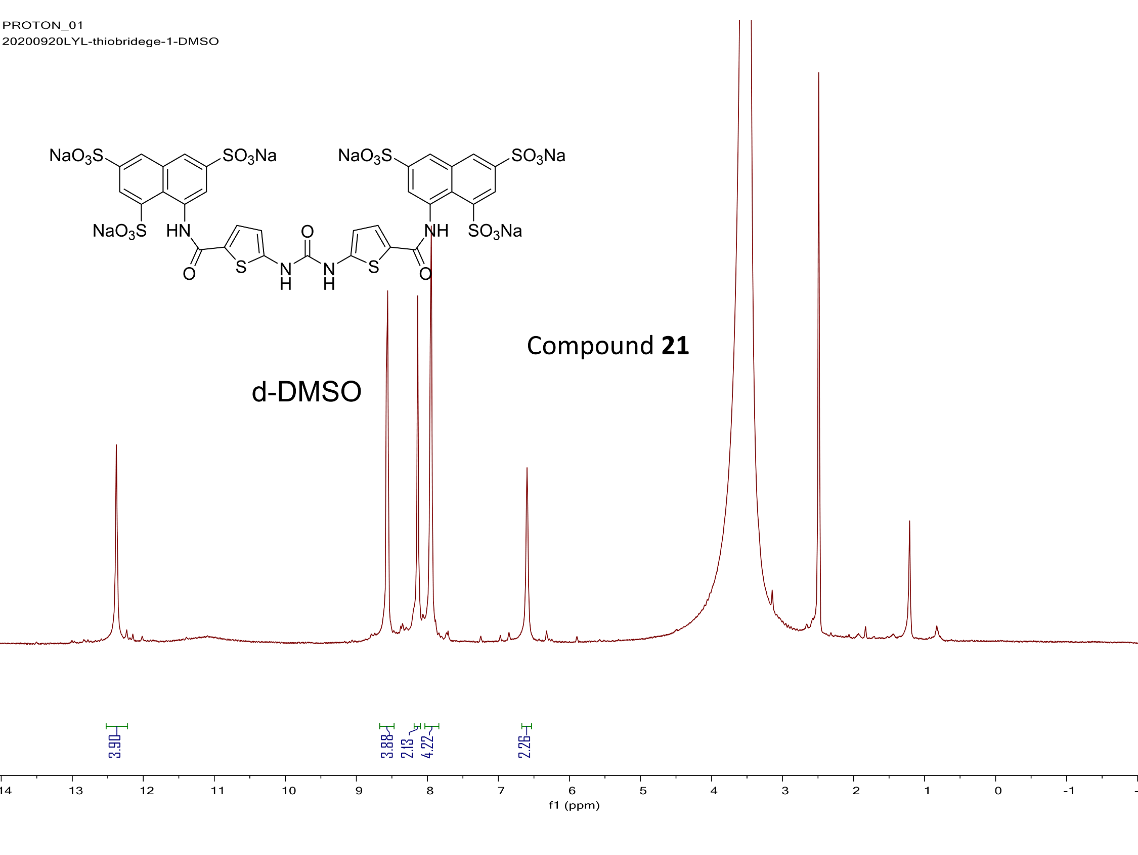


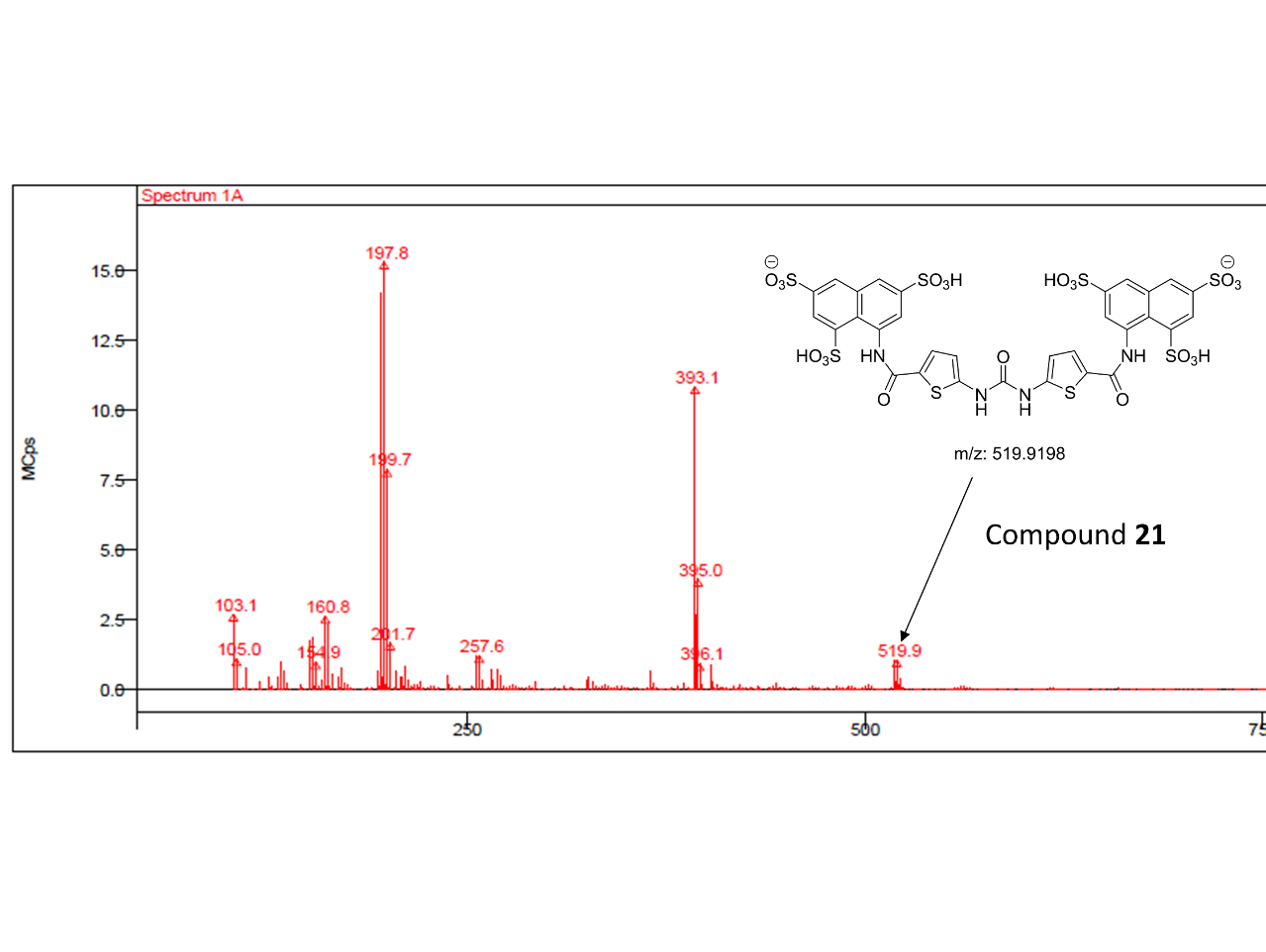


**S2. Stability of the compounds in aqueous solution and DMSO**

11 Days

1 Day

4 Days

**Figure S2-1.** ^1^H NMR spectrum of compound **15** in DMSO-*d_6_*.

1 Day

4 Days

11 Days

**Figure S2-2.** ^1^H NMR spectrum of compound **15** in D_2_O.

 **Figure S2-3.** ^1^H NMR spectrum of compound **18** in DMSO-*d_6_*.

11 Days

4 Days

1 Day

 **Figure S2-4.** ^1^H NMR spectrum of compound **18** in D_2_O.

1 Day

4 Days

11 Days

**S3. SDS PAGE of FGF1**


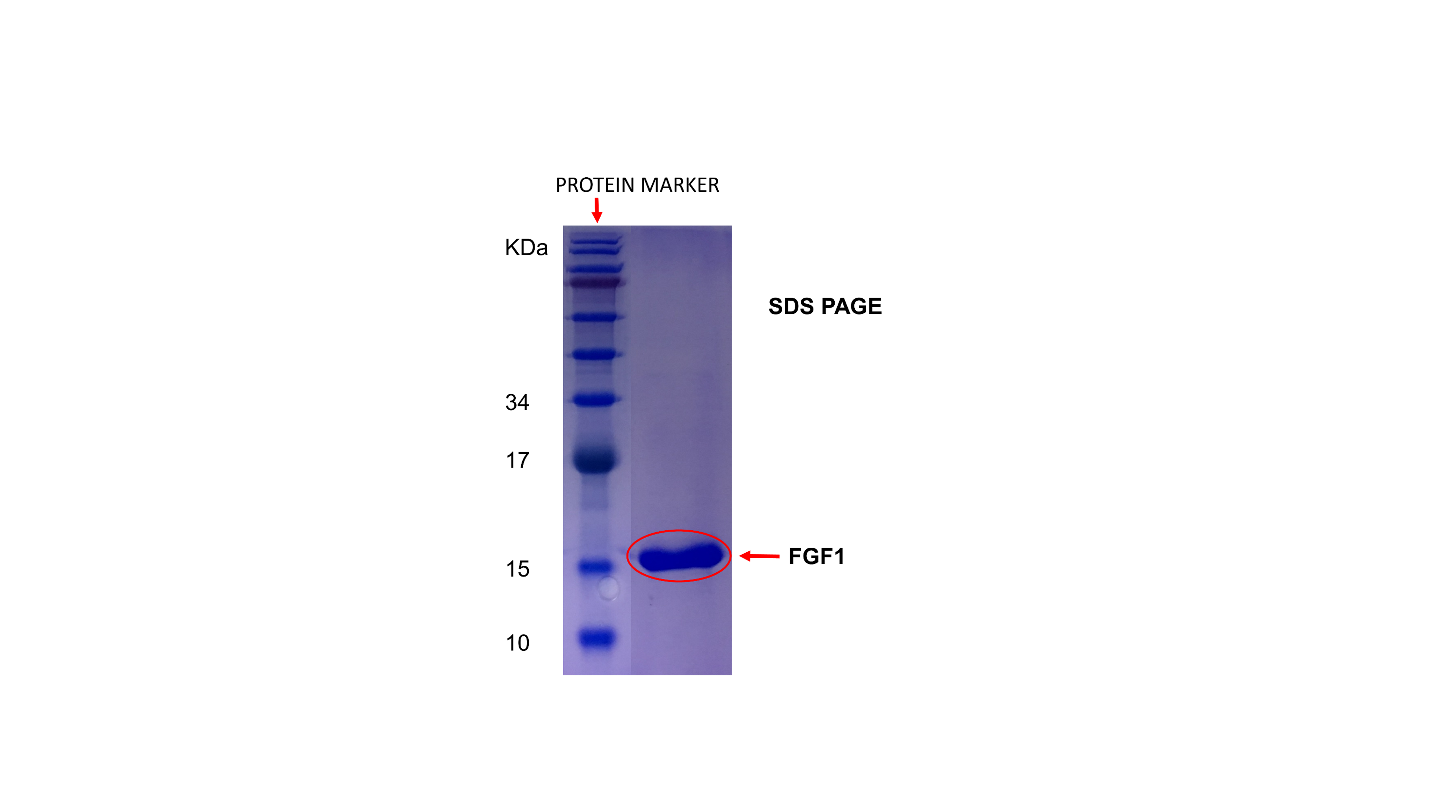


**S4. HPLC Details for FGF1**

**
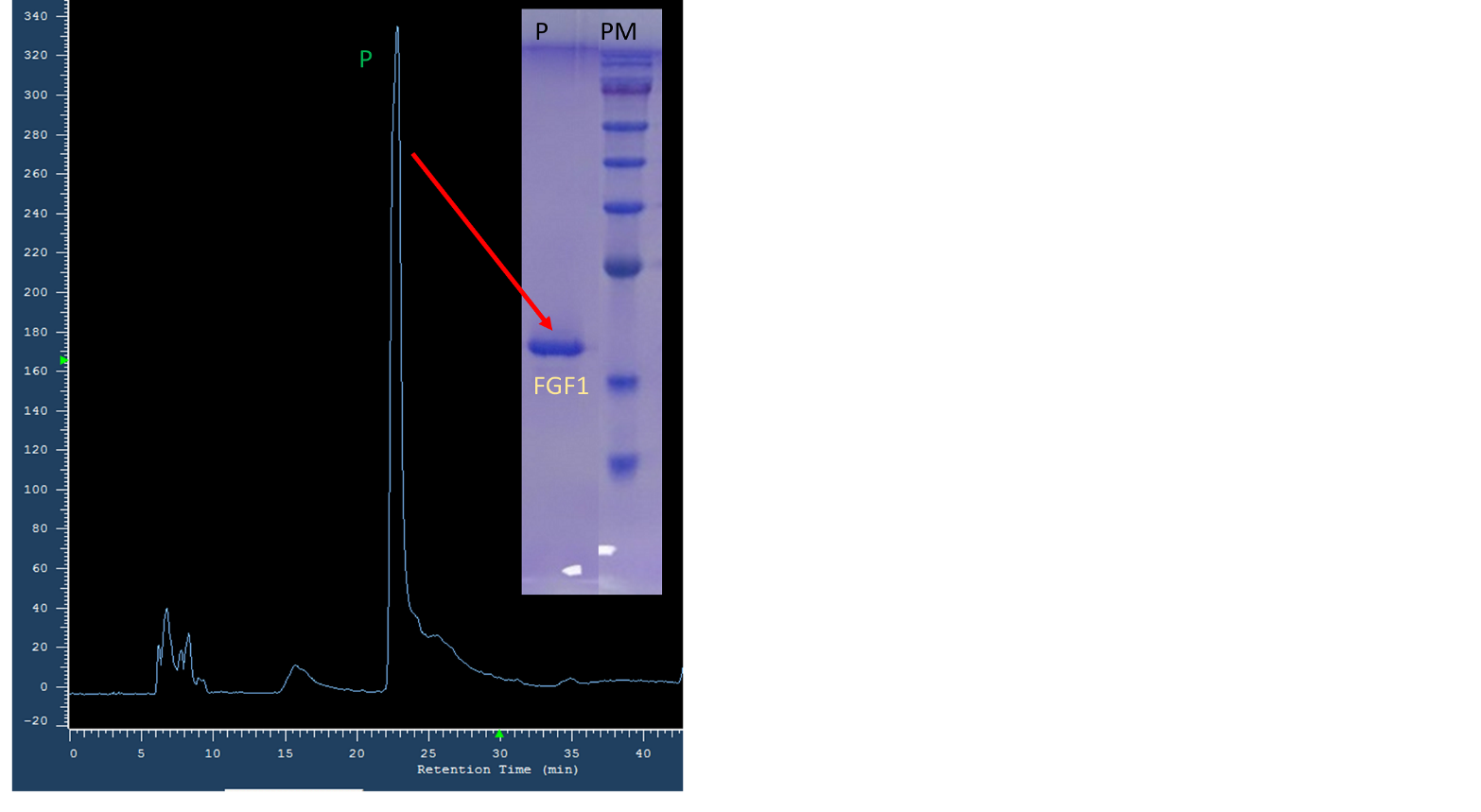
**

P: PEAK

PM: PROTEIN MARKER

**S5. ESI-MASS of FGF1**


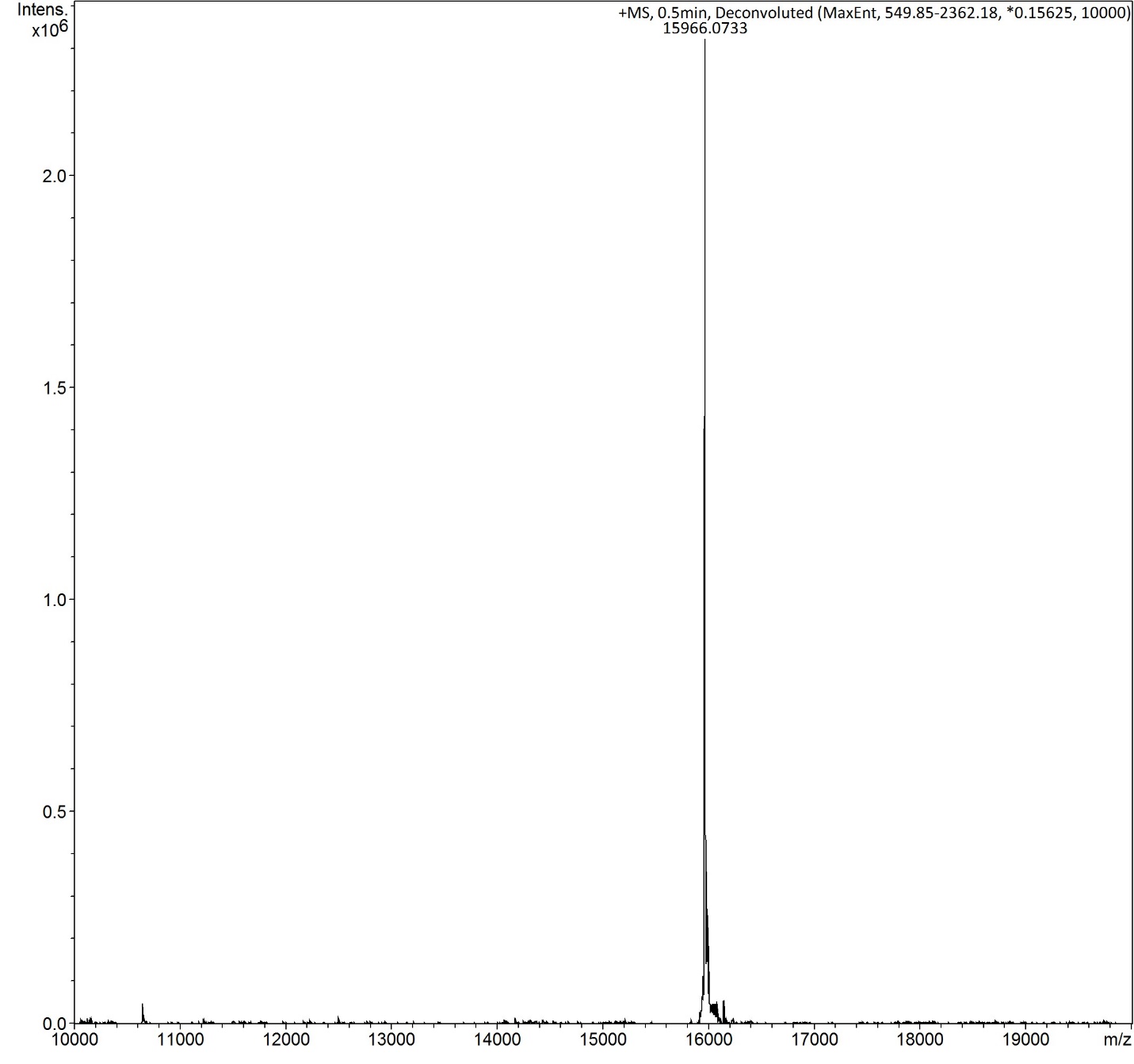


Theoretical MW: 15967.00

Experimental MW: 15966.0733

**Compound 14**

**S6. Validation of Docking protocol**


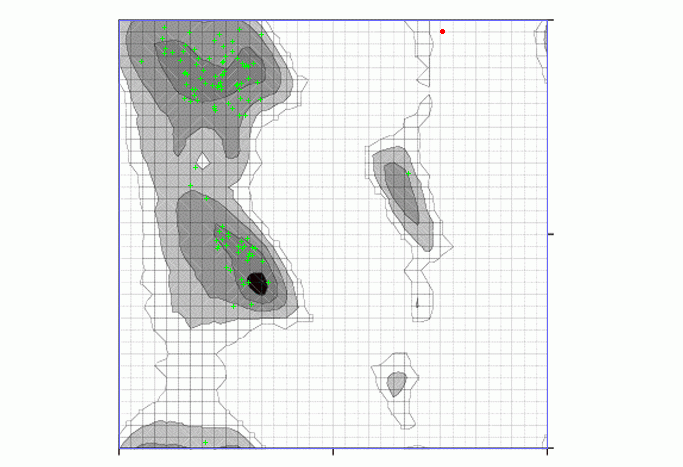


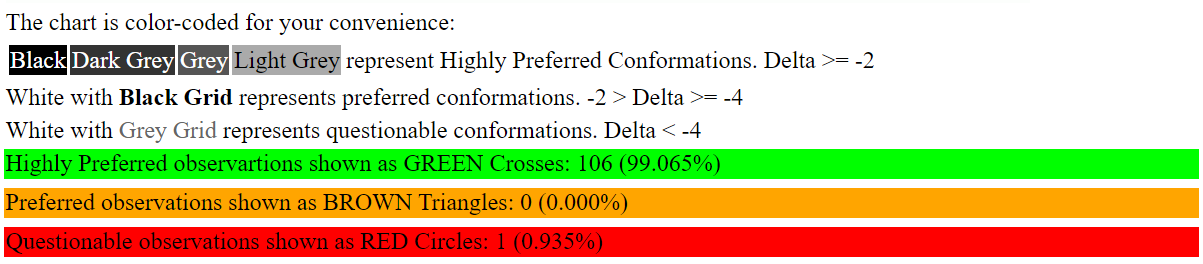


**Compound 15**


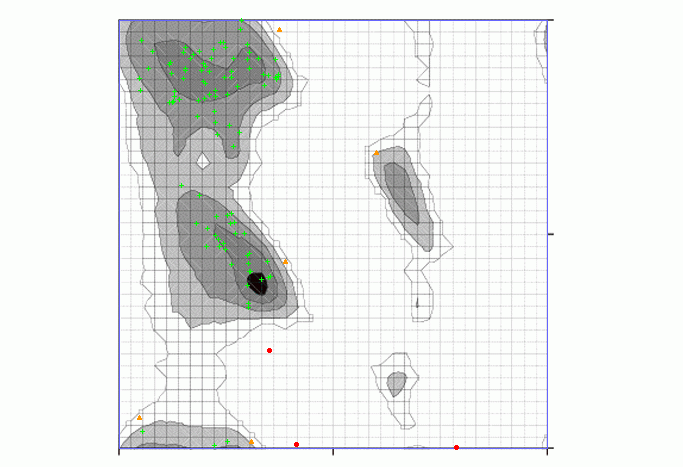


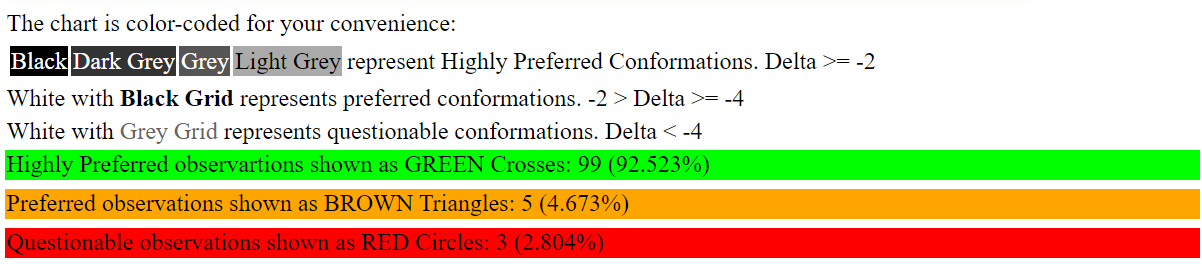


**Compound 17**


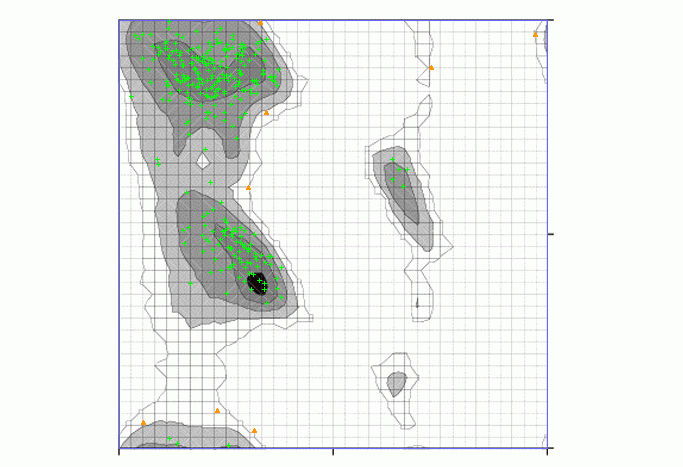


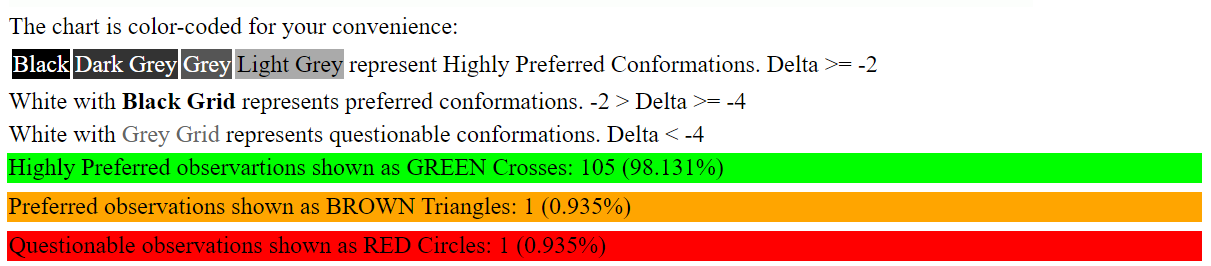

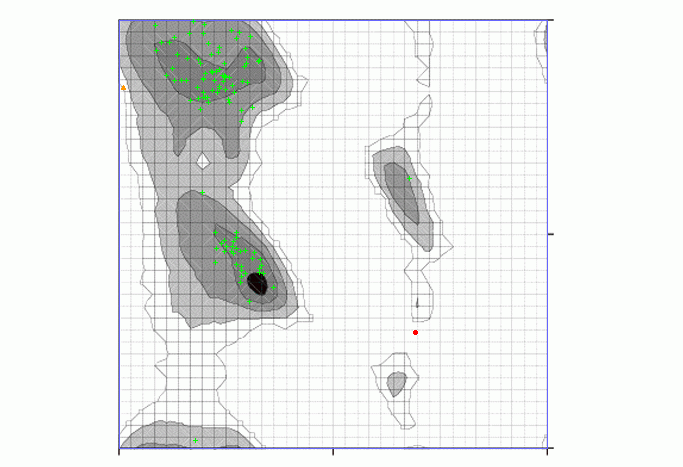

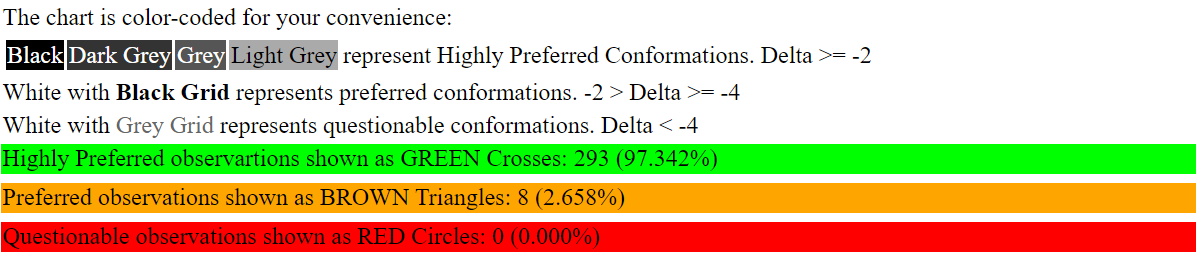


**Compound 18**


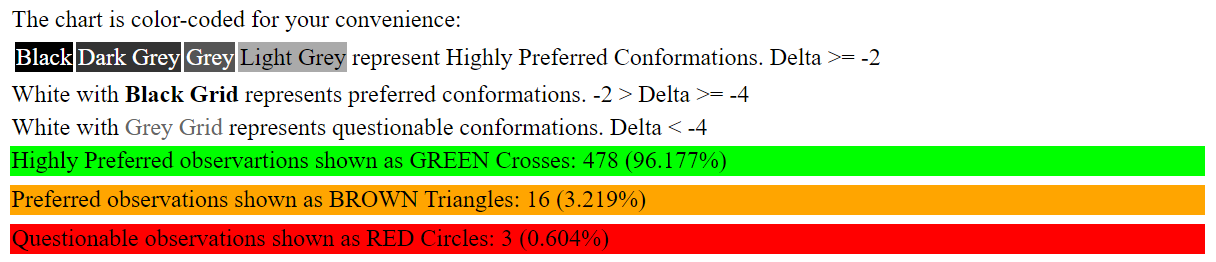

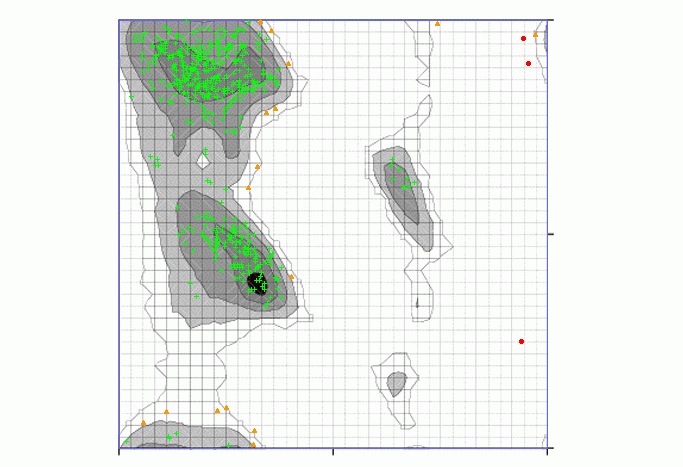


**Compound 21**

**S7. Cytotoxicity evaluated against normal breast cell line**


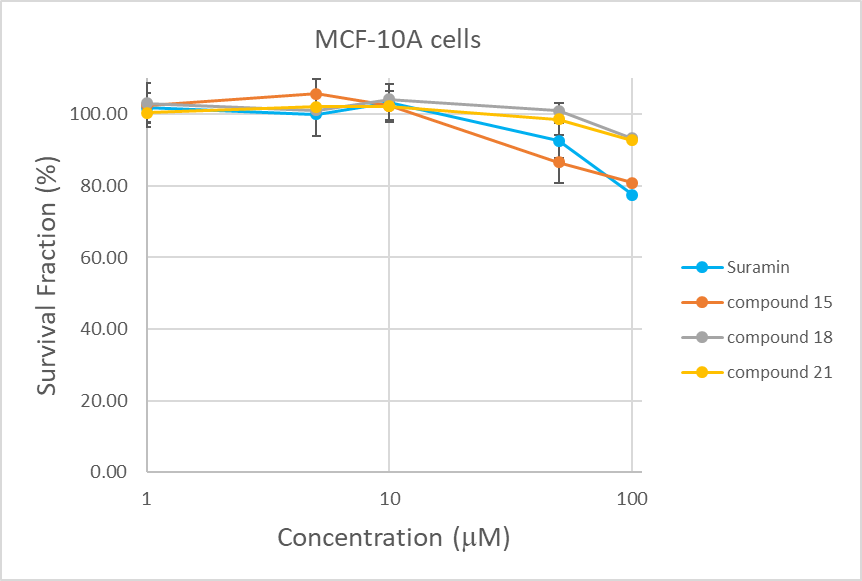


**S8. Statistical data for the cytotoxicity**

| **Name** | **Blank 450** | **Mean** | **Std Dev** | **CV (%)** | **Fold** | **Fold (mean)** | **SD (fold)** |
| --- | --- | --- | --- | --- | --- | --- | --- |
| Control | 1.175 | 1.165 | 0.011 | 1.0% | 1.008 | 1.00 | 0.01 |
|  | 1.17 |  |  |  | 1.004 |  |  |
|  | 1.167 |  |  |  | 1.002 |  |  |
|  | 1.149 |  |  |  | 0.986 |  |  |
| 1 uM suramin | 1.186 | 1.211 | 0.053 | 4.4% | 1.018 | 1.04 | 0.05 |
|  | 1.173 |  |  |  | 1.007 |  |  |
|  | 1.197 |  |  |  | 1.027 |  |  |
|  | 1.289 |  |  |  | 1.106 |  |  |
| 5 uM suramin | 1.137 | 1.160 | 0.038 | 3.3% | 0.976 | 1.00 | 0.03 |
|  | 1.217 |  |  |  | 1.044 |  |  |
|  | 1.147 |  |  |  | 0.984 |  |  |
|  | 1.138 |  |  |  | 0.977 |  |  |
| 10 uM suramin | 1.187 | 1.116 | 0.068 | 6.1% | 1.019 | 0.96 | 0.06 |
|  | 1.064 |  |  |  | 0.913 |  |  |
|  | 1.159 |  |  |  | 0.995 |  |  |
|  | 1.052 |  |  |  | 0.903 |  |  |
| 50 uM suramin | 0.936 | 1.004 | 0.063 | 6.3% | 0.803 | 0.86 | 0.05 |
|  | 0.988 |  |  |  | 0.848 |  |  |
|  | 1.088 |  |  |  | 0.934 |  |  |
|  | 1.004 |  |  |  | 0.862 |  |  |
| 100 uM suramin | 0.795 | 0.817 | 0.018 | 2.2% | 0.682 | 0.70 | 0.02 |
|  | 0.82 |  |  |  | 0.704 |  |  |
|  | 0.838 |  |  |  | 0.719 |  |  |
|  | 0.816 |  |  |  | 0.700 |  |  |
| 1 uM Compound 15 | 1.143 | 1.163 | 0.023 | 2.0% | 0.981 | 1.00 | 0.02 |
|  | 1.162 |  |  |  | 0.997 |  |  |
|  | 1.195 |  |  |  | 1.026 |  |  |
|  | 1.152 |  |  |  | 0.989 |  |  |
| 5 uM Compound 15 | 1.161 | 1.146 | 0.047 | 4.1% | 0.996 | 0.98 | 0.04 |
|  | 1.116 |  |  |  | 0.958 |  |  |
|  | 1.206 |  |  |  | 1.035 |  |  |
|  | 1.101 |  |  |  | 0.945 |  |  |
| 10 uM Compound 15 | 1.151 | 1.122 | 0.046 | 4.1% | 0.988 | 0.96 | 0.04 |
|  | 1.117 |  |  |  | 0.959 |  |  |
|  | 1.059 |  |  |  | 0.909 |  |  |
|  | 1.162 |  |  |  | 0.997 |  |  |
| 50 uM Compound 15 | 0.998 | 0.982 | 0.028 | 2.8% | 0.856 | 0.84 | 0.02 |
|  | 1.012 |  |  |  | 0.868 |  |  |
|  | 0.955 |  |  |  | 0.820 |  |  |
|  | 0.961 |  |  |  | 0.825 |  |  |
| 100 uM Compound 15 | 0.927 | 0.880 | 0.039 | 4.4% | 0.796 | 0.75 | 0.03 |
|  | 0.887 |  |  |  | 0.761 |  |  |
|  | 0.833 |  |  |  | 0.715 |  |  |
|  | 0.872 |  |  |  | 0.748 |  |  |
| 1 uM Compound 18 | 1.234 | 1.224 | 0.031 | 2.5% | 1.059 | 1.05 | 0.03 |
|  | 1.224 |  |  |  | 1.050 |  |  |
|  | 1.182 |  |  |  | 1.014 |  |  |
|  | 1.256 |  |  |  | 1.078 |  |  |
| 5 uM Compound 18 | 1.176 | 1.176 | 0.030 | 2.5% | 1.009 | 1.01 | 0.03 |
|  | 1.214 |  |  |  | 1.042 |  |  |
|  | 1.141 |  |  |  | 0.979 |  |  |
|  | 1.173 |  |  |  | 1.007 |  |  |
| 10 uM Compound 18 | 1.108 | 1.160 | 0.041 | 3.5% | 0.951 | 1.00 | 0.03 |
|  | 1.161 |  |  |  | 0.996 |  |  |
|  | 1.207 |  |  |  | 1.036 |  |  |
|  | 1.165 |  |  |  | 1.000 |  |  |
| 50 uM Compound 18 | 1.184 | 1.139 | 0.059 | 5.2% | 1.016 | 0.98 | 0.05 |
|  | 1.149 |  |  |  | 0.986 |  |  |
|  | 1.053 |  |  |  | 0.904 |  |  |
|  | 1.169 |  |  |  | 1.003 |  |  |
| 100 uM Compound 18 | 1.098 | 1.057 | 0.042 | 4.0% | 0.942 | 0.91 | 0.04 |
|  | 1.004 |  |  |  | 0.862 |  |  |
|  | 1.043 |  |  |  | 0.895 |  |  |
|  | 1.081 |  |  |  | 0.928 |  |  |
| 1 uM Compound 21 | 1.107 | 1.167 | 0.045 | 3.8% | 0.950 | 1.00 | 0.04 |
|  | 1.215 |  |  |  | 1.043 |  |  |
|  | 1.169 |  |  |  | 1.003 |  |  |
|  | 1.177 |  |  |  | 1.010 |  |  |
| 5 uM Compound 21 | 1.145 | 1.178 | 0.040 | 3.4% | 0.983 | 1.01 | 0.03 |
|  | 1.151 |  |  |  | 0.988 |  |  |
|  | 1.183 |  |  |  | 1.015 |  |  |
|  | 1.233 |  |  |  | 1.058 |  |  |
| 10 uM Compound 21 | 1.136 | 1.163 | 0.021 | 1.8% | 0.975 | 1.00 | 0.02 |
|  | 1.167 |  |  |  | 1.002 |  |  |
|  | 1.187 |  |  |  | 1.019 |  |  |
|  | 1.163 |  |  |  | 0.998 |  |  |
| 50 uM Compound 21 | 1.137 | 1.124 | 0.011 | 0.9% | 0.976 | 0.96 | 0.01 |
|  | 1.128 |  |  |  | 0.968 |  |  |
|  | 1.113 |  |  |  | 0.955 |  |  |
|  | 1.119 |  |  |  | 0.960 |  |  |
| 100 uM Compound 21 | 1.046 | 1.021 | 0.071 | 7.0% | 0.898 | 0.88 | 0.06 |
|  | 1.046 |  |  |  | 0.898 |  |  |
|  | 1.075 |  |  |  | 0.923 |  |  |
|  | 0.916 |  |  |  | 0.786 |  |  |

| Compound 17 0.5 uM | 0.241 | 0.233 | 0.012 | 5.3% | 1.082 | 1.04 | 0.06 |
| --- | --- | --- | --- | --- | --- | --- | --- |
|  | 0.231 |  |  |  | 1.037 |  |  |
|  | 0.216 |  |  |  | 0.970 |  |  |
|  | 0.243 |  |  |  | 1.091 |  |  |
| Compound 17 1 uM | 0.235 | 0.226 | 0.011 | 4.7% | 1.055 | 1.01 | 0.05 |
|  | 0.232 |  |  |  | 1.042 |  |  |
|  | 0.211 |  |  |  | 0.947 |  |  |
|  | 0.225 |  |  |  | 1.010 |  |  |
| Compound 17 5 uM | 0.201 | 0.222 | 0.017 | 7.8% | 0.902 | 1.00 | 0.08 |
|  | 0.215 |  |  |  | 0.965 |  |  |
|  | 0.233 |  |  |  | 1.046 |  |  |
|  | 0.239 |  |  |  | 1.073 |  |  |
| Compound 17 10 uM | 0.218 | 0.220 | 0.008 | 3.7% | 0.979 | 0.99 | 0.04 |
|  | 0.227 |  |  |  | 1.019 |  |  |
|  | 0.225 |  |  |  | 1.010 |  |  |
|  | 0.209 |  |  |  | 0.938 |  |  |
| Compound 17 100 uM | 0.197 | 0.208 | 0.009 | 4.2% | 0.884 | 0.93 | 0.04 |
|  | 0.209 |  |  |  | 0.938 |  |  |
|  | 0.218 |  |  |  | 0.979 |  |  |
|  | 0.206 |  |  |  | 0.925 |  |  |
| Compound 14 0.5 uM | 0.228 | 0.233 | 0.021 | 9.1% | 1.024 | 1.04 | 0.09 |
|  | 0.253 |  |  |  | 1.136 |  |  |
|  | 0.244 |  |  |  | 1.095 |  |  |
|  | 0.205 |  |  |  | 0.920 |  |  |
| Compound 14 1 uM | 0.248 | 0.231 | 0.019 | 8.3% | 1.113 | 1.03 | 0.09 |
|  | 0.227 |  |  |  | 1.019 |  |  |
|  | 0.205 |  |  |  | 0.920 |  |  |
|  | 0.242 |  |  |  | 1.086 |  |  |
| Compound 14 5 uM | 0.219 | 0.225 | 0.014 | 6.3% | 0.983 | 1.01 | 0.06 |
|  | 0.241 |  |  |  | 1.082 |  |  |
|  | 0.23 |  |  |  | 1.033 |  |  |
|  | 0.208 |  |  |  | 0.934 |  |  |
| Compound 14 10 uM | 0.206 | 0.211 | 0.014 | 6.7% | 0.925 | 0.95 | 0.06 |
|  | 0.193 |  |  |  | 0.866 |  |  |
|  | 0.225 |  |  |  | 1.010 |  |  |
|  | 0.218 |  |  |  | 0.979 |  |  |
| Compound 14 100 uM | 0.209 | 0.198 | 0.013 | 6.6% | 0.938 | 0.89 | 0.06 |
|  | 0.195 |  |  |  | 0.875 |  |  |
|  | 0.181 |  |  |  | 0.813 |  |  |
|  | 0.208 |  |  |  | 0.934 |  |  |
